# Eligibility for omecamtiv mecarbil in a real-world heart failure population: Data from the Swedish Heart Failure Registry

**DOI:** 10.1371/journal.pone.0303348

**Published:** 2024-05-24

**Authors:** Felix Lindberg, Natanael Øigaard, Marco Metra, Giuseppe M. C. Rosano, Ulf Dahlström, Peter Mol, Camilla Hage, Lars H. Lund, Gianluigi Savarese

**Affiliations:** 1 Division of Cardiology, Department of Medicine, Karolinska Institutet, Stockholm, Sweden; 2 Cardiology, Department of Medical and Surgical Specialties, Radiological Sciences and Public Health, Cardiothoracic Department, Civil Hospitals, University of Brescia, Brescia, Italy; 3 Cardiovascular Clinical Academic Group, Molecular and Clinical Research Institute, St George’s University Hospital, London, United Kingdom; 4 Cardiology, San Raffaele Cassino Hospital, Cassino, Italy; 5 Department of Cardiology and Department of Health, Medicine and Caring Sciences, Linköping University, Linköping, Sweden; 6 Department of Clinical Pharmacy and Pharmacology, University Medical Center Groningen, University of Groningen, Groningen, The Netherlands; 7 Heart and Vascular and Neuro Theme, Karolinska University Hospital, Stockholm, Sweden; Showa University: Showa Daigaku, JAPAN

## Abstract

**Aims:**

We assessed eligibility for omecamtiv mecarbil (OM) in a real-world cohort with heart failure with reduced ejection fraction (HFrEF) according to the selection criteria of the GALACTIC-HF trial (trial scenario) and selected trial´s criteria more likely to impact real-world use (pragmatic scenario).

**Methods and results:**

We included 31,015 patients with HFrEF lasting ≥3 months and registered in the Swedish HF registry between 2000–2021. Trial eligibility was calculated by applying all the GALACTIC-HF selection criteria. The pragmatic scenario considered only the New York Heart Association class, history of worsening HF, N-terminal pro-B-type natriuretic peptides (NT-proBNP), blood pressure and renal failure criteria defined as in the trial. Eligibility for OM in chronic HFrEF was 21% and 36% in the trial and pragmatic scenarios, respectively. Eligibility was higher in those with EF<30% (trial: 27%, pragmatic: 44%), in-patients (trial:30%, pragmatic:57%), severe HF (trial: 35%, pragmatic: 60%), NYHA class III-IV (trial: 26%, pragmatic: 45%), and NT-proBNP≥5,000pg/mL (trial: 30%, pragmatic: 51%). The criteria that most limited eligibility were history of a recent worsening HF event (60% eligible in chronic HFrEF), elevated NT-proBNP (82% eligible), and deviating blood pressure (82% eligible). Overall, eligible patients were characterized by more severe HF and higher CV event-rates in both scenarios, and higher comorbidity burden in the pragmatic scenario.

**Conclusion:**

Approximately 21% of real-world chronic HFrEF patients would be eligible for OM according to the GALACTIC-HF selection criteria, and 36% according to the criteria more likely to affect OM use in clinical practice. Criteria in both scenarios identified a patient-group with severe HF and high CV event-rates.

## Introduction

The prognosis in heart failure (HF) with reduced ejection fraction (HFrEF) remains poor despite recent advances in pharmacological treatment [1]. Current evidence-based pharmacotherapies in HFrEF might be poorly tolerated or contraindicated in patients with severe renal impairment, hyperkalemia, hypotension, and with worsening and advanced HF [2–4]. Meanwhile, inotropic agents, which might represent an option in part of these patients, have consistently failed to improve survival in randomized controlled trials (RCTs), instead showing signals of increased mortality and risk of arrhythmias [5–8].

Omecamtiv mecarbil (OM) is a novel oral myotrope that acts directly on the sarcomere, by increasing systolic ejection time and cardiac contractility without increasing oxygen demand or intracellular calcium transients [8, 9]. The Global Approach to Lowering Adverse Cardiac Outcomes through Improving Contractility in Heart Failure (GALACTIC-HF), a large multi-centre phase 3 double-blinded RCT enrolling 8,256 patients with EF ≤35% and a current or recent worsening HF event, demonstrated that OM reduced cardiovascular (CV) deaths or HF events by 8% compared to placebo, with an absolute risk reduction of ~2% [10]. Importantly, OM did not affect renal function, potassium levels, or blood pressure. Therefore, although the relative risk reduction was modest, OM might represent one of few viable alternatives for patients with contraindications or low tolerance to established HFrEF therapies, and in those with advanced HF where the available therapeutic options are limited [11].

RCTs in HF apply eligibility criteria to ensure the selection of the intended patient population, to enrich for the CV events targeted by the intervention, and to minimize safety events and the effect of competing risk from non-CV events [12–14]. A comprehensive characterization of the eligibility for OM according to the GALACTIC-HF selection criteria can provide important information on the generalizability of the trial´s findings, design of future studies on OM, decision-making for regulatory and reimbursement purposes, and potential clinical implementation.

We aimed to assess i) the proportion of patients eligible for OM in a large, real-world HF cohort with HFrEF; ii) compare patient characteristics and outcomes according to eligibility for OM, as defined by the enrolment criteria of GALACTIC-HF (*trial scenario*) and the criteria deemed most likely to affect the use of OM in clinical practice (*pragmatic scenario*).

## Methods

### Data sources

The ongoing nationwide Swedish HF registry (SwedeHF) has been previously described [15]. Since its foundation in May 2000, SwedeHF has enrolled patients who fulfill the only inclusion criterion of clinician-judged HF (since 2017 defined as International Classification of Diseases, 10th Revision [ICD-10] codes I50.0, I50.1, I50.9, I42.0, I42.6, I42.7, I25.5, I11.0, I13.0, I13.2). Upon the outpatient encounter or hospital discharge prompting registration, i.e. index date, ~80 variables, including EF, New York Heart Association (NYHA) functional class, and N-terminal pro-B-type natriuretic peptide (NT-proBNP), are collected. As of 2021, the nationwide coverage of prevalent HF was 32% [16]. For this study, the data in SwedeHF was supplemented by linkage with other registries: the National Patient Register provided additional comorbidities and hospitalizations; Statistics Sweden provided socioeconomic data; the Cause of Death Register provided date and cause of death. The establishment of SwedeHF, its linkage with other registries, and the execution of the present study were all approved by the Swedish Ethical Review Authority. Although written consent was not required for enrolment in SwedeHF, patients were informed of entry and able to opt-out. All data were pseudonymized before being delivered to investigators, who did not access data that could be used to identify individual patients. Prospective interventional trials that use SwedeHF as a platform are registered in trial registries (e.g. SPIRRIT: NCT02901184). The present study was not specifically registered, since it was a retrospective analysis of already collected data and did not involve prospective patient recruitment, allocation of an intervention, or collection of new data.

### Study population and design

Patients enrolled in SwedeHF from 1^st^ May 2000 to 31^st^ December 2021 with non-missing entry for EF were eligible for this study. To reflect more contemporary care, the most recent entry for each patient was considered, with the date of the hospital discharge/out-patient encounter that prompted that entry defined as baseline. Patients with EF≥40% were excluded. In SwedeHF, reported EF refers to the last available measurement at any given time, which may have been collected prior to the out-patient visit or hospital discharge that prompts the SwedeHF entry. To provide time for patients to have been initiated on HF pharmacotherapy prior to recording of EF, we therefore only included entries that occurred >3 months since the diagnosis of HF. GALACTIC-HF enrolled patients with EF≤35% [10]. In SwedeHF, EF is reported as a categorical variable (<30%, 30–39%, 40–49%, and ≥50%) in most patients, which does not enable the adoption of a 35% cut-off. Therefore, the main analysis considered patients with EF<40%, whereas a sensitivity analysis was performed considering only those with EF<30%. In addition, six sub-cohorts were assessed: i) patients with EF<30%; ii) in-patients who were hospitalized for HF at baseline; iii) out-patients; iv) patients with severe HF (defined as EF<30%, NYHA class III-IV, and NT-proBNP≥5,000pg/mL); v) NYHA class III-IV; vi) NT-proBNP≥5,000pg/mL. Patients were followed-up until 31^st^ December 2021, emigration from Sweden, or a death (whichever came first). The patient selection is depicted in S1 Fig in the **S1 Appendix**.

### Eligibility criteria for OM in the trial and pragmatic scenarios

Eligibility was assessed according to two main scenarios: 1) the *trial scenario* considered all eligibility criteria of the GALACTIC-HF trial; 2) in the *pragmatic scenario*, key trial criteria deemed by the investigators as more likely to determine potential clinical use were selected and defined as in the trial (inclusion criteria: NYHA class II-IV, history of worsening HF, elevated NT-proBNP; exclusion criteria: deviating blood pressure/heart rate, impaired renal function).

Each eligibility criterion from the GALACTIC-HF trial was reviewed and adapted to the setting of SwedeHF and the linked registries as reported in S1 Table in the **S1 Appendix**. Several sensitivity analyses were performed to assess the impact of different adaptions of criteria to the real-world setting. GALACTIC-HF enrolled patients who received optimal medical therapy (OMT) unless contraindicated [10]. Two analyses were performed considering different definitions of OMT. In the main analysis, all patients with a HF duration ≥3 months were considered as fulfilling the OMT criterion, under the assumption that if OMT had not been achieved by then, it might be due to non-tolerance or contraindication. As a sensitivity analysis, we considered only patients with concomitant use of a renin-angiotensin system inhibitors (RASi)/angiotensin receptor-neprilysin inhibitor (ARNi), beta-blocker and MRA at baseline as fulfilling the OMT criterion. GALACTIC-HF excluded patients with a systolic blood pressure >140 mmHg or <85 mmHg. However, OM, unlike most HF therapies, does not decrease blood pressure, and might even be more beneficial in patients with lower blood pressure according to post-hoc analyses of GALACTIC-HF [17]. Therefore, we performed an additional sensitivity analysis in both scenarios where a systolic blood pressure <85 mmHg did not lead to ineligibility. In GALACTIC-HF, atrial fibrillation at baseline was associated with less benefit of OM [10, 18], and post-hoc analyses suggested that this association was particularly pronounced in patients with atrial fibrillation and digoxin use [18]. We therefore considered two additional sensitivity analyses in both scenarios: one where patients with both atrial fibrillation and digoxin use at baseline were considered as ineligible, and one where all patients with atrial fibrillation were considered as ineligible.

### Statistical analysis

Eligibility was calculated as the proportion of patients who were eligible after applying all inclusion and exclusion criteria of the trial and pragmatic scenarios, respectively, in the seven study cohorts (all having HF duration ≥3 months): patients with EF<40% (main analysis); patients with EF<30%; in-patients hospitalized for HF at baseline; out-patients; patients with severe HF; NYHA class III-IV; NT-proBNP≥5,000pg/mL. The impact of each criterion was estimated by calculating the eligibility after applying the respective criterion individually (not sequentially). Categorical and continuous baseline characteristics were described as frequencies (percentages) and median (interquartile range [IQR]) and compared according to eligibility status by χ2 test and Kruskal-Wallis test, respectively.

Seven outcomes were considered: CV hospitalization, non-CV hospitalization, hospitalization for stroke/transient ischemic attack (TIA), HF hospitalization, all-cause hospitalization, non-CV death, CV death and all-cause death according to eligibility status. Poisson regression was used to calculate incidence rates (per 100 patient-years), and perform comparisons according to eligibility status, reported as incidence rate ratios (IRR) with 95% confidence interval (CI).

Missing data were handled by single imputation (R package *mice*) [19]. The imputation model included variables labelled with * in **Table 1** along with all-cause mortality as a Nelson-Aalen estimator. Two consistency analyses were performed to assess the potential bias from missing data: 1) patients were excluded if any of the variables needed for eligibility estimation was missing (*complete-case*); 2) any criterion was considered as fulfilled if a variable needed to compute eligibility for that criterion was missing (*missing-as-eligible*). All analyses were performed in R 4.0.2. Two-sided p-values <0.05 were considered statistically significant.

**Table 1:** Baseline characteristics according to eligibility for omecamtiv mecarbil (trial and pragmatic scenarios) in the overall (ejection fraction <40%) cohort.

	Trial scenario			Pragmatic scenario		
Criterion	Ineligible	Eligible	*P*	Ineligible	Eligible	*P*
*Number of patients (% of study population)*	*24*,*476 (78*.*9%)*	*6*,*539 (21*.*1%)*	* *	*19*,*974 (64*.*4%)*	*11*,*041 (35*.*6%)*	* *
**Sociodemographic variables**		* *	* *	* *	* *	* *
Female*	6,541 (26.7%)	1,742 (26.6%)	0.904	5,283 (26.4%)	3,000 (27.2%)	0.173
Age, years	76 [68.0, 83.0]	76 [69.0, 81.0]	<0.001	75 [67.0, 81.0]	78 [70.0, 83.0]	<0.001
Age ≥75 years*	13,351 (54.5%)	3,653 (55.9%)	0.059	10,235 (51.2%)	6,769 (61.3%)	<0.001
Index year*			<0.001			<0.001
2000–2008	3,711 (15.2%)	1,294 (19.8%)		2,866 (14.3%)	2,139 (19.4%)	
2009–2015	8,759 (35.8%)	2,925 (44.7%)		6,682 (33.5%)	5,002 (45.3%)	
2016–2021	12,006 (49.1%)	2,320 (35.5%)		10,426 (52.2%)	3,900 (35.3%)	
Income level <median (by index year)*	12,120 (49.5%)	3,360 (51.4%)	0.007	9,674 (48.5%)	5,806 (52.6%)	<0.001
Education: compulsory school only (vs. secondary school/university)*	10,344 (43.1%)	2,922 (45.8%)	<0.001	8,193 (41.8%)	5,073 (47.0%)	<0.001
Single living*	11,632 (47.6%)	3,001 (45.9%)	0.021	9,140 (45.8%)	5,493 (49.8%)	<0.001
Children*	20,407 (83.4%)	5,384 (82.3%)	0.048	16,682 (83.5%)	9,109 (82.5%)	0.023
**Health organizational variables**	* *	* *	* *	* *	* *	* *
Caregiver: in-patient (vs. out-patient)*	7,084 (28.9%)	3,012 (46.1%)	<0.001	4,328 (21.7%)	5,768 (52.2%)	<0.001
Planned follow-up: specialty care (vs. primary care/other)*	16,219 (69.4%)	4,457 (71.7%)	0.001	13,935 (72.7%)	6,741 (64.8%)	<0.001
Referral to follow-up in a nurse-led HF unit*	13,788 (59.8%)	3,433 (56.0%)	<0.001	11,978 (63.3%)	5,243 (51.1%)	<0.001
**Clinical variables**	* *	* *	* *	* *	* *	* *
Ejection fraction <30%	10,798 (44.1%)	4,007 (61.3%)	<0.001	8,312 (41.6%)	6,493 (58.8%)	<0.001
NYHA class*			<0.001			<0.001
I	1,952 (10.0%)	0 (0.0%)		1,952 (11.9%)	0 (0.0%)	
II	8,469 (43.6%)	1,919 (37.4%)		7,425 (45.4%)	2,963 (36.0%)	
III	8,205 (42.2%)	2,833 (55.3%)		6,414 (39.3%)	4,624 (56.2%)	
IV	811 (4.2%)	375 (7.3%)		548 (3.4%)	638 (7.8%)	
HF duration ≥6 months*	19,781 (82.1%)	5,271 (81.4%)	0.203	16,113 (82.1%)	8,939 (81.8%)	0.6
Systolic blood pressure, mmHg	120 [110.0, 137.0]	115 [105.0, 125.0]	<0.001	121 [110.0, 140.0]	115 [105.0, 126.0]	<0.001
Diastolic blood pressure, mmHg	70 [62.0, 80.0]	70 [60.0, 76.0]	<0.001	70 [64.0, 80.0]	70 [60.0, 76.0]	<0.001
Mean arterial pressure, mmHg	88.3 [80.0, 96.7]	83.3 [76.7, 91.7]	<0.001	90 [80.0, 98.3]	83.3 [76.7, 91.7]	<0.001
Mean arterial pressure <90 mmHg*	12,415 (52.6%)	4,349 (68.1%)	<0.001	9,463 (49.3%)	7,301 (67.6%)	<0.001
Heart rate, b.p.m.	70 [62.0, 80.0]	72 [64.0, 81.0]	<0.001	70 [62.0, 80.0]	72 [65.0, 82.0]	<0.001
Heart rate ≥70 b.p.m.*	12,831 (55.6%)	3,822 (61.7%)	<0.001	10,031 (53.4%)	6,622 (63.1%)	<0.001
Body mass index, kg/m^2^	26 [23.0, 29.7]	26 [23.1, 29.9]	0.731	26.4 [23.4, 30.1]	25.5 [22.5, 29.2]	<0.001
Body mass index category*			0.038			<0.001
Normal/overweight	15,694 (73.3%)	4,367 (71.7%)		12,369 (71.8%)	7,692 (74.8%)	
Underweight	633 (3.0%)	203 (3.3%)		428 (2.5%)	408 (4.0%)	
Obese	5,085 (23.7%)	1,517 (24.9%)		4,421 (25.7%)	2,181 (21.2%)	
HF hospitalization past 6 months	10,692 (43.7%)	5,756 (88.0%)	<0.001	6,545 (32.8%)	9,903 (89.7%)	<0.001
HF hospitalization past 12 months*	11,905 (48.6%)	6,539 (100.0%)	<0.001	7,403 (37.1%)	11,041 (100.0%)	<0.001
**Laboratory variables**	* *	* *	* *	* *	* *	* *
NT-proBNP, pg/L	2,090 [820.0, 5412.5]	4,250 [2131.0, 8517.0]	<0.001	1,670 [679.8, 4189.2]	4,790 [2330.8, 9948.0]	<0.001
NT-proBNP tertile (by EF and atrial fibrillation)*			<0.001			<0.001
NT-proBNP lowest tertile	5,414 (38.5%)	472 (13.1%)		5,179 (44.5%)	707 (11.8%)	
NT-proBNP middle tertile	4,469 (31.8%)	1,417 (39.4%)		3,697 (31.7%)	2,189 (36.4%)	
NT-proBNP highest tertile	4,176 (29.7%)	1,712 (47.5%)		2,772 (23.8%)	3,116 (51.8%)	
eGFR, mL/min/1.73m^2^	60.4 [43.1, 80.7]	55.6 [41.1, 74.3]	<0.001	62.5 [44.8, 82.5]	54.2 [39.8, 72.7]	<0.001
eGFR category*			<0.001			<0.001
eGFR≥60	12,025 (50.6%)	2,775 (43.0%)		10,366 (53.6%)	4,434 (40.7%)	
eGFR 30–60	9,477 (39.8%)	3,099 (48.0%)		7,172 (37.1%)	5,404 (49.6%)	
eGFR<30	2,282 (9.6%)	577 (8.9%)		1,802 (9.3%)	1,057 (9.7%)	
Hemoglobin, g/L	132 [120, 144]	131 [119, 143]	<0.001	134 [122, 145]	129 [117, 141]	<0.001
Potassium, mmol/L	4.3 [4.0, 4.6]	4.2 [3.9, 4.5]	<0.001	4.3 [4.0, 4.6]	4.2 [3.9, 4.5]	<0.001
Potessium category			<0.001			<0.001
Hypokalemia <5mmol/L	628 (3.1%)	218 (4.3%)		426 (2.6%)	420 (4.9%)	
Normokalemia 3.5–4.9mmol/L	18,033 (89.8%)	4,505 (88.5%)		14,969 (90.2%)	7,569 (88.3%)	
Hyperkalemia ≥5mmol/L	1,419 (7.1%)	368 (7.2%)		1,203 (7.2%)	584 (6.8%)	
**Comorbidities**	* *	* *	* *	* *	* *	* *
Peripheral arterial disease*	2,774 (11.3%)	847 (13.0%)	<0.001	2,237 (11.2%)	1,384 (12.5%)	<0.001
Stroke/transitory ischemic attack*	4,757 (19.4%)	1,241 (19.0%)	0.416	3,644 (18.2%)	2,354 (21.3%)	<0.001
Anemia*	8,227 (37.0%)	2,427 (39.5%)	<0.001	6,124 (34.2%)	4,530 (43.3%)	<0.001
Cancer past 3 years*	3,713 (15.2%)	350 (5.4%)	<0.001	2,570 (12.9%)	1,493 (13.5%)	0.105
Liver disease*	726 (3.0%)	126 (1.9%)	<0.001	467 (2.3%)	385 (3.5%)	<0.001
Major bleeding*	5,062 (20.7%)	1,375 (21.0%)	0.551	3,836 (19.2%)	2,601 (23.6%)	<0.001
Diabetes mellitus*	7,576 (31.0%)	2,538 (38.8%)	<0.001	6,158 (30.8%)	3,956 (35.8%)	<0.001
Atrial fibrillation*	14,557 (59.5%)	4,095 (62.6%)	<0.001	11,584 (58.0%)	7,068 (64.0%)	<0.001
Hypertension*	16,179 (66.1%)	4,270 (65.3%)	0.23	13,213 (66.2%)	7,236 (65.5%)	0.281
Chronic obstructive pulmonary disease*	3,517 (14.4%)	1,221 (18.7%)	<0.001	2,753 (13.8%)	1,985 (18.0%)	<0.001
Ischemic heart disease*	16,166 (66.0%)	4,517 (69.1%)	<0.001	13,022 (65.2%)	7,661 (69.4%)	<0.001
Revascularized	9,988 (40.8%)	2,853 (43.6%)	<0.001	8,256 (41.3%)	4,585 (41.5%)	0.75
Valvular disease*	6,992 (28.6%)	2,154 (32.9%)	<0.001	5,323 (26.6%)	3,823 (34.6%)	<0.001
**Treatments**	* *	* *	* *	* *	* *	* *
Beta-blockers*	22,351 (91.6%)	6,073 (93.1%)	<0.001	18,368 (92.2%)	10,056 (91.4%)	0.01
RASi/ARNi*	21,450 (88.4%)	5,809 (89.6%)	0.004	17,866 (90.1%)	9,393 (86.0%)	<0.001
MRA*	11,551 (47.4%)	3,486 (53.6%)	<0.001	9,555 (48.0%)	5,482 (50.0%)	0.001
RASi/ARNi + Beta-blocker + MRA	9,946 (40.9%)	3,023 (46.6%)	<0.001	8,422 (42.4%)	4,547 (41.5%)	0.144
SGLT2 inhibitor*^a^	608 (33.6%)	123 (41.4%)	0.01	548 (33.6%)	183 (38.3%)	0.067
Diuretics*	19,281 (79.1%)	5,979 (91.9%)	<0.001	15,147 (76.1%)	10,113 (92.1%)	<0.001
Digoxin*	3,292 (13.5%)	1,176 (18.0%)	<0.001	2,600 (13.1%)	1,868 (17.0%)	<0.001
Nitrates*	3,907 (16.0%)	1,043 (16.0%)	0.999	3,007 (15.1%)	1,943 (17.7%)	<0.001
Anticoagulants*	12,111 (49.7%)	3,656 (56.2%)	<0.001	10,045 (50.5%)	5,722 (52.1%)	0.007
Antiplatelets*	10,122 (41.5%)	2,478 (38.1%)	<0.001	8,166 (41.0%)	4,434 (40.4%)	0.26
Statins*	13,213 (54.2%)	3,671 (56.3%)	0.002	11,304 (56.8%)	5,580 (50.7%)	<0.001
Cardiac resynchronisation therapy*	2,833 (11.7%)	1,012 (15.5%)	<0.001	2,418 (12.2%)	1,427 (13.0%)	0.045
Implantable cardioverter-defibrillator*	3,694 (15.2%)	1,120 (17.2%)	<0.001	3,193 (16.1%)	1,621 (14.8%)	0.002

Descriptive statistics based on unimputed data.

^a^ SGLT2 inhibitor use reflects only those in whom use / non-use was reported, which comprised only a small minority of the enrolled patients.

**Abbreviations:** ARNi, angiotensin receptor–neprilysin inhibitor; b.p.m., beats per minute; eGFR, estimated glomerular filtration rate (calculated by the Chronic Kidney Disease Epidemiology Collaboration formula); HF, heart failure; MRA, mineralocorticoid receptor antagonist; NT-proBNP, N-terminal pro-B-type natriuretic peptide; NYHA, New York Heart Association; RASi, renin–angiotensin system inhibitor; SGLT2, sodium-glucose co-transporter

## Results

In 31,015 unique patients with EF<40% and HF duration ≥3 months, the median age was 76 (IQR 68–82) years, 27% were female, 50% had NYHA III-IV, and the median NT-proBNP was 2,510 (IQR 999–6,150) pg/mL. The use of beta-blockers was 92%, RASi/ARNi 89%, MRA 49%, and diuretics 82%. The most predominant comorbidities were ischemic heart disease (67%), hypertension (66%), and atrial fibrillation (60%).

The cohort with EF<30% (n = 14,804 [48%]) was similar for sex, age, treatments, and comorbidities, but had overall more severe HF (i.e. higher NYHA classes and NT-proBNP). The cohorts including only in-patients (n = 10,096 [33%]) and patients with severe HF (i.e. EF<30%, NYHA III-IV, and NT-proBNP≥5,000pg/mL) (n = 5,631 [18%]) were older, and had markedly higher NYHA classes, NT-proBNP, and were more likely to have peripheral artery disease, stroke/TIA, diabetes, atrial fibrillation, ischemic heart disease, and valvular disease, as compared with the overall (EF<40%) and EF<30% cohorts. The characteristics of the seven cohorts (EF<40%, EF<30%, in-patients, out-patients, severe HF, NYHA III-IV, and NT-proBNP≥5,000pg/mL) are reported in S2 Table in the **S2 Appendix**.

### Eligibility for OM

**Fig 1:**
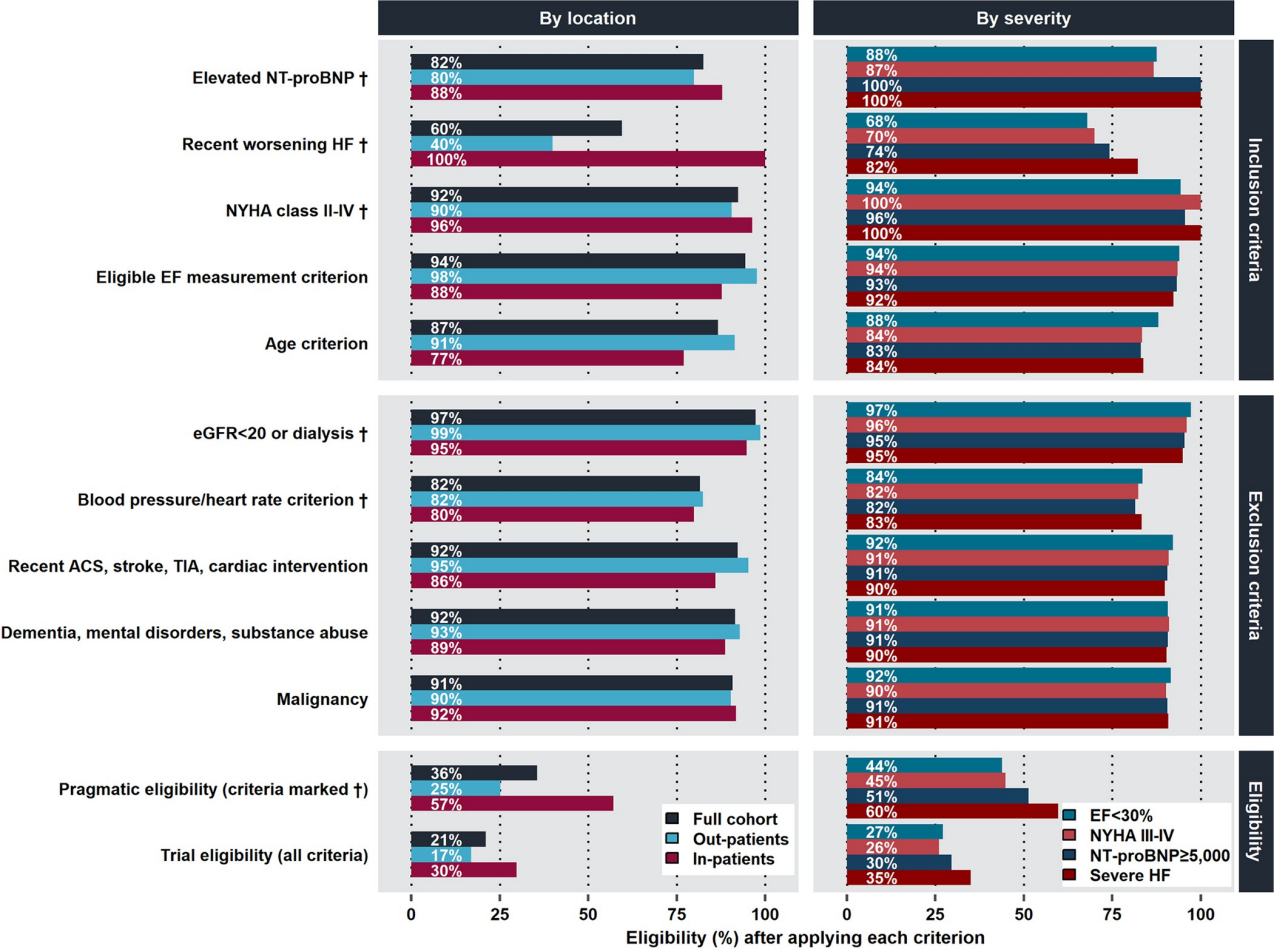
Impactful eligibility criteria in the trial and pragmatic scenarios in the overall cohort (EF<40%) and subgroups (EF<30%, in-patients, out-patients, severe HF, NYHA III-IV, and NT-proBNP≥5,000pg/mL). **Abbreviations:** ACS, acute coronary syndrome; EF, ejection fraction; eGFR, estimated glomerular filtration rate; HF, heart failure; NT-proBNP, N-terminal pro-B-type natriuretic peptide; NYHA, New York Heart Association; TIA, transient ischemic attack.

In the overall cohort (EF<40%), 21% of patients met all the eligibility criteria in the trial scenario (**Fig 1**). Inclusion criteria had a stronger impact on eligibility (only 38% of patients met all inclusion criteria) than exclusion criteria (57% of patients were not excluded by any exclusion criteria). The most limiting inclusion criteria were recent worsening HF event (60% eligible), elevated NT-proBNP (83%) and age<85 years (87%). The exclusion criteria that most limited eligibility were deviating (i.e. too low or too high) blood pressure or heart rate (82% eligible), malignancy (91%), dementia/substance abuse (92%), and recent acute coronary syndrome, stroke/TIA, or cardiac intervention (92%). Eligibility according to the pragmatic scenario was 36%. The most impactful criteria were the same as in the trial scenario, i.e. recent worsening HF event, elevated NT-proBNP, and deviating blood pressure or heart rate.

Eligibility was higher in the cohorts with EF<30% (trial: 27%, pragmatic: 44%), in-patients (trial: 30%, pragmatic: 57%), severe HF (trial: 35%, pragmatic: 60%), NYHA class III-IV (trial: 26%, pragmatic: 45%), and NT-proBNP≥5,000pg/mL (trial: 30%, pragmatic: 51%) when compared to the overall cohort (trial: 21%, pragmatic: 36%). This was mainly driven by a higher proportion of patients in these cohorts, as compared with the overall cohort, meeting the criteria of recent worsening HF event and elevated NT-proBNP. Out-patients had lower eligibility (trial: 17%; pragmatic 25%) than the overall cohort, mainly driven by lower eligibility for the criterion of a recent worsening HF event (40%). The eligibility rates for all criteria across the different scenarios and in each cohort are presented in **Table 2**.

**Table 2:** Eligibility for omecamtiv mecarbil according to all the selection criteria in the trial and pragmatic scenarios. All numbered criteria were applied in the trial scenario. Only criteria with **bold underlined font** were applied in the pragmatic scenario.

Criterion	EF<40%	EF<30%	In-patients	Out-patients	Severe HF	NYHA III-IV	NT-proBNP >5000
*Number of patients (% of study population)*	*31*,*015 (100%)*	*14*,*805 (47*.*7%)*	*10*,*096 (32*.*6%)*	*20*,*919 (67*.*4%)*	*5*,*631 (18*.*2%)*	*16*,*008 (51*.*6%)*	*12*,*077 (38*.*9%)*
**Inclusion**	* *	* *	* *	* *	* *	* *	* *
1. Informed consent (assumed 100%)	100.0%	100.0%	100.0%	100.0%	100.0%	100.0%	100.0%
2. Male/female, 18–85 years	86.7%	88.0%	77.0%	91.4%	83.8%	83.5%	83.1%
**3. Requiring HF treatment ≥30 days (assumed 100% in patients with HF duration ≥3 months)**	100.0%	100.0%	100.0%	100.0%	100.0%	100.0%	100.0%
4. EF measurement not within 30 days of HF debute or event likely to affect EF	94.4%	94.0%	87.8%	97.7%	92.3%	93.5%	93.2%
**5. NYHA class II-IV**	92.4%	94.4%	96.3%	90.5%	100.0%	100.0%	95.6%
6. Receiving OMT, unless contraindicated (assumed 100% in patients with HF duration ≥3 months)	100.0%	100.0%	100.0%	100.0%	100.0%	100.0%	100.0%
. OMT sensitivity: OMT defined as concomitant use of RASi/ARNi+BB+MRA	42.2%	44.6%	30.8%	47.8%	37.0%	39.0%	36.0%
**7. Currently hospitalized for HF or experienced worsening HF event in the past 1 year**	59.5%	68.0%	100.0%	39.9%	82.2%	70.0%	74.2%
**8. Elevated natriuretic peptides ≥400pg/mL or ≥1,200pg/mL in patients with AF**	82.5%	87.5%	87.9%	79.9%	100.0%	86.7%	100.0%
*All inclusion criteria trial scenario*	*37*.*6%*	*46*.*4%*	*57*.*8%*	*27*.*8%*	*62*.*4%*	*46*.*7%*	*53*.*6%*
*All inclusion criteria trial scenario*, *OMT sensitivity*	*16*.*2%*	*21*.*2%*	*20*.*1%*	*14*.*4%*	*25*.*0%*	*19*.*1%*	*20*.*8%*
*All inclusion criteria pragmatic scenario*	*45*.*3%*	*54*.*0%*	*74*.*9%*	*31*.*0%*	*75*.*2%*	*57*.*1%*	*65*.*9%*
**Exclusion**	* *	* *	* *	* *	* *	* *	* *
1. Receiving other investigational device/treatment (assumed 100%)	100.0%	100.0%	100.0%	100.0%	100.0%	100.0%	100.0%
2. Malignancy within 5 years prior	90.8%	91.5%	91.7%	90.3%	90.8%	90.2%	90.6%
3. Known sensitivity to OM (assumed 100%)	100.0%	100.0%	100.0%	100.0%	100.0%	100.0%	100.0%
4. Condition likely to interfer, e.g. dementia, mental disorders, substance abuse	91.5%	90.7%	88.7%	92.9%	90.4%	91.1%	90.7%
5. Inability to swallow (assumed 100%)	100.0%	100.0%	100.0%	100.0%	100.0%	100.0%	100.0%
6. mechanical hemodynamical support or invasive ventilation past 7 days(assumed 100%)	100.0%	100.0%	100.0%	100.0%	100.0%	100.0%	100.0%
7. IV inotropes or vasopressors past 3 days (assumed 100%)	100.0%	100.0%	100.0%	100.0%	100.0%	100.0%	100.0%
8. IV diuretics or vasodilators, oxygen therapy, NIV, or CPAP, in past 12 hours (assumed 100%)	100.0%	100.0%	100.0%	100.0%	100.0%	100.0%	100.0%
9. ACS, stroke/TIA, or major cardiac surgery/intervention past 3 months	92.2%	92.2%	85.9%	95.3%	89.9%	90.9%	90.6%
10. Insertion of other cardiac devices past 1 month	97.5%	97.1%	94.8%	98.8%	96.9%	97.4%	97.2%
11. Uncorrected valvulopathy, HCM/infiltrative cardiomyopathy, myocarditis, congenital heart disease	96.8%	96.9%	96.9%	96.7%	96.7%	96.7%	96.8%
12. Untreated severe ventricular arrhythmia (assumed 100%)	100.0%	100.0%	100.0%	100.0%	100.0%	100.0%	100.0%
13. Chronic antiarrhythmics, excluding amiodarone/digoxin/calcium blocker/BB (assumed 100%)	100.0%	100.0%	100.0%	100.0%	100.0%	100.0%	100.0%
14. Symptomatic bradycardia or 2nd-3rd degree heart block without pacemaker (assumed 100%)	100.0%	100.0%	100.0%	100.0%	100.0%	100.0%	100.0%
15. Routine outpatient IV infusions for HF (assumed 100%)	100.0%	100.0%	100.0%	100.0%	100.0%	100.0%	100.0%
**16. Systolic BP not 85-140mmHg, diastolic BP >90mmHg, or heart rate not 50–110 b.p.m.**	81.6%	83.6%	79.9%	82.4%	83.3%	82.4%	81.5%
**. BP sensitivity: Systolic BP<85mmHg does not lead to exclusion**	83.5%	86.1%	82.6%	84.0%	87.0%	85.1%	84.2%
**17. eGFR<20mL/min/1.73m2, or patient on renal dialysis**	97.3%	97.2%	94.8%	98.6%	94.9%	96.0%	95.4%
18. Hepatic impairment	99.1%	98.8%	98.1%	99.6%	98.3%	98.9%	98.7%
19. Previously received OM (assumed 100%)	100.0%	100.0%	100.0%	100.0%	100.0%	100.0%	100.0%
20. Non-CV comorbidity reducing life expectancy to <2 years (assumed 100%)	100.0%	100.0%	100.0%	100.0%	100.0%	100.0%	100.0%
21. Major organ transplant or planned chronic mechanical support / heart transplant	99.3%	99.2%	99.3%	99.3%	99.1%	99.2%	99.1%
22. Patient or patient’s partner of childbearing potential (assumed 100%)	100.0%	100.0%	100.0%	100.0%	100.0%	100.0%	100.0%
23. Current or planned pregnancy / breastfeeding (assumed 100%)	100.0%	100.0%	100.0%	100.0%	100.0%	100.0%	100.0%
24. Planned discharge to long term care facility or hospice (assumed 100%)	100.0%	100.0%	100.0%	100.0%	100.0%	100.0%	100.0%
25. Other condition likely to interfere with safety or ability to adhere (assumed 100%)	100.0%	100.0%	100.0%	100.0%	100.0%	100.0%	100.0%
**AF+digoxin sensitivity: Patients with AF with digoxin use are considered ineligible**	**86.8%**	**85.2%**	**83.8%**	**88.3%**	**83.9%**	**85.5%**	**85.2%**
**AF sensitivity: All patients with AF are considered ineligible**	**39.9%**	**39.8%**	**32.7%**	**43.3%**	**33.2%**	**33.9%**	**35.9%**
*All exclusion criteria trial scenario*	*56*.*7%*	*57*.*5%*	*47*.*2%*	*61*.*3%*	*53*.*5%*	*55*.*2%*	*53*.*6%*
*All exclusion criteria trial scenario*, *BP sensitivity*	*58*.*0%*	*59*.*1%*	*48*.*9%*	*62*.*4%*	*55*.*9%*	*57*.*0%*	*55*.*4%*
*All exclusion criteria trial scenario*, *AF+digoxin sensitivity*	*49*.*0%*	*48*.*8%*	*38*.*6%*	*54*.*0%*	*44*.*5%*	*46*.*8%*	*45*.*3%*
*All exclusion criteria trial scenario*, *AF sensitivity*	*22*.*3%*	*22*.*5%*	*13*.*9%*	*26*.*3%*	*17*.*0%*	*18*.*0%*	*18*.*6%*
*All exclusion criteria pragmatic scenario*	*79*.*7%*	*81*.*4%*	*76*.*3%*	*81*.*3%*	*79*.*5%*	*79*.*5%*	*78*.*1%*
*All exclusion criteria pragmatic scenario*, *BP sensitivity*	*81*.*5%*	*83*.*7%*	*78*.*7%*	*82*.*8%*	*82*.*8%*	*82*.*1%*	*80*.*5%*
*All exclusion criteria pragmatic scenario*, *AF+digoxin sensitivity*	*68*.*9%*	*69*.*2%*	*63*.*4%*	*71*.*6%*	*66*.*3%*	*67*.*8%*	*66*.*3%*
*All exclusion criteria pragmatic scenario*, *AF sensitivity*	*31*.*3%*	*32*.*0%*	*24*.*4%*	*34*.*6%*	*26*.*0%*	*26*.*4%*	*27*.*6%*
**Eligibility**	* *	* *	* *	* *	* *	* *	* *
*All criteria trial scenario*	*21*.*1%*	*27*.*1%*	*29*.*8%*	*16*.*9%*	*35*.*0%*	*26*.*1%*	*29*.*5%*
*All criteria trial scenario*, *OMT sensitivity*	*9*.*8%*	*13*.*1%*	*11*.*2%*	*9*.*2%*	*14*.*4%*	*11*.*5%*	*12*.*2%*
*All criteria trial scenario*, *BP sensitivity*	*21*.*8%*	*28*.*2%*	*31*.*1%*	*17*.*4%*	*37*.*0%*	*27*.*2%*	*30*.*7%*
*All criteria trial scenario*, *AF+digoxin sensitivity*	*17*.*7%*	*22*.*7%*	*24*.*2%*	*14*.*6%*	*28*.*6%*	*21*.*8%*	*24*.*3%*
*All criteria trial scenario*, *AF sensitivity*	*7*.*9%*	*10*.*7%*	*9*.*6%*	*7*.*0%*	*10*.*9%*	*8*.*7%*	*9*.*7%*
*All criteria pragmatic scenario*	*35*.*6%*	*43*.*9%*	*57*.*1%*	*25*.*2%*	*59*.*7%*	*44*.*8%*	*51*.*3%*
*All criteria pragmatic scenario*, *BP sensitivity*	*36*.*8%*	*45*.*5%*	*59*.*1%*	*26*.*0%*	*62*.*6%*	*46*.*6%*	*53*.*3%*
*All criteria pragmatic scenario*, *AF+digoxin sensitivity*	*30*.*1%*	*36*.*9%*	*47*.*4%*	*21*.*8%*	*49*.*5%*	*37*.*8%*	*43*.*0%*
*All criteria pragmatic scenario*, *AF sensitivity*	*12*.*8%*	*16*.*9%*	*18*.*3%*	*10*.*1%*	*18*.*6%*	*14*.*6%*	*16*.*8%*

**Abbreviations:** AF, atrial fibrillation; ACS, acute coronary syndrome; ARNi, angiotensin receptor–neprilysin inhibitor; BB, beta-blocker; BP, blood pressure; CV, cardiovascular; CPAP, continuous positive airway pressure; EF, ejection fraction; eGFR, estimated glomerular filtration rate; HCM, hypertrophic cardiomyopathy; HF, heart failure; MRA, mineralocorticoid receptor antagonist; NIV, non-invasive ventilation; NT-proBNP, N-terminal pro-B-type natriuretic peptide; NYHA, New York Heart Association; OM, omecamtiv mecarbil; OMT, optimal medical therapy; RASi, renin–angiotensin system inhibitor; TIA, transient ischemic attack

In the trial scenario, in the sensitivity analysis considering a literal interpretation of the OMT criterion (i.e. only patients with concomitant use of RASi/ARNi, beta-blocker and MRA), only 42% of patients in the overall cohort fulfilled the OMT criterion, and overall eligibility was markedly lower (10% vs. 21%) than in the main analysis, where OMT was assumed as fulfilled. In the sensitivity analysis considering as eligible also patients with systolic blood pressure <85mmHg, overall eligibility was only modestly higher (trial: 22% vs. 21%; pragmatic: 37% vs. 36%) than in the main analysis, where systolic blood pressure <85mmHg led to ineligibility. Eligibility was modestly lower when classifying as ineligible those with atrial fibrillation and digoxin use (trial: 18% vs. 21%; pragmatic: 30% vs. 36%), and markedly lower when defining all patients with atrial fibrillation as ineligible (trial: 8% vs. 21%; pragmatic: 12% vs. 36%), as compared with the main analysis. The eligibility according to the complete-case analysis (trial: 20%; pragmatic: 32%) was slightly lower than the main analysis and slightly higher in the missing-as-eligible analysis (trial: 23%; pragmatic: 39%). Eligibility rates according to all criteria across sensitivity analyses are presented in S3 Table in the **S3 Appendix**.

### Patient characteristics

In both scenarios, eligible compared to ineligible patients in the overall cohort showed similar distribution for sex, but had more severe HF (i.e. had higher NYHA class and NT-proBNP, lower blood pressure, more likely used diuretics, and had more likely experienced a recent HF hospitalization), and lower income and education (**Table 1**). Eligible vs. ineligible patients in the trial scenario were comparable for age, were more likely to have diabetes, ischemic heart disease, atrial fibrillation, and pulmonary disease, but less likely to report liver disease and cancer, with no differences for stroke/TIA, major bleeding, hypertension, and previous coronary revascularization. Eligible patients in the pragmatic scenario were older and had higher burden of nearly all comorbidities, but had similar prevalence of cancer, hypertension, and previous coronary revascularization. Use of guideline-directed medical HF therapies (beta-blocker, RASi/ARNi, MRA, and sodium-glucose co-transporter 2 inhibitors [SGLT2i]) was higher in eligible vs. ineligible patients in the trial scenario. In the pragmatic scenario, beta-blocker and RASi/ARNi use were lower but MRA and SGLT2i inhibitor use higher in eligible vs. ineligible patients. Differences according to eligibility status were overall consistent with the overall cohort when assessed in the five sub-cohorts (EF<30%, in-patients, out-patients, severe HF, NYHA class III-IV, and NT-proBNP≥5,000pg/mL) (S4-S9 Tables in the **S1 Appendix**), and across sensitivity analyses considering deviating blood pressure as eligible (S10 Table in the **S1 Appendix**) and patients with atrial fibrillation with digoxin use as ineligible (S11 Table in the **S1 Appendix**). When considering a strict interpretation of the OMT criterion in the trial scenario (S12 Table in the **S1 Appendix**), and in both scenarios considering all patients with atrial fibrillation as ineligible (S13 Table in the **S1 Appendix**), eligible patients were overall younger, and had fewer comorbidities and lower NT-proBNP, as compared to eligible patients in the main analysis.

### Outcomes according to eligibility for OM

**Fig 2:**
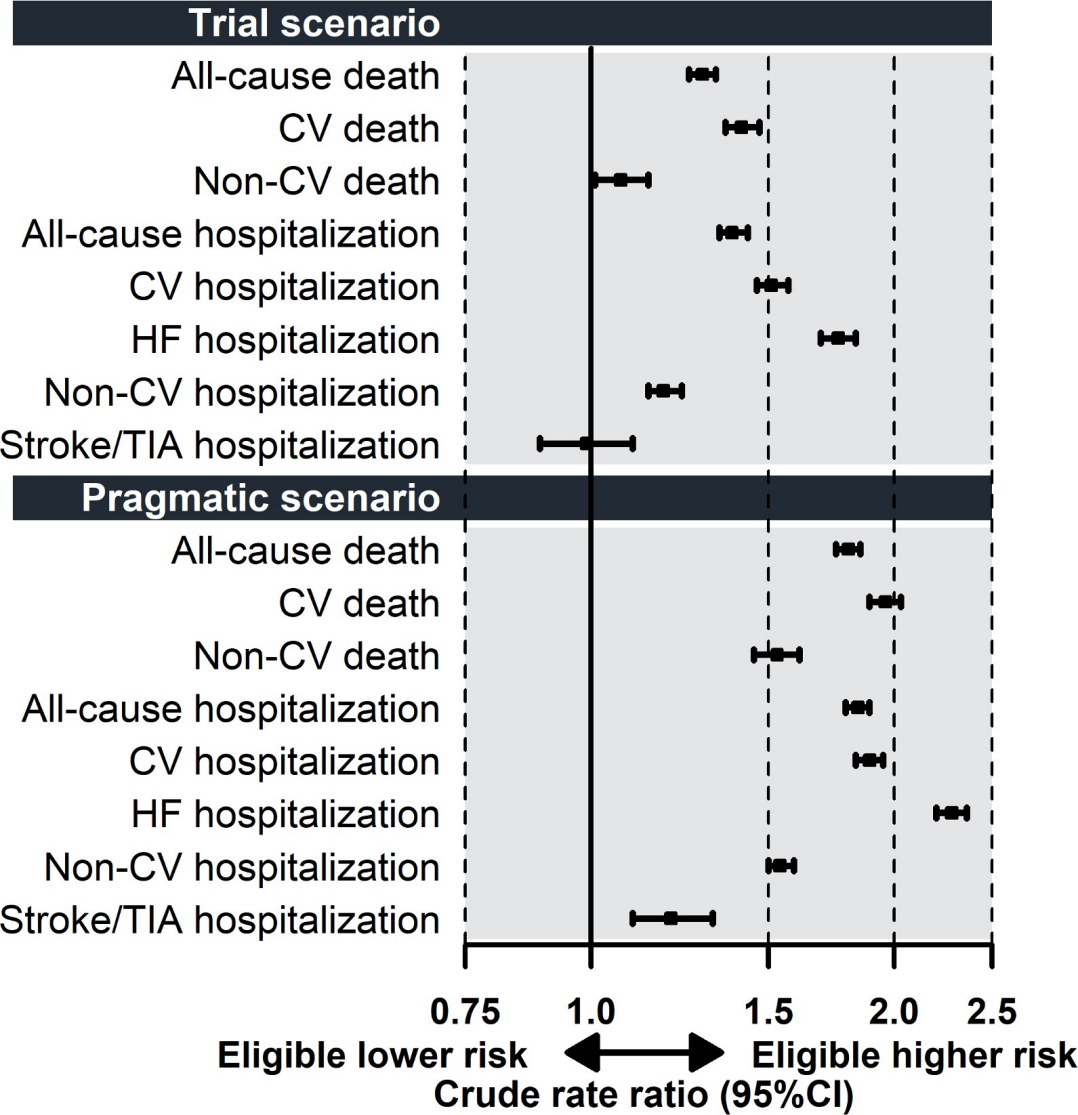
Incidence rate ratios in eligible and ineligible patients with ejection fraction <40% in the trial and pragmatic scenarios. **Abbreviations:** CI, confidence interval; CV, cardiovascular; HF, heart failure; TIA, transient ischemic attack.

In the overall (EF<40%) cohort, eligible patients according to both scenarios had higher absolute rates for all outcomes, except for stroke/TIA hospitalization whose rates did not differ according to eligibility in the trial scenario (**Fig 2**; **Table 3**). The IRRs of eligible vs ineligible patients were overall higher in the pragmatic scenario than in the trial scenario, and also overall greater for CV outcomes than for non-CV outcomes (e.g. CV death: IRR 1.41, 95% CI 1.36–1.47 [trial scenario] and 1.96, 95% CI 1.89–2.03 [pragmatic scenario]; non-CV death IRR 1.07, 95% CI 1.01–1.14 [trial scenario] and 1.53, 95% CI 1.45–1.61 [pragmatic scenario]).

**Table 3:** Comparison of event rates in eligible and ineligible patients in the trial and pragmatic scenario in patients with ejection fraction <40%.

	Events per 100 patient-years (95% CI)				
Event	Ineligible	Eligible		*IRR*	*P*
**Trial scenario**	** **	** **		** **	** **
All-cause death	18.5 (18.2–18.8)	23.8 (23.1–24.5)		1.29 (1.25–1.33)	<0.001
CV death	11.8 (11.6–12.1)	16.7 (16.1–17.3)		1.41 (1.36–1.47)	<0.001
Non-CV death	6.6 (6.5–6.8)	7.1 (6.7–7.5)		1.07 (1.01–1.14)	0.021
All-cause hospitalization	52.5 (51.7–53.3)	72.5 (70.6–74.5)		1.38 (1.34–1.43)	<0.001
CV hospitalization	25.9 (25.4–26.3)	39.2 (38.0–40.4)		1.51 (1.46–1.57)	<0.001
HF hospitalization	14.7 (14.4–15.1)	25.9 (25.0–26.8)		1.76 (1.69–1.83)	<0.001
Non-CV hospitalization	29.8 (29.2–30.3)	35.2 (34.1–36.4)		1.18 (1.14–1.23)	<0.001
Stroke/TIA hospitalization	2.3 (2.2–2.5)	2.3 (2.1–2.6)		0.99 (0.89–1.10)	0.914
**Pragmatic scenario**	** **	** **		** **	** **
All-cause death	15.7 (15.4–16.0)	28.2 (27.6–28.8)		1.80 (1.75–1.85)	<0.001
CV death	9.9 (9.7–10.1)	19.4 (18.9–19.9)		1.96 (1.89–2.03)	<0.001
Non-CV death	5.8 (5.6–6.0)	8.8 (8.5–9.2)		1.53 (1.45–1.61)	<0.001
All-cause hospitalization	46.0 (45.3–46.8)	84.7 (82.9–86.4)		1.84 (1.79–1.89)	<0.001
CV hospitalization	22.7 (22.3–23.2)	43.0 (42.0–44.0)		1.89 (1.83–1.95)	<0.001
HF hospitalization	12.4 (12.1–12.7)	28.3 (27.6–29.1)		2.28 (2.20–2.36)	<0.001
Non-CV hospitalization	26.7 (26.2–27.2)	41.2 (40.2–42.2)		1.54 (1.50–1.59)	<0.001
Stroke/TIA hospitalization	2.2 (2.1–2.3)	2.7 (2.5–2.8)		1.20 (1.10–1.32)	<0.001
**Trial scenario, OMT sensitivity**		** **		** **	** **
All-cause death	19.6 (19.3–19.9)	19.6 (18.7–20.5)		1.00 (0.95–1.05)	0.922
CV death	12.8 (12.5–13.0)	13.5 (12.8–14.3)		1.06 (1.00–1.12)	0.047
Non-CV death	6.8 (6.6–7.0)	6.1 (5.6–6.6)		0.89 (0.82–0.97)	0.01
All-cause hospitalization	55.3 (54.6–56.1)	62.6 (60.1–65.2)		1.13 (1.08–1.18)	<0.001
CV hospitalization	27.5 (27.0–27.9)	36.6 (34.9–38.3)		1.33 (1.27–1.40)	<0.001
HF hospitalization	16.0 (15.7–16.3)	25.1 (23.9–26.4)		1.57 (1.49–1.66)	<0.001
Non-CV hospitalization	30.9 (30.4–31.3)	30.4 (28.9–31.9)		0.98 (0.93–1.04)	0.545
Stroke/TIA hospitalization	2.4 (2.3–2.5)	2.0 (1.7–2.3)		0.85 (0.73–0.99)	0.036
**Trial scenario, BP sensitivity**		** **		** **	** **
All-cause death	18.3 (18.0–18.6)	24.3 (23.6–25.0)		1.33 (1.28–1.37)	<0.001
CV death	11.7 (11.5–11.9)	17.1 (16.5–17.7)		1.46 (1.41–1.52)	<0.001
Non-CV death	6.6 (6.4–6.8)	7.2 (6.8–7.6)		1.09 (1.02–1.15)	0.006
All-cause hospitalization	52.1 (51.4–52.9)	73.8 (71.8–75.8)		1.42 (1.37–1.46)	<0.001
CV hospitalization	25.7 (25.2–26.1)	39.9 (38.7–41.1)		1.55 (1.50–1.61)	<0.001
HF hospitalization	14.6 (14.3–14.9)	26.5 (25.6–27.4)		1.82 (1.75–1.89)	<0.001
Non-CV hospitalization	29.7 (29.2–30.2)	35.5 (34.4–36.6)		1.20 (1.15–1.24)	<0.001
Stroke/TIA hospitalization	2.3 (2.2–2.5)	2.3 (2.1–2.6)		1.00 (0.90–1.11)	0.979
**Trial scenario, AF+digoxin sensitivity**		** **		** **	** **
All-cause death	18.8 (18.5–19.1)	23.3 (22.6–24.1)		1.24 (1.20–1.29)	<0.001
CV death	12.1 (11.9–12.4)	16.2 (15.6–16.9)		1.34 (1.28–1.40)	<0.001
Non-CV death	6.7 (6.5–6.8)	7.1 (6.7–7.5)		1.06 (1.00–1.13)	0.056
All-cause hospitalization	53.4 (52.6–54.2)	70.8 (68.7–72.9)		1.33 (1.28–1.37)	<0.001
CV hospitalization	26.4 (26.0–26.9)	38.4 (37.2–39.8)		1.45 (1.40–1.51)	<0.001
HF hospitalization	15.2 (14.9–15.5)	25.4 (24.5–26.4)		1.67 (1.60–1.75)	<0.001
Non-CV hospitalization	30.0 (29.5–30.5)	34.9 (33.6–36.1)		1.16 (1.12–1.21)	<0.001
Stroke/TIA hospitalization	2.4 (2.3–2.5)	2.2 (2.0–2.5)		0.95 (0.84–1.07)	0.403
**Trial scenario, AF sensitivity**		** **		** **	** **
All-cause death	19.7 (19.4–20.0)	18.1 (17.2–19.0)		0.92 (0.87–0.97)	0.001
CV death	12.9 (12.6–13.1)	12.5 (11.8–13.3)		0.97 (0.91–1.04)	0.418
Non-CV death	6.8 (6.7–7.0)	5.6 (5.1–6.1)		0.82 (0.74–0.90)	<0.001
All-cause hospitalization	56.3 (55.5–57.1)	52.9 (50.5–55.4)		0.94 (0.90–0.99)	0.011
CV hospitalization	28.2 (27.7–28.6)	29.1 (27.6–30.6)		1.03 (0.98–1.09)	0.258
HF hospitalization	16.5 (16.2–16.9)	19.2 (18.1–20.3)		1.16 (1.09–1.23)	<0.001
Non-CV hospitalization	31.1 (30.7–31.6)	27.2 (25.8–28.7)		0.87 (0.83–0.92)	<0.001
Stroke/TIA hospitalization	2.4 (2.3–2.5)	1.9 (1.6–2.2)		0.78 (0.65–0.92)	0.002
**Pragmatic scenario, BP sensitivity**		** **		** **	** **
All-cause death	15.4 (15.1–15.7)	28.6 (28.0–29.3)		1.86 (1.81–1.92)	<0.001
CV death	9.7 (9.4–9.9)	19.7 (19.2–20.2)		2.04 (1.97–2.11)	<0.001
Non-CV death	5.7 (5.5–5.9)	8.9 (8.6–9.3)		1.56 (1.49–1.64)	<0.001
All-cause hospitalization	45.3 (44.6–46.1)	85.9 (84.2–87.7)		1.90 (1.85–1.95)	<0.001
CV hospitalization	22.3 (21.9–22.8)	43.6 (42.6–44.7)		1.95 (1.89–2.01)	<0.001
HF hospitalization	12.1 (11.8–12.4)	28.8 (28.1–29.6)		2.38 (2.29–2.47)	<0.001
Non-CV hospitalization	26.5 (26.0–27.0)	41.6 (40.6–42.6)		1.57 (1.52–1.62)	<0.001
Stroke/TIA hospitalization	2.2 (2.1–2.3)	2.7 (2.5–2.9)		1.21 (1.11–1.33)	<0.001
**Pragmatic scenario, AF+digoxin sensitivity**		** **		** **	** **
All-cause death	16.6 (16.3–16.9)	28.0 (27.4–28.7)		1.69 (1.64–1.74)	<0.001
CV death	10.6 (10.4–10.9)	19.1 (18.6–19.7)		1.80 (1.74–1.87)	<0.001
Non-CV death	6.0 (5.8–6.2)	8.9 (8.5–9.3)		1.49 (1.41–1.57)	<0.001
All-cause hospitalization	48.2 (47.4–49.0)	83.8 (81.9–85.7)		1.74 (1.69–1.79)	<0.001
CV hospitalization	23.9 (23.5–24.4)	42.6 (41.6–43.8)		1.78 (1.72–1.84)	<0.001
HF hospitalization	13.4 (13.1–13.7)	28.1 (27.3–28.9)		2.10 (2.02–2.18)	<0.001
Non-CV hospitalization	27.6 (27.1–28.1)	41.1 (40.0–42.2)		1.49 (1.44–1.54)	<0.001
Stroke/TIA hospitalization	2.3 (2.1–2.4)	2.6 (2.4–2.8)		1.14 (1.03–1.25)	0.008
**Pragmatic scenario, AF sensitivity**		** **		** **	** **
All-cause death	19.2 (18.9–19.5)	21.7 (20.8–22.5)		1.13 (1.08–1.17)	<0.001
CV death	12.6 (12.3–12.8)	14.4 (13.8–15.1)		1.15 (1.09–1.21)	<0.001
Non-CV death	6.7 (6.5–6.8)	7.2 (6.8–7.7)		1.08 (1.01–1.16)	0.028
All-cause hospitalization	55.0 (54.3–55.8)	63.1 (60.9–65.4)		1.15 (1.10–1.19)	<0.001
CV hospitalization	27.7 (27.3–28.2)	32.0 (30.7–33.3)		1.15 (1.10–1.20)	<0.001
HF hospitalization	16.2 (15.9–16.5)	21.1 (20.1–22.1)		1.30 (1.24–1.37)	<0.001
Non-CV hospitalization	30.6 (30.1–31.1)	32.2 (30.9–33.6)		1.05 (1.01–1.10)	0.022
Stroke/TIA hospitalization	2.4 (2.3–2.5)	2.0 (1.8–2.3)		0.84 (0.73–0.96)	0.01

**Abbreviations:** AF, atrial fibrillation; BP, blood pressure; CI, confidence interval; CV, cardiovascular; HF, heart failure; IRR, incidence rate ratio; OMT, optimal medical therapy; TIA, transient ischemic attack

As compared with the overall cohort, absolute event-rates were overall higher, and IRRs were overall lower (i.e. less difference between eligible vs. ineligible patients), in the EF<30%, in-patient, severe HF, NYHA III-IV, and NT-proBNP≥5,000pg/mL cohorts, whereas event-rates were overall lower and IRRs consistent in the out-patient cohort (S14-19 Tables in the **S1 Appendix**). In the EF<30% cohort, IRRs were directionally consistent with the overall EF<40% cohort, except that they were non-significant for non-CV death in the trial scenario, and stroke/TIA hospitalization in both scenarios (S14 Table in the **S1 Appendix**). In the in-patient cohort, eligible vs. ineligible patients in the trial scenario had higher risk only of CV and HF hospitalization, no difference in all-cause hospitalization, and lower risk of all-cause, CV, and non-CV death, non-CV hospitalization, and stroke/TIA hospitalization (S15 Table in the **S1 Appendix**). In severe HF, trial eligibility was associated with higher risk of all-cause, CV, and HF hospitalization, lower risk of non-CV death, and no difference in other outcomes (S17 Table in the **S1 Appendix**). In both the in-patient and severe HF cohorts, pragmatic eligibility was associated with higher risk of all outcomes, except for non-CV death and stroke/TIA hospitalization where there was no difference according to eligibility.

In the overall cohort (**Table 3**), the OMT sensitivity analysis (where the OMT criterion of the trial scenario was interpreted strictly) reported overall less pronounced differences in outcomes according to trial eligibility (i.e. lower IRRs), with higher risk in eligible patients only for CV death, and all-cause, CV and HF hospitalization, no significant difference in all-cause death or non-CV hospitalizations, and lower risk in eligible patients for non-CV death and stroke/TIA hospitalization. In the sensitivity analyses where hypotension did not lead to exclusion, and where atrial fibrillation with digoxin use led to exclusion, IRRs in both scenarios were overall consistent with the main analysis. In the sensitivity analysis classifying all patients with atrial fibrillation as ineligible, eligible patients in the trial scenario had higher risk only of HF hospitalization, similar risk of CV death and CV hospitalization, and lower risk of all-cause and non-CV death, all-cause, non-CV, and stroke/TIA hospitalization, whereas eligible patients in the pragmatic scenario had lower rates of stroke/TIA hospitalization and higher risk of all other outcomes.

The complete case and missing-as-eligible analyses showed findings largely consistent with the main analysis (S20, S21 Tables in the **S1 Appendix**).

## Discussion

In this comprehensive characterization of eligibility for OM in a large real-world chronic HFrEF cohort, we observed that i) 21% of patients were eligible according to the GALACTIC-HF criteria (trial scenario), and 36% according to criteria more likely to impact use in clinical practice (pragmatic scenario); ii) the criteria that limited eligibility the most across scenarios was the requirement for a recent event of worsening HF (met by 60% of patients); iii) eligibility was higher with EF<30% (trial: 27%; pragmatic: 44%), in-patient status, (trial: 30%; pragmatic: 57%) and severe HF (trial: 35%; pragmatic: 60%); iv) eligible patients in both scenarios were characterized by more severe HF, and in the pragmatic scenario by higher comorbidity burden; v) eligible patients had higher crude risk of CV outcomes in the overall HFrEF cohort and in patients with EF<30%, but this difference was lesser among in-patients and patients with severe HF. Although this specific analysis is based on GALACTIC-HF and OM, our findings might inform multiple stakeholders on the consequences in terms of patient eligibility when adopting specific selection criteria in trials and labeling criteria for HFrEF and more specifically severe HFrEF treatments.

In the overall patient cohort of chronic HFrEF, we estimated 21% eligibility for OM according to the trial scenario and 36% according to the pragmatic scenario. The trial eligibility was lower than for other HFrEF treatments in SwedeHF, including ARNi (38%) [14], dapagliflozin (35%) and empagliflozin (31%) [20], but higher than ivabradine (14%) [21]. The criteria that most limited OM eligibility in both scenarios were the requirement of an episode of worsening HF in the past 12 months (met by 60% in the overall cohort) and elevated natriuretic peptides (met by 82%). SwedeHF has been used to estimate real-world eligibility for two previous trials focusing on worsening HF: SOLOIST-WHF and VICTORIA [13, 22]. The wider timeframe for recent worsening HF event in GALACTIC-HF (12 months vs. 6 months in VICTORIA) contributed to more patients meeting this criterion (60%) than in VICTORIA (44%). However, the wider timeframe also likely contributed to the overall lower IRRs for eligible vs. ineligible patients observed in this study (e.g. 41% and 70% higher event-rates for CV death in patients who were eligible vs. ineligible for GALACTIC-HF and VICTORIA, respectively). This highlights that for trials focusing on worsening HF, a wider timeframe to define a recent worsening HF event might improve eligibility, but also compromise the enrichment for CV events since the risk of events is likely highest in the early vulnerable period of the first 2–3 months post-discharge [23].

One previous study utilized electronic health records and administrative claims data to estimate OM eligibility according to GALACTIC-HF criteria in a Californian HFrEF cohort, reporting an eligibility of 37% [24]. Our considerably lower estimate for the trial scenario (21%) might be due to the application of a larger number of the exclusion criteria of GALACTIC-HF, enabled by the use of our well-characterized HF registry. In 455 patients hospitalized for acute HFrEF in the Canadian HF (CAN-HF) registry, eligibility according to GALACTIC-HF enrolment criteria was estimated at 30% [25], which is again higher than our estimates in chronic HFrEF (21%) but considerably lower than the ones for in-patients (57%). This discrepancy is likely explained by their use of a strict interpretation of the OMT criterion (i.e. patients were required to receive RASi/ARNI+betablocker+MRA to be eligible), which was unmet by 30% of patients in CAN-HF [25]. We performed a trial scenario sensitivity analysis with a similarly strict interpretation of the OMT criterion, which was met only by 42% of our cohort and yielded an overall trial eligibility of only 10%. The primary reason for not meeting the literal interpretation of OMT was the underuse of MRA in approximately ½ of the population. This highlights the difficulty of achieving OMT in clinical practice [2, 3, 26], where poor tolerance and/or contraindications to neurohormonal therapies are common. The distinct mechanism of OM suggests that its efficacy is independent of background OMT and that OMT should not be a prerequisite for initiation of OM. Since OM does not impair blood pressure, renal clearance, or serum potassium [10, 27], it might offer the opportunity to treat patients reporting these barriers to the implementation of other HFrEF therapies. Moreover, in severe HF where patients are often less prone to tolerate hypotensive effects of guideline-directed medical therapies, myotropes such as OM might facilitate initiation and/or continuation. Although the overall relative reduction in risk of HF events or CV death was modest in GALACTIC-HF [28], and non-significant according to a meta-analysis [29], subgroup and post-hoc analyses from GALACTIC-HF have suggested a greater benefit with OM in patients with lower EF and greater HF severity [30, 31]. In our analysis, these patients were also considerably more likely eligible for OM according to the trial as well as pragmatic criteria (EF<30%: 27% trial and 44% pragmatic eligibility; severe HF: 35% trial and 60% pragmatic eligibility).

When comparing the patients who were enrolled in GALACTIC-HF with real-world patients who according to our analysis would have been eligible for enrolment, some important differences emerge (S22 Table in the **S1 Appendix**). Eligible patients in SwedeHF, compared to the GALACTIC-HF study population, were older (mean age 74 vs. 65 years), more likely female (27% vs. 21%), more likely in NYHA classes III-IV (63% vs. 47%), had higher NT-proBNP levels (median 4,250 vs. 1,971 pg/mL), and with higher prevalence of atrial fibrillation (63% vs. 42%) and ischemic heart disease (69% vs. 62%), but lower prevalence of hypertension (65% vs. 70%) [10]. The higher prevalence of atrial fibrillation among eligible real-world patients vs. GALACTIC-HF is noteworthy, since post-hoc analyses of GALACTIC-HF suggested that OM was potentially linked with harm in patients with atrial fibrillation treated with digoxin [10, 18]. Concomitant atrial fibrillation and digoxin were present in 16% of eligible patients in the trial scenario in our registry, vs. 8% in GALACTIC-HF. Importantly, when compared to the placebo arm of GALACTIC-HF, eligible patients in SwedeHF also had markedly higher event-rates of outcomes, e.g. CV death (17 vs. 11 events per 100 patient-years), all-cause death (24 vs. 14 events per 100 patient-years), and first HF hospitalization (26 vs. 19 events per 100 patient-years) [10]. This might lead to speculation that the absolute risk reduction with OM might be greater in the real-world vs. the GALACTIC-HF setting. Some of these differences might result from regional variations, as well as from a high degree of selection being introduced already when investigators choose patients to screen for trial enrolment. Indeed, approximately 75% of patients screened for GALACTIC-HF met the eligibility criteria [28], far exceeding the 21% eligibility observed in unselected patients with chronic HFrEF, further supporting this interpretation. Although the observed differences might raise questions regarding generalizability, they are not unique to GALACTIC-HF; similar patterns have been observed in previous studies assessing real-world eligibility for other HFrEF trials [32]. Taken together, these findings emphasize that trial generalizability goes beyond the design of eligibility criteria and requires a conscious and active effort at all sites engaged in RCT enrolment.

### Limitations

The well-characterized and large SwedeHF cohort enabled the detailed application of GALACTIC-HF selection criteria to a real-world HFrEF population of over 31,000 patients. However, there are limitations that should be considered when interpreting our findings. First, not all criteria could be precisely reproduced according to the obtained variables. This led to the adaption of proxies for few criteria. For some of the nonreproducible criteria, such as informed consent, childbearing potential, and OM oversensitivity, we opted to assume 100% eligibility. Second, we had missing data for certain variables that were required for eligibility calculations. This was addressed by sensitivity analyses where we applied alternative methods for handling missing data, and these yielded results that were largely consistent with the main analysis. Third, patients enrolled in SwedeHF had higher event rates than patients in GALACTIC, but have lower comorbidity burden and better outcomes than patients with HF in Sweden who are not enrolled in the registry [33]. Lastly, the potential real-world use of medications is affected by several factors not addressed in this study, including patient preferences, drug availability, reimbursements, and regulatory labels. According to a recent decision from the U.S. Food and Drug Administration, the evidence from GALACTIC-HF alone was not sufficient for approval of OM. The decision from the European Medicines Agency (EMA) is still awaited. Label indications are generally less detailed than trial criteria, and a potential EMA approval might imply greater eligibility than the pragmatic scenario applied in this study.

## Conclusion

In this comprehensive characterization of eligibility for OM in a large real-world HFrEF cohort, approximately 21% of patients were eligible according to the literal GALACTIC-HF trial criteria, and 36% according to the pragmatic criteria most likely to determine use in clinical practice. Eligibility for OM was considerably higher in patients with lower EF (27% trial and 44% pragmatic) and severe HF (35% trial and 60% pragmatic), i.e. those subgroups where GALACTIC-HF demonstrated the greatest benefit of OM. Eligibility according to either scenario translated to the selection of a population at high risk for CV and HF events.

## Supporting information

S1 Appendix Supplementary tables and figures. (DOCX)
