# Supplementary material for: Eligibility for omecamtiv mecarbil in a real-world heart failure population: Data from the Swedish Heart Failure Registry
Source: PLoS One. 2024 May 24;19(5):e0303348. doi: 10.1371/journal.pone.0303348 (PMC11125482; doi:10.1371/journal.pone.0303348)
Supplement: S1 Appendix — (DOCX) [file pone.0303348.s001.docx]

**SUPPLEMENTARY MATERIAL**

**Eligibility for omecamtiv mecarbil in a real-world heart failure population: data from the Swedish Heart Failure Registry**

**Authors:** Felix Lindberg^1*^, Natanael Øigaard^1*^, Marco Metra^2^, Giuseppe MC Rosano^3,4^, Ulf Dahlström^5^, Peter Mol^6^, Camilla Hage^1,7^, Lars H Lund^1,7^, Gianluigi Savarese^1,7^

* These authors equally contributed as first author

**Affiliations:**

1. Division of Cardiology, Department of Medicine, Karolinska Institutet, Stockholm, Sweden.

2. Cardiology, Department of Medical and Surgical Specialties, Radiological Sciences and Public Health, University of Brescia, Cardiothoracic Department, Civil Hospitals, Brescia, Italy

3. Cardiovascular Clinical Academic Group, Molecular and Clinical Research Institute, St George’s University Hospital, London, United Kingdom

4. Cardiology, San Raffaele Cassino Hospital, Cassino, Italy

5. Department of Cardiology and Department of Health, Medicine and Caring Sciences, Linköping University, Linköping, Sweden.

6. Department of Clinical Pharmacy and Pharmacology, University Medical Center Groningen, University of Groningen, Groningen, The Netherlands.

7. Heart and Vascular and Neuro Theme, Karolinska University Hospital, Stockholm, Sweden.

**Table of contents**

| **Item** | **Description** | **Page** |
| --- | --- | --- |
| **Table S1.** | Eligibility criteria of the GALACTIC-HF trial and corresponding adaptions and data sources. | **3** |
| **Table S2.** | Patient characteristics for patients with ejection fraction <40%, ejection fraction <30%, in-patients, out-patients, patients with severe heart failure, patients with NYHA class III-IV, and patients with NT-proBNP≥5,000pg/mL. | **14** |
| Table S3. | Eligibity for omecamtiv mecarbil according to all criteria in the trial and pragmatic scenarios in sensitivity analyses for missing data. | **18** |
| Table S4 | Baseline characteristics according to eligibility for omecamtiv mecarbil (trial and pragmatic scenarios) in patients with ejection fraction <30%. | **20** |
| Table S5 | Baseline characteristics according to eligibility for omecamtiv mecarbil (trial and pragmatic scenarios) in in-patients. | **24** |
| Table S6 | Baseline characteristics according to eligibility for omecamtiv mecarbil (trial and pragmatic scenarios) in out-patients. | **28** |
| Table S7 | Baseline characteristics according to eligibility for omecamtiv mecarbil (trial and pragmatic scenarios) in patients with severe heart failure. | **32** |
| Table S8 | Baseline characteristics according to eligibility for omecamtiv mecarbil (trial and pragmatic scenarios) in patients with NYHA class III-IV. | **36** |
| Table S9 | Baseline characteristics according to eligibility for omecamtiv mecarbil (trial and pragmatic scenarios) in patients with NT-proBNP≥5,000pg/mL. | **40** |
| Table S10 | Baseline characteristics according to eligibility for omecamtiv mecarbil (trial and pragmatic scenario sensitivity analysis considering as eligible also patients with hypotension). | **44** |
| Table S11 | Baseline characteristics according to eligibility for omecamtiv mecarbil (trial and pragmatic scenario sensitivity analyses considering as ineligible all patients with atrial fibrillation and digoxin use). | **48** |
| Table S12 | Baseline characteristics according to eligibility for omecamtiv mecarbil (trial scenario sensitivity analysis considering a strict interpretation of the optimal-medical-therapy criterion). | **52** |
| Table S13 | Baseline characteristics according to eligibility for omecamtiv mecarbil (trial and pragmatic scenario sensitivity analyses considering as ineligible all patients with atrial fibrillation). | **54** |
| Table S14 | Outcomes in eligible and ineligible patients in the trial and pragmatic scenario in patients with ejection fraction <30%. | **58** |
| **Table S15** | Outcomes in eligible and ineligible patients in the trial and pragmatic scenario in in-patients. | **60** |
| **Table S16** | Outcomes in eligible and ineligible patients in the trial and pragmatic scenario in out-patients. | **62** |
| **Table S17** | Outcomes in eligible and ineligible patients in the trial and pragmatic scenario in patients with severe heart failure. | **64** |
| **Table S18** | Outcomes in eligible and ineligible patients in the trial and pragmatic scenario in patients with NYHA class III-IV. | **66** |
| **Table S19** | Outcomes in eligible and ineligible patients in the trial and pragmatic scenario in patients with NT-proBNP≥5,000pg/mL. | **68** |
| **Table S20** | Outcomes in eligible and ineligible patients in the trial and pragmatic scenario in the complete case analysis. | **70** |
| **Table S21** | Outcomes in eligible and ineligible patients in the trial and pragmatic scenario in the missing-as-eligible analysis. | **71** |
| **Table S22** | Comparison of patient characteristics in patients eligible for GALACTIC-HF in SwedeHF, vs. the GALACTIC-HF trial population | **72** |
| **Figure S1** | Patient selection flowchart. | **73** |

**Table S1. Eligibility criteria of the GALACTIC-HF trial and corresponding adaptions and data sources used in current study.**

| **Eligibility criterion** | **Adapted criterion** | **Data sources and codes** |
| --- | --- | --- |
| **Inclusion** |  |  |
| 1. Informed consent | Assumed |  |
| 2. Male or female, ≥ 18 to ≤ 85 years | Male or female, ≥ 18 to ≤ 85 years | *Data source:  SwedeHF*  *Variables:*  shf_age,  shf_sex |
| 3. Requiring treatment for HF for a minimum of 30 days before randomization | HF duration of ≥ 30 days | *Data source:* SwedeHF  *Variable:*  shf_duration_hf (calculated from NPR) |
| 4. Left ventricular EF ≤ 35% within 12 months prior to screening. The most recent qualifying left ventricular EF must be at least 30 days after any of the following, if applicable: | Main analysis: left ventricular EF < 40% Sensitivity analysis: left ventricular EF < 30% | *Data source*:  SwedeHF  *Variable*:  shf_ef |
| a) an event likely to decrease EF (e.g., myocardial infarction, sepsis); | One of the following events within 30 days prior to index date:  a) Acute myocarditis  b) Acute myocardial infarction  c) Subsequent myocardial infarction  d) Salmonella sepsis  e) Listerial sepsis  f) Acute meningococcaemia  g) Streptococcal sepsis  h) Other sepsis  i) systemic inflammatory response and sepsis according to SEPSIS-3 | *Data source*:  NPR  *ICD-10 codes:* I21, I22, I40, I41, I51.4, A02.1, A32.7, A39.2, A40, A41, A37.7, R65 I21 Acute myocardial infarction I22 Subsequent ST elevation (STEMI) and non-ST elevation (NSTEMI) myocardial infarction  I40 Acute myocarditis I41 Myocarditis in diseases classified elsewhere I51.4 Myocarditis, unspecified A02.1 Salmonella sepsis A32.7 Listerial sepsis A39.2 Acute meningococcemia A40 Streptococcal sepsis A41 Other sepsis B37.7 Candidal sepsis R65 Symptoms and signs specifically associated with systemic inflammation and infection |
| b) an intervention likely to increase EF (e.g., cardiac resynchronization therapy, coronary revascularization); | One of the following events within 30 days prior to index date:  a) CRT implantation  b) CRT-D implantation  c) PCI  d) CABG | *Data source:* NPR  *KVÅ-codes:* FPE26, FPG36, FNA-FNH FPE26 Insertion of transvenous pacemaker with biventricular electrodes FPG36 Insertion of transvenous cardioverter-defibrillator with biventricular electrodes  FNA Anastomosis between internal thoracic artery and coronary arteries  FNB Anastomosis between gastroepiploic arteries and coronary arteries  FNC Aortocoronary venous bypass FND Aortocoronary bypass with vascular prosthesis  FNE Coronary bypass with free arterial grafts  FNF Coronary thrombectomy  FNG Dilation and recanalization of coronary arteries  FNH Reconstruction of coronary artery |
| c) the first ever presentation for HF | HF duration < 30 days | *Data source:* SwedeHF  *Variable:* shf_durationhf (calculated from NPR) |
| 5. NYHA class II to IV | NYHA class II to IV | *Data source:*  SwedeHF  *Variable:* shf_nyha |
| 6. Managed with HF standard of care therapies consistent with regional clinical practice guidelines. Oral standard of care therapies for chronic HF (eg, beta blockers, renin-angiotensin-aldosterone system inhibitors) should be present, if not contraindicated | ≥ 3 months since HF diagnosis (thereby assuming sufficient time to achieve optimal care)  Sensitivity analysis: concomitant use of beta-blocker, RAAS-inhibitor/ARNi and MRA at baseline | *Data source:* SwedeHF  *Variables*:  shf_durationhf shf_betablocker shf_rasi  shf_arni  shf_mra |
| 7. Currently hospitalized with primary reason of HF OR one of the following events within 1 year to screening:  1) hospitalization with primary reason of HF; 2) urgent visit to ED with primary reason of HF | Hospitalization due to HF ≤ 12 months prior to index date | *Data source:*  SwedeHF  *Variable:*  sos_prevhosphf sos_location |
| 8. B-type natriuretic peptide (BNP) level ≥ 125 pg/mL or an NT-proBNP level ≥ 400 pg/mL (for subjects in atrial fibrillation/flutter at screening, the cut off levels are: BNP ≥ 375 pg/mL or NT-proBNP ≥ 1200 pg/mL) | NT-proBNP level ≥ 400 pg/mL, ≥ 1200 pg/mL for atrial fibrillation/flutter | *Data source:*  SwedeHF  *Variables:* shf_ntprobnp  sos_com_af shf_af |
| **Exclusion** |  |  |
| 1. Currently receiving treatment in another investigational device or drug study, or < 30 days since ending treatment on another investigational device or drug study(ies) | Not included |  |
| 2. Malignancy within 5 years prior to randomization with the following exceptions: localized basal or squamous cell carcinoma of the skin, cervical intraepithelial neoplasia, stage 1 prostate carcinoma, breast ductal carcinoma in situ. | History of malignancies other than localized basal/squamous cell carcinoma within 5 years prior to index date | *Data source*:  NPR  *ICD-10 codes:*  C00-C97, D00-D09 Neoplasms *with the exception of:* C44.0B, C44.0C, C44.0D, C44.0E, C44.0S, C44.1B, C44.1C, C44.1D, C44.1E, C44.1S, C44.2B, C44.2C, C44.2D, C44.2E, C44.2S, C44.3B, C44.3C, C44.3D, C44.3E, C44.3S, C44.4B, C44.4C, C44.4D, C44.4E, C44.4S, C44.5B, C44.5C, C44.5D, C44.5E, C44.5S, C44.6B, C44.6C, C44.6D, C44.6E, C44.6S, C44.7B, C44.7C, C44.7D, C44.7E, C44.7S, C44.8B, C44.8C, C44.8D, C44.8E, C44.8S, C44.9B, C44.9C, C44.9D, C44.9E, C44.9S, D05.1, D06  C44.0B Superficial basal cell carcinoma of the skin of the lip C44.0C Morphea-like basal cell carcinoma of the skin of the lip  C440D Other basal cell carcinoma of the skin of the lip C44.0E Unspecified basal cell carcinoma of the skin of the lip C44.0S Squamous cell carcinoma of the skin of the lip  C44.1B Superficial basal cell carcinoma of the skin of the eyelid including the canthus C44.1C Morphea-like basal cell carcinoma of the skin of the eyelids including the canthus  C44.1D Other basal cell carcinoma of skin of eyelid including the canthus C44.1E Unspecified basal cell carcinoma of the skin of the eyelid including the canthus C44.1S Squamous cell carcinoma of the skin of the eyelid including the canthus C44.2B Superficial basal cell carcinoma of the skin of the outer ear and of the external auditory meatus  C44.2C Morphea-like basal cell carcinoma of the skin of the outer ear and of the external auditory meatus C44.2D Other basal cell carcinoma of the skin of the outer ear and of the external auditory meatus C44.2E Unspecified basal cell carcinoma of the skin of the outer ear and of the external auditory meatus  C44.2S Squamous cell carcinoma of the skin of the outer ear and of the external auditory meatus  C44.3B Superficially growing basal cell carcinoma of the skin of other and unspecified parts of the face  C44.3C Morphea-like basal cell carcinoma of the skin of other and unspecified parts of the face C44.3D Other basal cell carcinoma of the skin of other and unspecified parts of the face  C44.3E Unspecified basal cell carcinoma of the skin of other and unspecified parts of the face C44.3S Squamous cell carcinoma of the skin of other and unspecified parts of the face  C44.4B Superficial basal cell carcinoma of the skin of the scalp and neck  C44.4C Morphea-like basal cell carcinoma of skin of scalp and neck  C44.4D Other basal cell carcinoma of the skin, scalp and neck  C44.4E Unspecified basal cell carcinoma of the skin of the scalp and neck  C44.4S Squamous cell carcinoma of the skin of the scalp and neck  C44.5B Superficial basal cell carcinoma of the skin of the trunk  C44.5C Morphea-like basal cell carcinoma of the skin of the trunk  C44.5D Other basal cell carcinoma of the skin of the trunk C44.5E Unspecified basal cell carcinoma of the skin of the trunk  C44.5S Squamous cell carcinoma of the skin of the trunk C44.6B Superficial basal cell carcinoma of the skin of the upper extremity including the shoulder  C44.6C Morphea-like basal cell carcinoma of the skin of the upper extremity including the shoulder  C44.6D Other basal cell carcinoma of the skin of the upper limb including the shoulder  C44.6E Unspecified basal cell carcinoma of the skin of the upper extremity including the shoulder  C44.6S Squamous cell carcinoma of the skin of the upper extremity including the shoulder C44.7B Superficially growing basal cell carcinoma of the skin of the lower extremity including the hip  C44.7C Morphea-like basal cell carcinoma of the skin of the lower extremity including the hip  C44.7D Other basal cell carcinoma of the skin of the lower limb including the hip  C44.7E Unspecified basal cell carcinoma of the skin of the lower extremity including the hip  C44.7S Squamous cell carcinoma of the skin of the lower extremity including the hip C44.8B Superficially growing basal cell carcinoma of the skin with overlapping growth  C44.8C Morphea-like basal cell carcinoma of the skin with overlapping growth  C44.8D Other basal cell carcinoma of the skin with overlapping growth  C44.8E Unspecified basal cell carcinoma of the skin with overlapping growth  C44.8S Squamous cell carcinoma of the skin with overlapping growth  C44.9B Superficial basal cell carcinoma with unspecified localisation  C44.9C Superficial basal cell carcinoma with unspecified localisation  C44.9D Other basal cell carcinoma with unspecified localisation  C44.9E Unspecified basal cell carcinoma with unspecified localisation  C44.9S Squamous cell carcinoma of the skin with unspecified localisation  D05.1 Intraductal carcinoma in situ of breast D06 Carcinoma in situ of cervix uteri |
| 3. Subject has known sensitivity to any of the products or components to be administered during dosing | Not included |  |
| 4. Factors expected to interfere with the subject’s availability or ability to complete all protocol-required study visits or procedures, and/or to comply with all required study procedures to the best of the subject and investigator’s knowledge (including ongoing substance abuse). | History of one of the following:  a) dementia  b) organic amnesic syndrome  c) delirium  d) other mental disorders due to brain damage and dysfunction and to physical disease  e) personality and behavioural disorders due to brain disease, damage and dysfunction  f) unspecified organic or symptomatic mental disorder  g) mental and behavioural disorders due to use of alcohol, opioids, cannabinoids, sedatives, cocaine, hallucinogens, volatile solvents or other stimulants including caffeine  h) mental and behavioural disorders due to multiple drug use and use of other psychoactive substances  i) schizophrenia, schizotypal disorder, persistent delusional disorders, acute and transient psychotic disorders, induced delusional disorder and schizoaffective disorders  j) other nonorganic psychotic disorders  k) unspecified nonorganic psychosis  l) hypomania  m) bipolar affective disorder  n) Pellagra (alcoholic)  o) degeneration of nervous system due to alcohol  p) alcoholic polyneuropathy, myopathy, cardiomyopathy or gastritis  q) alcoholic liver disease  r) toxic effect of alcohol  s) alcohol rehabilitation  t) alcohol abuse counselling and surveillance | *Data source*:  NPR  *ICD-10 codes*: F00-F07, F09-F16, F18-F25, F28-F31, E52, G31.2, G62.1, G72.1, I42.6, K29.2, K70, T51, Z71.4 F00 Dementia in Alzheimer disease F01 Vascular dementia F02 Dementia in other diseases classified elsewhere F03 Unspecified dementia F04 Amnestic disorder due to known physiological condition F05 Delirium due to known physiological condition F06 Other mental disorders due to known physiological condition F07 Personality and behavioural disorders due to known physiological condiion F09 Unspecified mental disorder due to known physiological condition F10 Alcohol related disorders F11 Opioid related disorders F12 Cannabis related disorders F13 Sedative, hypnotic, or anxiolytic related disorders F14 Cocaine related disorders F15 Other stimulant related disorders F16 Hallucinogen related disorders F18 Inhalant related disorders F19 Other psychoactive substance related disorders F20 Schizophrenia F21 Schizotypal disorder F22 Delusional disorders F23 Brief psychotic disorder F24 Shared psychotic disorder F25 Schizoaffective disorders  F28 Other psychotic disorder not due to a substance or known physiological condition F29 Unspecified psychosis not due to a substance or known physiological condition F30 Manic episode F31 Bipolar disorder E52 Niacin deficiency (pellagra) G31.2 Degeneration of nervous system due to alcohol G62.1 Alcoholic polyneuropathy G72.1 Alcoholic myopathy I42.6 Alcoholic cardiomyopathy K29.2 Alcoholic gastritis K70 Alcoholic liver disease T51 Toxic effect of alcohol Z71.4. Alcohol abuse counselling and surveillance |
| 5. Inability to swallow study medication tablet (eg, swallowing disorders, feeding tubes) | Not included |  |
| 6. Receiving mechanical hemodynamic support (eg, intra-aortic balloon pump counterpulsation), or invasive mechanical ventilation ≤ 7 days prior to randomization | Not included |  |
| 7. Receiving IV inotropes (eg, dobutamine, milrinone, levosimendan) or IV vasopressors (eg, epinephrine, norepinephrine, dopamine, or vasopressin) ≤ 3 days prior to randomization | Not included |  |
| 8. Receiving IV diuretics or IV vasodilators, supplemental oxygen therapy, or non-invasive mechanical ventilation (eg, bilevel positive airway pressure [BiPAP] or continuous positive airway pressure [CPAP] ≤ 12 hours prior to randomization | Not included |  |
| 9. Acute coronary syndrome (ST-elevation myocardial infarction, non-ST-elevation myocardial infarction, unstable angina), stroke, or transient ischaemic attack, major cardiac surgery or cardiac intervention (i.e., implantation of cardiac closure devices, cardiac resynchronization therapy, or catheter ablation), percutaneous coronary intervention, or valvuloplasty/other cardiac valve repair or implantation within the 3 months prior to randomization | Any of the following ≤ 3 months prior to index date:  a) Unstable angina  b) Myocardial infarction or reinfarction  c) Subarachnoid or intracerebral hemorrhage  d) Other nontraumatic intracranial hemorrhage  e) Cerebral infarction  f) Stroke, not specified as haemorrhage or infarction  g) Occlusion and stenosis of carotid artery  h) Surgery on heart or large intrathoracic vessels | *Data source:*  NPR  *ICD-10 codes:* I20.0, I21, I22, I60-I64, I65.2, G45 I20.0 Unstable angina I21 Acute myocardial infarction I22 Subsequent ST elevation (STEMI) and non-ST elevation (NSTEMI) myocardial infarction I60 Nontraumatic subarachnoid hemorrhage I61 Nontraumatic intracerebral hemorrhage  I62 Other and unspecified nontraumatic intracranial hemorrhage  I63 Cerebral infarction I64 Stroke, not specified as haemorrhage or infarction I65.2 Occlusion and stenosis of carotid artery G45 Transient cerebral ischemic attacks and related syndromes   *KVÅ-codes:* F  F Operations on the heart and large intrathoracic vessels |
| 10. Insertion of other cardiac devices (eg, implantable cardioverter defibrillator, permanent pacemaker, monitoring devices) within 30 days prior to randomization | One of the following procedures ≤ 30 days prior to index date:  a) Implant or change of permanent transveneous pacemaker  b) Implant or change of permanent epicardial pacemaker  c) Implant of ICD | *Data source:* NPR  *KVÅ-codes:* FPE-FPG FPE Insertion or replacement of permanent transvenous pacemaker  FPF Insertion or replacement of permanent epicardial pacemaker  FPG Insertion of permanent cardioverter-defibrillator |
| 11. Severe uncorrected valvular heart disease, hypertrophic or infiltrative cardiomyopathy, active myocarditis, constrictive pericarditis, or clinically significant congenital heart disease | Congenital heart disease, eosinophilic cardiomyopathy, other restrictive cardiomyopathy, other/unknown cardiomyopathy, active myocarditis, constrictive pericarditis, hypertrophic cardiomyopathy. | *Data source:*  NPR  *ICD-10-codes:* Q20-Q28, I01.2, I31.0, I31.1, I40.0, I40.1, I40.8, I40.9, I41.0-I41.2, I42.1-I41.3, I42.5, I42.8, I43.1, I51.4  Q20 Congenital malformation of cardiac chambers and connections  Q21 Congenital malformations of cardiac septa  Q22 Congenital malformations of pulmonary and tricuspid valves  Q23 Congenital malformations of aortic and mitral valves  Q24 Other congenital malformations of heart  Q25 Congenital malformations of great arteries  Q26 Congenital malformations of great veins  Q27 Other congenital malformations of peripheral vascular system  Q28 Other congenital malformations of circulatory system  I01.2 Acute rheumatic myocarditis  I31.0 Chronic adhesive pericarditis I31.1 Chronic constrictive pericarditis  I40.0 Infective myocarditis  I40.1 Isolated myocarditis I40.8 Other acute myocarditis I40.9 Acute myocarditis, unspecified  I41.0 Myocarditis in bacterial diseases classified elsewhere I41.1 Myocarditis in viral diseases classified elsewhere I41.2 Myocarditis in other infectious and parasitic diseases classified elsewhere I42.1 Obstructive hypertrophic cardiomyopathy I42.2 Other hypertrophic cardiomyopathy I42.3 Endomyocardial (eosinophilic) disease I42.5 Other restrictive cardiomyopathy I42.8 Other cardiomyopathies  I43.1 Cardiomyopathy with metabolic disease (incl. Danon) I51.4 Myocarditis, unspecified |
| 12. Untreated severe ventricular arrhythmia (eg, ventricular tachycardia or ventricular fibrillation) | Not included |  |
| 13. Chronic antiarrhythmic therapy, with the exception of amiodarone. Note: for the purposes of this exclusion criterion, digoxin, calcium channel blocker, and beta-blocker therapy are not considered to be chronic antiarrhythmic therapies | Not included |  |
| 14. Symptomatic bradycardia or second or third degree heart block without a pacemaker | Not included |  |
| 15. Routinely scheduled outpatient intravenous infusions for HF (eg, inotropes, vasodilators [eg, nesiritide], diuretics) or routinely scheduled ultrafiltration | Not included |  |
| 16. Systolic blood pressure > 140 mmHg or < 85 mmHg, or diastolic blood pressure > 90 mmHg, or heart rate > 110 beats per minute, or < 50 beats per minute at screening | Systolic blood pressure > 140 mmHg or < 85 mmHg, or diastolic blood pressure > 90 mmHg, or heart rate > 110 beats per minute, or < 50 beats per minute at index date  Sensitivity analysis: Systolic blood pressure <85mmHg does not lead to exclusion | *Data source:* SwedeHF  *Variables:* shf_bpsys shf_bpdia shf_heartrate |
| 17. Estimated glomerular filtration rate (eGFR) < 20 mL/min/1.73m2 or receiving dialysis at screening | Estimated glomerular filtration rate (eGFR) < 20 mL/min/1.73m2 or dependence on renal dialysis at index date | *Data sources:* SwedeHF and NPR  *Variable:* shf_gfrckdepi  *ICD-10 code:* Z99.2 Z99.2 Dependence on renal dialysis |
| 18. Hepatic impairment defined by a total bilirubin (TBL) ≥ 2 times the upper limit of normal (ULN), or alanine aminotransferase (ALT) or aspartate aminotransferase (AST) ≥ 3 times ULN at screeningHyperbilirubinemia or elevated transaminases | Hyperbilirubinemia or elevated transaminases/lactate dehydrogenase at index date | *Data source:* NPR  *ICD- 10 codes:* K70-K77, B18, R17, R74.0 K70 Alcoholic liver disease K71 Toxic liver disease K72 Hepatic failure, not elsewhere classified K73 Chronic hepatitis, not elsewhere classified K74 Fibrosis and cirrhosis of liver K75 Other inflammatory liver diseases K76 Other diseases of liver K77 Liver disorders in diseases classified elsewhere B18 Chronic viral hepatitis R17 Unspecified jaundice R74.0 Nonspecific elevation of levels of transaminase and lactic acid dehydrogenase [LDH] |
| 19. Previously received OM | Not included |  |
| 20. Severe, concomitant non-CV disease that is expected to reduce life expectancy to < 2 years | Not included |  |
| 21. Recipient of any major organ transplant (eg, lung, liver, heart, bone marrow, renal) or anticipated to receive chronic mechanical circulatory support or heart transplantation within 12 months from randomization | One of the following procedures prior to index date:  a) Admission and use of extracorporeal, paracorporeal or intracorporeal VAD  b) Heart transplantation  c) Heart and lungs transplantation  d) Lung transplantation  e) Liver transplantation  f) Kidney transplantation  g) Bone marrow transplantation | *Data source*:  NPR  *KVÅ-codes*: FQA, FQB, GDG, KAS, JJC, DR010, DR008, DR041-DR048, FXL10, FXL20, FXL30 FQA Transplantation of heart  FQB Transplantation of heart and lungs  GDG Lung transplants  KAS Kidney transplantation and related operations JJC Liver transplantation and related operations  DR010 Autologous bone marrow transplantation  DR008 Allogen benmärgstransplantation  DR041 Transplantation, bone marrow stem cells, allogeneic, related donor  DR042 Transplantation, bone marrow stem cells, allogeneic, unrelated donor  DR043 Transplantation, bone marrow stem cells, autologous  DR044 Transplantation, umbilical cord blood stem cells, related donor  DR045 Transplantation, umbilical cord blood stem cells, unrelated donor  DR046 Transplantation, peripheral blood stem cells, allogeneic, related donor  DR047 Transplantation, peripheral blood stem cells, allogeneic, unrelated donor  DR048 Transplantation, peripheral blood stem cells, autologous  FXL10 Insertion and use of extracorporeal VAD FXL20 Insertion and use of paracorporeal VAD  FXL30 Insertion and use of intracorporeal VAD  *ICD-10 codes*: Z94.0, Z94.1, Z94.3, Z94.4, Z94.6, Z94.8 Z94.0 Kidney transplant status Z94.1 Heart transplant statusZ94.2 Lung transplant status Z94.3 Heart and lungs transplant status Z94.4 Liver transplant status Z94.6 Bone transplant status Z94.8 Other transplants |
| 22. Female subject of childbearing potential who is not willing to inform her partner of her participation in this clinical study and to use 2 acceptable methods of effective birth control or practice true sexual abstinence. Male subject with a female partner of childbearing potential and not willing to inform his partner of his participation in this clinical study. | Not included |  |
| 23. Female subject is pregnant or breastfeeding or is planning to become pregnant or planning to breastfeed during treatment with OM or placebo, or within 5 days after the end of treatment | Not included |  |
| 24. Planned to be discharged from the hospital to long term care facility (e.g., skilled nursing facility) or hospice. | Not included |  |
| 25. History or evidence of any other clinically significant disorder (including cardiac arrhythmia), condition or disease (with the exception of those outlined above) that would pose a risk to subject safety or interfere with the study evaluation, procedures, or completion | Not included |  |

**Table S2. Patient characteristics for patients with ejection fraction <40% , ejection fraction <30%, in-patients, patients with severe heart failure, patients with NYHA class III-IV, and patients with NT-proBNP≥5,000pg/mL.**

| **Criterion** | **EF<40%** | **EF<30%** | **In-patients** | **Out-patients** | **Severe HF** | **NYHA III-IV** | **NT-proBNP≥5,000** | ***Missing*** |
| --- | --- | --- | --- | --- | --- | --- | --- | --- |
| *Number of patients (% of study population)* | *31,015 (100%)* | *14,805 (47.7%)* | *10,096 (32.6%)* | 20,919 (67.4%) | *5,631 (18.2%)* | *16,008 (51.6%)* | *12,077 (38.9%)* |  |
| **Sociodemographic variables** |  |  |  |  |  |  |  |  |
| Female | 8,283 (26.7%) | 3,543 (23.9%) | 3,056 (30.3%) | 5,227 (25.0%) | 1,440 (25.6%) | 4,453 (27.8%) | 3,250 (26.9%) | *0%* |
| Age, years | 76 [68.0, 82.0] | 75 [67.0, 82.0] | 79 [72.0, 85.0] | 74 [66.0, 80.0] | 78 [71.0, 83.0] | 78 [71.0, 83.0] | 78 [70.0, 84.0] | *0%* |
| Age ≥75 years | 17,004 (54.8%) | 7,898 (53.3%) | 6,772 (67.1%) | 10,232 (48.9%) | 3,591 (63.8%) | 10,105 (63.1%) | 7,495 (62.1%) | *0%* |
| Index year |  |  |  |  |  |  |  | *0%* |
| 2000-2008 | 5,005 (16.1%) | 2,671 (18.0%) | 2,698 (26.7%) | 2,307 (11.0%) | 1,345 (23.9%) | 2,926 (18.3%) | 2,456 (20.3%) |  |
| 2009-2015 | 11,684 (37.7%) | 6,137 (41.5%) | 5,350 (53.0%) | 6,334 (30.3%) | 2,636 (46.8%) | 6,539 (40.8%) | 5,423 (44.9%) |  |
| 2016-2021 | 14,326 (46.2%) | 5,997 (40.5%) | 2,048 (20.3%) | 12,278 (58.7%) | 1,650 (29.3%) | 6,543 (40.9%) | 4,198 (34.8%) |  |
| Income level <median (by index year) | 15,480 (49.9%) | 7,452 (50.4%) | 5,465 (54.2%) | 10,015 (47.9%) | 3,007 (53.4%) | 8,544 (53.4%) | 6,249 (51.8%) | *0.1%* |
| Education: compulsory school only (vs. secondary school/university) | 13,266 (43.6%) | 6,401 (44.1%) | 4,942 (50.4%) | 8,324 (40.4%) | 2,676 (48.7%) | 7,411 (47.4%) | 5,554 (47.0%) | *2%* |
| Single living | 14,633 (47.2%) | 7,001 (47.3%) | 5,332 (52.9%) | 9,301 (44.5%) | 2,717 (48.3%) | 7,787 (48.7%) | 5,855 (48.5%) | *0.1%* |
| Children | 25,791 (83.2%) | 12,224 (82.6%) | 8,322 (82.4%) | 17,469 (83.5%) | 4,622 (82.1%) | 13,317 (83.2%) | 10,029 (83.0%) | *0%* |
| **Health organizational variables** |  |  |  |  |  |  |  |  |
| Caregiver: in-patient (vs. out-patient) | 10,096 (32.6%) | 5,477 (37.0%) | 10,096 (100.0%) | 0 (0.0%) | 3,139 (55.7%) | 6,905 (43.1%) | 5,605 (46.4%) | *0%* |
| Planned follow-up: specialty care (vs. primary care/other) | 20,676 (69.9%) | 10,085 (72.0%) | 4,709 (51.1%) | 15,967 (78.4%) | 3,331 (63.7%) | 9,983 (66.2%) | 7,383 (65.3%) | *4.6%* |
| Referral to follow-up in a nurse-led HF unit | 17,221 (59.0%) | 7,967 (57.6%) | 2,958 (32.6%) | 14,263 (70.9%) | 2,490 (48.3%) | 8,311 (55.9%) | 5,674 (50.8%) | *5.9%* |
| **Clinical variables** |  |  |  |  |  |  |  |  |
| Ejection fraction <30% | 14,805 (47.7%) | 14,805 (100.0%) | 5,477 (54.2%) | 9,328 (44.6%) | 5,631 (100.0%) | 8,764 (54.7%) | 8,495 (70.3%) | *0%* |
| NYHA class |  |  |  |  |  |  |  | *20.8%* |
| I | 1,952 (7.9%) | 709 (5.9%) | 195 (3.3%) | 1,757 (9.4%) | 0 (0.0%) | 0 (0.0%) | 407 (4.5%) |  |
| II | 10,388 (42.3%) | 4,403 (36.9%) | 1,584 (26.9%) | 8,804 (47.1%) | 0 (0.0%) | 0 (0.0%) | 3,002 (32.9%) |  |
| III | 11,038 (44.9%) | 5,974 (50.1%) | 3,253 (55.2%) | 7,785 (41.7%) | 3,614 (84.9%) | 11,038 (90.3%) | 4,944 (54.1%) |  |
| IV | 1,186 (4.8%) | 850 (7.1%) | 857 (14.6%) | 329 (1.8%) | 643 (15.1%) | 1,186 (9.7%) | 779 (8.5%) |  |
| HF duration ≥6 months | 25,052 (82.0%) | 12,085 (82.6%) | 8,313 (83.4%) | 16,739 (81.3%) | 4,832 (86.7%) | 13,535 (85.7%) | 9,861 (82.7%) | *1.5%* |
| Mean arterial pressure, mmHg | 86.7 [79.3, 96.7] | 85.3 [76.7, 93.3] | 86.7 [77.0, 95.3] | 87.3 [80.0, 96.7] | 83.3 [75.0, 93.3] | 85 [76.7, 93.3] | 86.3 [76.7, 94.3] | *3.3%* |
| Mean arterial pressure <90 mmHg | 16,764 (55.9%) | 8,802 (61.3%) | 5,901 (59.1%) | 10,863 (54.3%) | 3,658 (67.0%) | 9,547 (61.5%) | 7,044 (60.1%) | *3.3%* |
| Heart rate, b.p.m. | 70 [63.0, 80.0] | 71 [64.0, 80.0] | 74 [65.0, 85.0] | 70 [61.0, 79.0] | 73 [65.0, 83.0] | 72 [64.0, 82.0] | 72 [64.0, 82.0] | *5.6%* |
| Heart rate ≥70 b.p.m. | 16,653 (56.9%) | 8,269 (59.0%) | 6,423 (66.6%) | 10,230 (52.1%) | 3,434 (64.9%) | 9,482 (62.7%) | 7,002 (61.4%) | *5.6%* |
| Body mass index, kg/m² | 26 [23.0, 29.7] | 25.6 [22.7, 29.3] | 25.2 [22.3, 29.1] | 26.4 [23.5, 30.1] | 24.8 [22.0, 28.2] | 25.9 [22.8, 29.9] | 25 [22.3, 28.4] | *11.3%* |
| Body mass index category |  |  |  |  |  |  |  | *11.3%* |
| Normal/overweight | 20,061 (73.0%) | 10,012 (75.0%) | 7,144 (74.6%) | 12,917 (72.1%) | 4,006 (77.6%) | 10,342 (71.6%) | 8,563 (78.3%) |  |
| Underweight | 836 (3.0%) | 472 (3.5%) | 469 (4.9%) | 367 (2.0%) | 248 (4.8%) | 532 (3.7%) | 459 (4.2%) |  |
| Obese | 6,602 (24.0%) | 2,871 (21.5%) | 1,961 (20.5%) | 4,641 (25.9%) | 908 (17.6%) | 3,573 (24.7%) | 1,919 (17.5%) |  |
| HF hospitalization past 6 months | 16,448 (53.0%) | 9,076 (61.3%) | 10,096 (100.0%) | 6,352 (30.4%) | 4,380 (77.8%) | 10,365 (64.7%) | 8,307 (68.8%) | *0%* |
| HF hospitalization past 12 months | 18,444 (59.5%) | 10,074 (68.0%) | 10,096 (100.0%) | 8,348 (39.9%) | 4,629 (82.2%) | 11,201 (70.0%) | 8,959 (74.2%) | *0%* |
| **Laboratory variables** |  |  |  |  |  |  |  |  |
| NT-proBNP, pg/L | 2,510 [998.8, 6150.0] | 3,471.5 [1410.0, 8133.0] | 6,624 [3000.0, 14663.0] | 1,944 [808.0, 4411.5] | 11,000 [7277.2, 19403.5] | 4,000 [1721.0, 9355.0] | 9,941 [6788.0, 17093.0] | *43.1%* |
| NT-proBNP tertile (by EF and atrial fibrillation) |  |  |  |  |  |  |  | *43.1%* |
| NT-proBNP lowest tertile | 5,886 (33.3%) | 1,947 (24.2%) | 458 (11.7%) | 5,428 (39.5%) | 0 (0.0%) | 1,849 (21.2%) | 0 (0.0%) |  |
| NT-proBNP middle tertile | 5,886 (33.3%) | 2,696 (33.5%) | 1,048 (26.7%) | 4,838 (35.2%) | 48 (2.1%) | 2,865 (32.9%) | 179 (3.3%) |  |
| NT-proBNP highest tertile | 5,888 (33.3%) | 3,403 (42.3%) | 2,419 (61.6%) | 3,469 (25.3%) | 2,220 (97.9%) | 3,995 (45.9%) | 5,183 (96.7%) |  |
| eGFR, mL/min/1.73m² | 59.3 [42.6, 79.3] | 57.9 [41.6, 77.3] | 49.9 [35.6, 68.9] | 63.7 [47.1, 83.1] | 48.5 [34.8, 65.6] | 52.2 [37.6, 70.9] | 51.3 [36.7, 70.2] | *2.5%* |
| eGFR category |  |  |  |  |  |  |  | *2.5%* |
| eGFR≥60 | 14,800 (48.9%) | 6,779 (46.8%) | 3,581 (35.6%) | 11,219 (55.6%) | 1,765 (32.0%) | 5,981 (38.1%) | 4,393 (37.4%) |  |
| eGFR 30-60 | 12,576 (41.6%) | 6,230 (43.0%) | 4,817 (47.9%) | 7,759 (38.4%) | 2,789 (50.6%) | 7,570 (48.2%) | 5,604 (47.7%) |  |
| eGFR<30 | 2,859 (9.5%) | 1,482 (10.2%) | 1,651 (16.4%) | 1,208 (6.0%) | 961 (17.4%) | 2,161 (13.8%) | 1,763 (15.0%) |  |
| Hemoglobin, g/L | 132 [120.0, 144.0] | 133 [120.0, 145.0] | 127 [114.0, 139.0] | 135 [123.0, 146.0] | 128 [116.0, 140.0] | 129 [117.0, 141.0] | 128 [116.0, 141.0] | *8.5%* |
| Potassium, mmol/L | 4.3 [4.0, 4.6] | 4.3 [4.0, 4.6] | 4.1 [3.8, 4.4] | 4.3 [4.0, 4.6] | 4.2 [3.9, 4.5] | 4.2 [3.9, 4.6] | 4.2 [3.9, 4.6] | *18.8%* |
| Potessium category |  |  |  |  |  |  |  | *18.8%* |
| Hypokalemia <5mmol/L | 846 (3.4%) | 432 (3.7%) | 502 (7.3%) | 344 (1.9%) | 248 (6.1%) | 604 (4.7%) | 465 (5.1%) |  |
| Normokalemia 3.5-4.9mmol/L | 22,538 (89.5%) | 10,372 (88.8%) | 6,032 (87.2%) | 16,506 (90.4%) | 3,502 (86.0%) | 11,132 (87.5%) | 7,977 (87.3%) |  |
| Hyperkalemia ≥5mmol/L | 1,787 (7.1%) | 878 (7.5%) | 381 (5.5%) | 1,406 (7.7%) | 322 (7.9%) | 981 (7.7%) | 695 (7.6%) |  |
| **Comorbidities** |  |  |  |  |  |  |  |  |
| Peripheral arterial disease | 3,621 (11.7%) | 1,756 (11.9%) | 1,477 (14.6%) | 2,144 (10.2%) | 843 (15.0%) | 2,278 (14.2%) | 1,673 (13.9%) | *0%* |
| Stroke/transitory ischemic attack | 5,998 (19.3%) | 2,980 (20.1%) | 2,426 (24.0%) | 3,572 (17.1%) | 1,367 (24.3%) | 3,681 (23.0%) | 2,648 (21.9%) | *0%* |
| Anemia | 10,654 (37.5%) | 5,130 (37.4%) | 4,888 (48.8%) | 5,766 (31.4%) | 2,563 (48.0%) | 6,677 (44.4%) | 5,172 (46.1%) | *8.5%* |
| Cancer past 3 years | 4,063 (13.1%) | 1,851 (12.5%) | 1,328 (13.2%) | 2,735 (13.1%) | 810 (14.4%) | 2,298 (14.4%) | 1,709 (14.2%) | *0%* |
| Liver disease | 852 (2.7%) | 449 (3.0%) | 344 (3.4%) | 508 (2.4%) | 201 (3.6%) | 494 (3.1%) | 366 (3.0%) | *0%* |
| Major bleeding | 6,437 (20.8%) | 2,987 (20.2%) | 2,488 (24.6%) | 3,949 (18.9%) | 1,432 (25.4%) | 3,877 (24.2%) | 2,948 (24.4%) | *0%* |
| Diabetes mellitus | 10,114 (32.6%) | 4,963 (33.5%) | 3,772 (37.4%) | 6,342 (30.3%) | 2,126 (37.8%) | 5,983 (37.4%) | 4,184 (34.6%) | *0%* |
| Atrial fibrillation | 18,652 (60.1%) | 8,920 (60.2%) | 6,790 (67.3%) | 11,862 (56.7%) | 3,762 (66.8%) | 10,589 (66.1%) | 7,743 (64.1%) | *0%* |
| Hypertension | 20,449 (65.9%) | 9,306 (62.9%) | 6,755 (66.9%) | 13,694 (65.5%) | 3,648 (64.8%) | 10,980 (68.6%) | 8,068 (66.8%) | *0%* |
| Chronic obstructive pulmonary disease | 4,738 (15.3%) | 2,202 (14.9%) | 1,901 (18.8%) | 2,837 (13.6%) | 1,031 (18.3%) | 3,129 (19.5%) | 1,961 (16.2%) | *0%* |
| Ischemic heart disease | 20,683 (66.7%) | 9,963 (67.3%) | 7,550 (74.8%) | 13,133 (62.8%) | 4,201 (74.6%) | 11,593 (72.4%) | 8,465 (70.1%) | *0%* |
| Revascularized | 12,841 (41.4%) | 6,013 (40.6%) | 4,225 (41.8%) | 8,616 (41.2%) | 2,458 (43.7%) | 7,050 (44.0%) | 5,001 (41.4%) | *0%* |
| Valvular disease | 9,146 (29.5%) | 4,416 (29.8%) | 3,756 (37.2%) | 5,390 (25.8%) | 2,048 (36.4%) | 5,363 (33.5%) | 4,077 (33.8%) | *0%* |
| **Treatments** |  |  |  |  |  |  |  |  |
| Beta-blockers | 28,424 (91.9%) | 13,621 (92.3%) | 8,924 (88.8%) | 19,500 (93.4%) | 5,078 (90.5%) | 14,523 (91.0%) | 10,988 (91.3%) | *0.3%* |
| RASi/ARNi | 27,259 (88.6%) | 13,011 (88.7%) | 7,789 (78.4%) | 19,470 (93.5%) | 4,596 (82.6%) | 13,353 (84.2%) | 10,080 (84.5%) | *0.8%* |
| Mineralocorticoid receptor antagonists | 15,037 (48.7%) | 7,517 (51.1%) | 4,053 (40.5%) | 10,984 (52.7%) | 2,554 (45.7%) | 7,512 (47.2%) | 5,212 (43.5%) | *0.5%* |
| SGLT2 inhibitor | 731 (34.7%) | 333 (38.8%) | 19 (16.8%) | 712 (35.7%) | 67 (35.3%) | 325 (36.0%) | 151 (31.1%) | *93.2%* |
| Diuretics | 25,260 (81.8%) | 12,694 (86.2%) | 9,167 (91.5%) | 16,093 (77.2%) | 5,226 (93.4%) | 14,391 (90.3%) | 10,684 (89.0%) | *0.4%* |
| Digoxin | 4,468 (14.5%) | 2,452 (16.6%) | 1,807 (18.0%) | 2,661 (12.8%) | 1,022 (18.2%) | 2,549 (16.0%) | 1,976 (16.4%) | *0.4%* |
| Nitrates | 4,950 (16.0%) | 2,239 (15.2%) | 2,446 (24.4%) | 2,504 (12.0%) | 1,080 (19.3%) | 3,260 (20.4%) | 2,159 (18.0%) | *0.4%* |
| Anticoagulants | 15,767 (51.0%) | 7,662 (52.0%) | 4,611 (46.0%) | 11,156 (53.5%) | 2,861 (51.1%) | 8,480 (53.2%) | 6,071 (50.6%) | *0.4%* |
| Antiplatelets | 12,600 (40.8%) | 5,915 (40.1%) | 4,880 (48.6%) | 7,720 (37.0%) | 2,379 (42.5%) | 6,479 (40.7%) | 5,065 (42.2%) | *0.4%* |
| Statins | 16,884 (54.6%) | 7,857 (53.3%) | 4,851 (48.3%) | 12,033 (57.7%) | 2,845 (50.7%) | 8,572 (53.7%) | 6,190 (51.5%) | *0.3%* |
| Cardiac resynchronisation therapy | 3,845 (12.5%) | 2,353 (16.0%) | 1,000 (10.0%) | 2,845 (13.7%) | 887 (15.9%) | 2,165 (13.6%) | 1,488 (12.4%) | *0.7%* |
| Implantable cardioverter-defibrillator | 4,814 (15.6%) | 2,826 (19.2%) | 1,099 (11.0%) | 3,715 (17.9%) | 875 (15.6%) | 2,456 (15.5%) | 1,588 (13.2%) | *0.7%* |

**Abbreviations:** ARNi, angiotensin receptor–neprilysin inhibitor; b.p.m., beats per minute; eGFR, estimated glomerular filtration rate (calculated by the Chronic Kidney Disease Epidemiology Collaboration formula); HF, heart failure; NT-proBNP, N-terminal pro-B-type natriuretic peptide; NYHA, New York Heart Association; RASi, renin–angiotensin system inhibitor; SGLT2, sodium-glucose co-transporter 2

**Table S3*.* Eligibity for omecamtiv mecarbil according to all criteria in the trial and pragmatic scenarios in sensitivity analyses for missing data.** All criteria were applied in the trial scenario. Only criteria with **bold underlined font** were applied in the pragmatic scenario.

| **Criterion** | Complete cases | Missing as eligible |
| --- | --- | --- |
| *Number of patients (% of study population)* | *13,964 (45%)* | *31,015 (100%)* |
| **Inclusion** |  |  |
| 1. Informed consent (assumed 100%) | 100.0% | 100.0% |
| 2. Male/female, 18-85 years | 89.8% | 86.7% |
| **3. Requiring HF treatment ≥30 days (assumed 100% in patients with HF duration ≥3 months)** | 100.0% | 100.0% |
| 4. EF measurement not within 30 days of HF debute or event likely to affect EF | 96.7% | 94.4% |
| **5. NYHA class II-IV** | 91.5% | 93.7% |
| 6. Receiving OMT, unless contraindicated (assumed 100% in patients with HF duration ≥3 months) | 100.0% | 100.0% |
| **7. Currently hospitalized for HF or experienced worsening HF event in the past 1 year** | 49.1% | 59.5% |
| **8. Elevated natriuretic peptides ≥400pg/mL or ≥1,200pg/mL in patients with atrial fibrillation** | 80.0% | 89.2% |
| *All inclusion criteria trial scenario* | *33.8%* | *41.1%* |
| *All inclusion criteria pragmatic scenario* | *39.2%* | *49.7%* |
| **Exclusion** |  |  |
| 1. Receiving other investigational device/treatment (assumed 100%) | 100.0% | 100.0% |
| 2. Malignancy within 5 years prior | 90.5% | 90.8% |
| 3. Known sensitivity to OM (assumed 100%) | 100.0% | 100.0% |
| 4. Condition likely to interfer, e.g. dementia, mental disorders, substance abuse | 92.5% | 91.5% |
| 5. Inability to swallow (assumed 100%) | 100.0% | 100.0% |
| 6. mechanical hemodynamical support or invasive ventilation past 7 days(assumed 100%) | 100.0% | 100.0% |
| 7. IV inotropes or vasopressors past 3 days (assumed 100%) | 100.0% | 100.0% |
| 8. IV diuretics or vasodilators, oxygen therapy, NIV, or CPAP, in past 12 hours (assumed 100%) | 100.0% | 100.0% |
| 9. ACS, stroke/TIA, or major cardiac surgery/intervention past 3 months | 94.8% | 92.2% |
| 10. Insertion of other cardiac devices past 1 month | 98.2% | 97.5% |
| 11. Uncorrected valvulopathy, HCM/infiltrative cardiomyopathy, myocarditis, congenital heart disease | 96.5% | 96.8% |
| 12. Untreated severe ventricular arrhythmia (assumed 100%) | 100.0% | 100.0% |
| 13. Chronic antiarrhythmics, excluding amiodarone/digoxin/calcium blocker/BB (assumed 100%) | 100.0% | 100.0% |
| 14. Symptomatic bradycardia or 2nd-3rd degree heart block without pacemaker (assumed 100%) | 100.0% | 100.0% |
| 15. Routine outpatient IV infusions for HF (assumed 100%) | 100.0% | 100.0% |
| **16. Systolic BP not 85-140mmHg, diastolic BP >90mmHg, or heart rate not 50-110 b.p.m.** | 82.7% | 82.1% |
| **17. eGFR<20mL/min/1.73m2, or patient on renal dialysis** | 98.2% | 97.3% |
| 18. Hepatic impairment | 99.3% | 99.1% |
| 19. Previously received OM (assumed 100%) | 100.0% | 100.0% |
| 20. Non-CV comorbidity reducing life expectancy to <2 years (assumed 100%) | 100.0% | 100.0% |
| 21. Major organ transplant or planned chronic mechanical support / heart transplant | 99.4% | 99.3% |
| 22. Patient or patient's partner of childbearing potential (assumed 100%) | 100.0% | 100.0% |
| 23. Current or planned pregnancy / breastfeeding (assumed 100%) | 100.0% | 100.0% |
| 24. Planned discharge to long term care facility or hospice (assumed 100%) | 100.0% | 100.0% |
| 25. Other condition likely to interfere with safety or ability to adhere (assumed 100%) | 100.0% | 100.0% |
| *All exclusion criteria trial scenario* | *60.7%* | *57.1%* |
| *All exclusion criteria pragmatic scenario* | *81.4%* | *80.2%* |
| **Eligibility** |  |  |
| *All criteria trial scenario* | *19.9%* | *23.1%* |
| *All criteria pragmatic scenario* | *31.7%* | *39.3%* |

**Abbreviations:** ACS, acute coronary syndrome; BP, blood pressure; CV, cardiovascular; CPAP, continuous positive airway pressure; EF, ejection fraction; eGFR, estimated glomerular filtration rate; HCM, hypertrophic cardiomyopathy; HF, heart failure; NIV, non-invasive ventilation; NT-proBNP, N-terminal pro-B-type natriuretic peptide; NYHA, New York Heart Association; OM, omecamtiv mecarbil; TIA, transient ischemic attack

**Table S4. Baseline characteristics according to eligibility for omecamtiv mecarbil (trial and pragmatic scenarios) in patients with ejection fraction <30%.**

|  | **Trial scenario** | | | **Pragmatic scenario** | | |
| --- | --- | --- | --- | --- | --- | --- |
| **Criterion** | **Ineligible** | **Eligible** | ***P*** | **Ineligible** | **Eligible** | ***P*** |
| *Number of patients (% of study population)* | *10,798 (72.9%)* | *4,007 (27.1%)* |  | *8,312 (56.1%)* | *6,493 (43.9%)* |  |
| **Sociodemographic variables** |  |  |  |  |  |  |
| Female | 2,595 (24.0%) | 948 (23.7%) | 0.651 | 1,985 (23.9%) | 1,558 (24.0%) | 0.887 |
| Age, years | 76 [67.0, 83.0] | 75 [68.0, 80.0] | <0.001 | 75 [67.0, 81.0] | 76 [68.0, 82.0] | <0.001 |
| Age ≥75 years | 5,791 (53.6%) | 2,107 (52.6%) | 0.264 | 4,202 (50.6%) | 3,696 (56.9%) | <0.001 |
| Index year |  |  | <0.001 |  |  | <0.001 |
| 2000-2008 | 1,842 (17.1%) | 829 (20.7%) |  | 1,358 (16.3%) | 1,313 (20.2%) |  |
| 2009-2015 | 4,270 (39.5%) | 1,867 (46.6%) |  | 3,100 (37.3%) | 3,037 (46.8%) |  |
| 2016-2021 | 4,686 (43.4%) | 1,311 (32.7%) |  | 3,854 (46.4%) | 2,143 (33.0%) |  |
| Income level <median (by index year) | 5,446 (50.5%) | 2,006 (50.1%) | 0.731 | 4,082 (49.2%) | 3,370 (52.0%) | 0.001 |
| Education: compulsory school only (vs. secondary school/university) | 4,643 (43.8%) | 1,758 (44.9%) | 0.239 | 3,495 (42.8%) | 2,906 (45.8%) | <0.001 |
| Single living | 5,216 (48.3%) | 1,785 (44.6%) | <0.001 | 3,862 (46.5%) | 3,139 (48.4%) | 0.024 |
| Children | 8,935 (82.7%) | 3,289 (82.1%) | 0.356 | 6,904 (83.1%) | 5,320 (81.9%) | 0.077 |
| **Health organizational variables** |  |  |  |  |  |  |
| Caregiver: in-patient (vs. out-patient) | 3,604 (33.4%) | 1,873 (46.7%) | <0.001 | 2,093 (25.2%) | 3,384 (52.1%) | <0.001 |
| Planned follow-up: specialty care (vs. primary care/other) | 7,265 (71.2%) | 2,820 (74.1%) | 0.001 | 5,910 (74.7%) | 4,175 (68.5%) | <0.001 |
| Referral to follow-up in a nurse-led HF unit | 5,850 (58.0%) | 2,117 (56.6%) | 0.123 | 4,801 (61.4%) | 3,166 (52.7%) | <0.001 |
| **Clinical variables** |  |  |  |  |  |  |
| Ejection fraction <30% | 10,798 (100.0%) | 4,007 (100.0%) | NaN | 8,312 (100.0%) | 6,493 (100.0%) | NaN |
| NYHA class |  |  | <0.001 |  |  | <0.001 |
| I | 709 (8.1%) | 0 (0.0%) |  | 709 (10.3%) | 0 (0.0%) |  |
| II | 3,314 (38.0%) | 1,089 (33.8%) |  | 2,775 (40.2%) | 1,628 (32.3%) |  |
| III | 4,143 (47.5%) | 1,831 (56.8%) |  | 3,059 (44.3%) | 2,915 (57.9%) |  |
| IV | 547 (6.3%) | 303 (9.4%) |  | 359 (5.2%) | 491 (9.8%) |  |
| HF duration ≥6 months | 8,814 (82.7%) | 3,271 (82.4%) | 0.715 | 6,776 (82.6%) | 5,309 (82.6%) | 1 |
| Mean arterial pressure, mmHg | 86.7 [77.7, 95.8] | 83.3 [76.7, 90.0] | <0.001 | 87.3 [78.3, 96.7] | 83.3 [76.7, 90.0] | <0.001 |
| Mean arterial pressure <90 mmHg | 5,977 (57.3%) | 2,825 (71.8%) | <0.001 | 4,285 (53.7%) | 4,517 (70.7%) | <0.001 |
| Heart rate, b.p.m. | 71 [63.0, 80.0] | 72 [64.0, 81.0] | <0.001 | 70 [62.0, 80.0] | 72.5 [65.0, 82.0] | <0.001 |
| Heart rate ≥70 b.p.m. | 5,884 (57.6%) | 2,385 (62.8%) | <0.001 | 4,301 (55.0%) | 3,968 (64.2%) | <0.001 |
| Body mass index, kg/m² | 25.6 [22.6, 29.3] | 25.7 [22.7, 29.3] | 0.586 | 25.9 [23.0, 29.7] | 25.1 [22.3, 28.7] | <0.001 |
| Body mass index category |  |  | 0.941 |  |  | <0.001 |
| Normal/overweight | 7,206 (75.0%) | 2,806 (74.8%) |  | 5,352 (73.5%) | 4,660 (76.7%) |  |
| Underweight | 340 (3.5%) | 132 (3.5%) |  | 214 (2.9%) | 258 (4.2%) |  |
| Obese | 2,057 (21.4%) | 814 (21.7%) |  | 1,712 (23.5%) | 1,159 (19.1%) |  |
| HF hospitalization past 6 months | 5,510 (51.0%) | 3,566 (89.0%) | <0.001 | 3,212 (38.6%) | 5,864 (90.3%) | <0.001 |
| HF hospitalization past 12 months | 6,067 (56.2%) | 4,007 (100.0%) | <0.001 | 3,581 (43.1%) | 6,493 (100.0%) | <0.001 |
| **Laboratory variables** |  |  |  |  |  |  |
| NT-proBNP, pg/L | 2,922.5 [1140.0, 7437.0] | 4,822.5 [2437.8, 9702.0] | <0.001 | 2,269 [924.0, 5654.0] | 5,304 [2656.0, 11237.0] | <0.001 |
| NT-proBNP tertile (by EF and atrial fibrillation) |  |  | <0.001 |  |  | <0.001 |
| NT-proBNP lowest tertile | 1,738 (29.4%) | 209 (9.8%) |  | 1,635 (35.5%) | 312 (9.1%) |  |
| NT-proBNP middle tertile | 1,896 (32.1%) | 800 (37.4%) |  | 1,514 (32.9%) | 1,182 (34.3%) |  |
| NT-proBNP highest tertile | 2,272 (38.5%) | 1,131 (52.9%) |  | 1,452 (31.6%) | 1,951 (56.6%) |  |
| eGFR, mL/min/1.73m² | 58.7 [41.7, 78.5] | 56 [41.4, 74.7] | <0.001 | 60.3 [42.8, 80.0] | 55.1 [40.4, 73.9] | <0.001 |
| eGFR category |  |  | <0.001 |  |  | <0.001 |
| eGFR≥60 | 5,055 (48.0%) | 1,724 (43.5%) |  | 4,086 (50.6%) | 2,693 (42.0%) |  |
| eGFR 30-60 | 4,347 (41.3%) | 1,883 (47.5%) |  | 3,113 (38.5%) | 3,117 (48.6%) |  |
| eGFR<30 | 1,128 (10.7%) | 354 (8.9%) |  | 878 (10.9%) | 604 (9.4%) |  |
| Hemoglobin, g/L | 132 [120.0, 145.0] | 133 [120.0, 144.0] | 0.765 | 134 [121.0, 145.0] | 131 [119.0, 143.0] | <0.001 |
| Potassium, mmol/L | 4.3 [4.0, 4.6] | 4.2 [3.9, 4.5] | <0.001 | 4.3 [4.0, 4.6] | 4.2 [3.9, 4.5] | <0.001 |
| Potessium category |  |  | 0.044 |  |  | <0.001 |
| Hypokalemia <5mmol/L | 296 (3.4%) | 136 (4.4%) |  | 193 (2.9%) | 239 (4.8%) |  |
| Normokalemia 3.5-4.9mmol/L | 7,663 (89.0%) | 2,709 (88.2%) |  | 5,990 (89.1%) | 4,382 (88.4%) |  |
| Hyperkalemia ≥5mmol/L | 651 (7.6%) | 227 (7.4%) |  | 540 (8.0%) | 338 (6.8%) |  |
| **Comorbidities** |  |  |  |  |  |  |
| Peripheral arterial disease | 1,269 (11.8%) | 487 (12.2%) | 0.52 | 979 (11.8%) | 777 (12.0%) | 0.744 |
| Stroke/transitory ischemic attack | 2,206 (20.4%) | 774 (19.3%) | 0.139 | 1,622 (19.5%) | 1,358 (20.9%) | 0.037 |
| Anemia | 3,719 (37.4%) | 1,411 (37.4%) | 0.974 | 2,624 (34.8%) | 2,506 (40.7%) | <0.001 |
| Cancer past 3 years | 1,646 (15.2%) | 205 (5.1%) | <0.001 | 1,032 (12.4%) | 819 (12.6%) | 0.737 |
| Liver disease | 377 (3.5%) | 72 (1.8%) | <0.001 | 204 (2.5%) | 245 (3.8%) | <0.001 |
| Major bleeding | 2,201 (20.4%) | 786 (19.6%) | 0.312 | 1,560 (18.8%) | 1,427 (22.0%) | <0.001 |
| Diabetes mellitus | 3,425 (31.7%) | 1,538 (38.4%) | <0.001 | 2,646 (31.8%) | 2,317 (35.7%) | <0.001 |
| Atrial fibrillation | 6,504 (60.2%) | 2,416 (60.3%) | 0.961 | 4,926 (59.3%) | 3,994 (61.5%) | 0.006 |
| Hypertension | 6,817 (63.1%) | 2,489 (62.1%) | 0.264 | 5,263 (63.3%) | 4,043 (62.3%) | 0.195 |
| Chronic obstructive pulmonary disease | 1,515 (14.0%) | 687 (17.1%) | <0.001 | 1,142 (13.7%) | 1,060 (16.3%) | <0.001 |
| Ischemic heart disease | 7,183 (66.5%) | 2,780 (69.4%) | 0.001 | 5,453 (65.6%) | 4,510 (69.5%) | <0.001 |
| Revascularized | 4,290 (39.7%) | 1,723 (43.0%) | <0.001 | 3,337 (40.1%) | 2,676 (41.2%) | 0.195 |
| Valvular disease | 3,140 (29.1%) | 1,276 (31.8%) | 0.001 | 2,246 (27.0%) | 2,170 (33.4%) | <0.001 |
| **Treatments** |  |  |  |  |  |  |
| Beta-blockers | 9,880 (91.8%) | 3,741 (93.6%) | <0.001 | 7,686 (92.7%) | 5,935 (91.7%) | 0.015 |
| RASi/ARNi | 9,432 (88.2%) | 3,579 (90.2%) | <0.001 | 7,396 (89.7%) | 5,615 (87.5%) | <0.001 |
| Mineralocorticoid receptor antagonists | 5,335 (49.7%) | 2,182 (54.8%) | <0.001 | 4,176 (50.5%) | 3,341 (51.8%) | 0.125 |
| SGLT2 inhibitor | 245 (35.8%) | 88 (50.6%) | <0.001 | 209 (35.8%) | 124 (45.1%) | 0.011 |
| Diuretics | 9,010 (83.9%) | 3,684 (92.4%) | <0.001 | 6,726 (81.3%) | 5,968 (92.5%) | <0.001 |
| Digoxin | 1,704 (15.8%) | 748 (18.7%) | <0.001 | 1,294 (15.6%) | 1,158 (17.9%) | <0.001 |
| Nitrates | 1,651 (15.3%) | 588 (14.7%) | 0.376 | 1,202 (14.5%) | 1,037 (16.0%) | 0.011 |
| Anticoagulants | 5,434 (50.6%) | 2,228 (55.9%) | <0.001 | 4,298 (51.9%) | 3,364 (52.1%) | 0.89 |
| Antiplatelets | 4,383 (40.8%) | 1,532 (38.4%) | 0.011 | 3,313 (40.0%) | 2,602 (40.3%) | 0.786 |
| Statins | 5,615 (52.2%) | 2,242 (56.1%) | <0.001 | 4,569 (55.2%) | 3,288 (50.8%) | <0.001 |
| Cardiac resynchronisation therapy | 1,589 (14.8%) | 764 (19.1%) | <0.001 | 1,293 (15.7%) | 1,060 (16.4%) | 0.239 |
| Implantable cardioverter-defibrillator | 1,995 (18.6%) | 831 (20.8%) | 0.003 | 1,638 (19.9%) | 1,188 (18.4%) | 0.026 |

**Abbreviations:** ARNi, angiotensin receptor–neprilysin inhibitor; b.p.m., beats per minute; eGFR, estimated glomerular filtration rate (calculated by the Chronic Kidney Disease Epidemiology Collaboration formula); HF, heart failure; NT-proBNP, N-terminal pro-B-type natriuretic peptide; NYHA, New York Heart Association; RASi, renin–angiotensin system inhibitor; SGLT2, sodium-glucose co-transporter 2

**Table S5. Baseline characteristics according to eligibility for omecamtiv mecarbil (trial and pragmatic scenarios) in in-patients.**

|  | **Trial scenario** | | | **Pragmatic scenario** | | |
| --- | --- | --- | --- | --- | --- | --- |
| **Criterion** | **Ineligible** | **Eligible** | ***P*** | **Ineligible** | **Eligible** | ***P*** |
| *Number of patients (% of study population)* | *7,084 (70.2%)* | *3,012 (29.8%)* |  | *4,328 (42.9%)* | *5,768 (57.1%)* |  |
| **Sociodemographic variables** |  |  |  |  |  |  |
| Female | 2,215 (31.3%) | 841 (27.9%) | 0.001 | 1,392 (32.2%) | 1,664 (28.8%) | <0.001 |
| Age, years | 81 [72.0, 87.0] | 77 [70.0, 82.0] | <0.001 | 79 [71.0, 85.0] | 79 [72.0, 85.0] | 0.605 |
| Age ≥75 years | 4,892 (69.1%) | 1,880 (62.4%) | <0.001 | 2,870 (66.3%) | 3,902 (67.6%) | 0.164 |
| Index year |  |  | 0.07 |  |  | <0.001 |
| 2000-2008 | 1,898 (26.8%) | 800 (26.6%) |  | 1,243 (28.7%) | 1,455 (25.2%) |  |
| 2009-2015 | 3,710 (52.4%) | 1,640 (54.4%) |  | 2,231 (51.5%) | 3,119 (54.1%) |  |
| 2016-2021 | 1,476 (20.8%) | 572 (19.0%) |  | 854 (19.7%) | 1,194 (20.7%) |  |
| Income level <median (by index year) | 3,892 (55.0%) | 1,573 (52.3%) | 0.013 | 2,366 (54.7%) | 3,099 (53.8%) | 0.346 |
| Education: compulsory school only (vs. secondary school/university) | 3,533 (51.3%) | 1,409 (48.3%) | 0.007 | 2,153 (51.3%) | 2,789 (49.7%) | 0.129 |
| Single living | 3,879 (54.8%) | 1,453 (48.3%) | <0.001 | 2,292 (53.0%) | 3,040 (52.7%) | 0.798 |
| Children | 5,868 (82.8%) | 2,454 (81.5%) | 0.106 | 3,594 (83.0%) | 4,728 (82.0%) | 0.17 |
| **Health organizational variables** |  |  |  |  |  |  |
| Caregiver: in-patient (vs. out-patient) | 7,084 (100.0%) | 3,012 (100.0%) | NaN | 4,328 (100.0%) | 5,768 (100.0%) | NaN |
| Planned follow-up: specialty care (vs. primary care/other) | 3,073 (47.8%) | 1,636 (58.6%) | <0.001 | 2,006 (51.2%) | 2,703 (51.0%) | 0.874 |
| Referral to follow-up in a nurse-led HF unit | 1,869 (29.6%) | 1,089 (39.7%) | <0.001 | 1,197 (31.1%) | 1,761 (33.8%) | 0.007 |
| **Clinical variables** |  |  |  |  |  |  |
| Ejection fraction <30% | 3,604 (50.9%) | 1,873 (62.2%) | <0.001 | 2,093 (48.4%) | 3,384 (58.7%) | <0.001 |
| NYHA class |  |  | <0.001 |  |  | <0.001 |
| I | 195 (4.9%) | 0 (0.0%) |  | 195 (7.9%) | 0 (0.0%) |  |
| II | 1,092 (27.4%) | 492 (25.8%) |  | 633 (25.7%) | 951 (27.7%) |  |
| III | 2,131 (53.5%) | 1,122 (58.9%) |  | 1,278 (51.9%) | 1,975 (57.6%) |  |
| IV | 565 (14.2%) | 292 (15.3%) |  | 355 (14.4%) | 502 (14.6%) |  |
| HF duration ≥6 months | 5,790 (82.9%) | 2,523 (84.6%) | 0.043 | 3,492 (82.0%) | 4,821 (84.4%) | 0.001 |
| Mean arterial pressure, mmHg | 86.7 [78.3, 96.7] | 83.3 [76.7, 91.7] | <0.001 | 90 [79.7, 101.3] | 83.7 [76.7, 92.3] | <0.001 |
| Mean arterial pressure <90 mmHg | 3,857 (55.1%) | 2,044 (68.5%) | <0.001 | 2,079 (48.7%) | 3,822 (66.9%) | <0.001 |
| Heart rate, b.p.m. | 74 [65.0, 85.0] | 74 [66.0, 84.0] | 0.408 | 74 [65.0, 86.0] | 75 [66.0, 84.0] | 0.187 |
| Heart rate ≥70 b.p.m. | 4,491 (66.2%) | 1,932 (67.6%) | 0.201 | 2,666 (64.4%) | 3,757 (68.3%) | <0.001 |
| Body mass index, kg/m² | 25 [22.1, 28.7] | 25.7 [22.7, 29.8] | <0.001 | 25.3 [22.4, 29.2] | 25.1 [22.2, 28.9] | 0.024 |
| Body mass index category |  |  | <0.001 |  |  | 0.103 |
| Normal/overweight | 5,101 (76.3%) | 2,043 (70.8%) |  | 3,040 (74.7%) | 4,104 (74.5%) |  |
| Underweight | 332 (5.0%) | 137 (4.7%) |  | 178 (4.4%) | 291 (5.3%) |  |
| Obese | 1,256 (18.8%) | 705 (24.4%) |  | 850 (20.9%) | 1,111 (20.2%) |  |
| HF hospitalization past 6 months | 7,084 (100.0%) | 3,012 (100.0%) | NaN | 4,328 (100.0%) | 5,768 (100.0%) | NaN |
| HF hospitalization past 12 months | 7,084 (100.0%) | 3,012 (100.0%) | NaN | 4,328 (100.0%) | 5,768 (100.0%) | NaN |
| **Laboratory variables** |  |  |  |  |  |  |
| NT-proBNP, pg/L | 6,920 [2960.0, 15779.5] | 6,185.5 [3126.8, 12692.0] | 0.162 | 5,797 [2080.0, 15100.0] | 6,906 [3480.0, 14406.8] | <0.001 |
| NT-proBNP tertile (by EF and atrial fibrillation) |  |  | <0.001 |  |  | <0.001 |
| NT-proBNP lowest tertile | 349 (13.4%) | 109 (8.3%) |  | 273 (19.5%) | 185 (7.3%) |  |
| NT-proBNP middle tertile | 637 (24.4%) | 411 (31.2%) |  | 328 (23.4%) | 720 (28.5%) |  |
| NT-proBNP highest tertile | 1,621 (62.2%) | 798 (60.5%) |  | 800 (57.1%) | 1,619 (64.1%) |  |
| eGFR, mL/min/1.73m² | 49.1 [34.2, 68.8] | 51.8 [38.2, 69.4] | <0.001 | 48.9 [32.1, 69.8] | 50.7 [37.4, 68.5] | <0.001 |
| eGFR category |  |  | <0.001 |  |  | <0.001 |
| eGFR≥60 | 2,473 (35.1%) | 1,108 (37.0%) |  | 1,540 (35.8%) | 2,041 (35.5%) |  |
| eGFR 30-60 | 3,275 (46.4%) | 1,542 (51.5%) |  | 1,810 (42.0%) | 3,007 (52.4%) |  |
| eGFR<30 | 1,304 (18.5%) | 347 (11.6%) |  | 956 (22.2%) | 695 (12.1%) |  |
| Hemoglobin, g/L | 126 [114.0, 138.0] | 129 [117.0, 141.0] | <0.001 | 125 [114.0, 139.0] | 128 [115.0, 140.0] | <0.001 |
| Potassium, mmol/L | 4.1 [3.8, 4.4] | 4.1 [3.8, 4.4] | 0.414 | 4.1 [3.8, 4.5] | 4.1 [3.8, 4.4] | 0.001 |
| Potessium category |  |  | 0.448 |  |  | 0.009 |
| Hypokalemia <5mmol/L | 353 (7.3%) | 149 (7.2%) |  | 196 (6.8%) | 306 (7.6%) |  |
| Normokalemia 3.5-4.9mmol/L | 4,203 (87.0%) | 1,829 (87.8%) |  | 2,513 (86.8%) | 3,519 (87.5%) |  |
| Hyperkalemia ≥5mmol/L | 277 (5.7%) | 104 (5.0%) |  | 186 (6.4%) | 195 (4.9%) |  |
| **Comorbidities** |  |  |  |  |  |  |
| Peripheral arterial disease | 1,026 (14.5%) | 451 (15.0%) | 0.544 | 691 (16.0%) | 786 (13.6%) | 0.001 |
| Stroke/transitory ischemic attack | 1,783 (25.2%) | 643 (21.3%) | <0.001 | 1,063 (24.6%) | 1,363 (23.6%) | 0.289 |
| Anemia | 3,561 (50.7%) | 1,327 (44.5%) | <0.001 | 2,168 (50.5%) | 2,720 (47.6%) | 0.004 |
| Cancer past 3 years | 1,157 (16.3%) | 171 (5.7%) | <0.001 | 559 (12.9%) | 769 (13.3%) | 0.56 |
| Liver disease | 289 (4.1%) | 55 (1.8%) | <0.001 | 137 (3.2%) | 207 (3.6%) | 0.269 |
| Major bleeding | 1,804 (25.5%) | 684 (22.7%) | 0.004 | 1,067 (24.7%) | 1,421 (24.6%) | 1 |
| Diabetes mellitus | 2,519 (35.6%) | 1,253 (41.6%) | <0.001 | 1,649 (38.1%) | 2,123 (36.8%) | 0.19 |
| Atrial fibrillation | 4,748 (67.0%) | 2,042 (67.8%) | 0.464 | 2,872 (66.4%) | 3,918 (67.9%) | 0.101 |
| Hypertension | 4,828 (68.2%) | 1,927 (64.0%) | <0.001 | 3,023 (69.8%) | 3,732 (64.7%) | <0.001 |
| Chronic obstructive pulmonary disease | 1,270 (17.9%) | 631 (20.9%) | <0.001 | 789 (18.2%) | 1,112 (19.3%) | 0.191 |
| Ischemic heart disease | 5,301 (74.8%) | 2,249 (74.7%) | 0.883 | 3,317 (76.6%) | 4,233 (73.4%) | <0.001 |
| Revascularized | 2,839 (40.1%) | 1,386 (46.0%) | <0.001 | 1,819 (42.0%) | 2,406 (41.7%) | 0.766 |
| Valvular disease | 2,616 (36.9%) | 1,140 (37.8%) | 0.394 | 1,525 (35.2%) | 2,231 (38.7%) | <0.001 |
| **Treatments** |  |  |  |  |  |  |
| Beta-blockers | 6,204 (88.0%) | 2,720 (90.7%) | <0.001 | 3,833 (89.0%) | 5,091 (88.6%) | 0.607 |
| RASi/ARNi | 5,285 (75.9%) | 2,504 (84.3%) | <0.001 | 3,243 (76.1%) | 4,546 (80.1%) | <0.001 |
| Mineralocorticoid receptor antagonists | 2,666 (37.9%) | 1,387 (46.5%) | <0.001 | 1,594 (37.1%) | 2,459 (43.0%) | <0.001 |
| SGLT2 inhibitor | 13 (15.1%) | 6 (22.2%) | 0.571 | 8 (15.1%) | 11 (18.3%) | 0.836 |
| Diuretics | 6,370 (90.6%) | 2,797 (93.5%) | <0.001 | 3,824 (89.0%) | 5,343 (93.3%) | <0.001 |
| Digoxin | 1,160 (16.5%) | 647 (21.6%) | <0.001 | 712 (16.5%) | 1,095 (19.1%) | 0.001 |
| Nitrates | 1,841 (26.2%) | 605 (20.2%) | <0.001 | 1,209 (28.1%) | 1,237 (21.6%) | <0.001 |
| Anticoagulants | 2,956 (42.0%) | 1,655 (55.3%) | <0.001 | 1,815 (42.2%) | 2,796 (48.8%) | <0.001 |
| Antiplatelets | 3,639 (51.6%) | 1,241 (41.5%) | <0.001 | 2,322 (53.9%) | 2,558 (44.7%) | <0.001 |
| Statins | 3,247 (46.0%) | 1,604 (53.5%) | <0.001 | 2,170 (50.4%) | 2,681 (46.7%) | <0.001 |
| Cardiac resynchronisation therapy | 606 (8.6%) | 394 (13.1%) | <0.001 | 402 (9.4%) | 598 (10.4%) | 0.083 |
| Implantable cardioverter-defibrillator | 660 (9.4%) | 439 (14.6%) | <0.001 | 411 (9.6%) | 688 (12.0%) | <0.001 |

**Abbreviations:** ARNi, angiotensin receptor–neprilysin inhibitor; b.p.m., beats per minute; eGFR, estimated glomerular filtration rate (calculated by the Chronic Kidney Disease Epidemiology Collaboration formula); HF, heart failure; NT-proBNP, N-terminal pro-B-type natriuretic peptide; NYHA, New York Heart Association; RASi, renin–angiotensin system inhibitor; SGLT2, sodium-glucose co-transporter 2

**Table S6. Baseline characteristics according to eligibility for omecamtiv mecarbil (trial and pragmatic scenarios) in out-patients.**

|  | **Trial scenario** | | | **Pragmatic scenario** | | |
| --- | --- | --- | --- | --- | --- | --- |
| **Criterion** | **Ineligible** | **Eligible** | ***P*** | **Ineligible** | **Eligible** | ***P*** |
| *Number of patients (% of study population)* | *17,392 (83.1%)* | *3,527 (16.9%)* |  | *15,646 (74.8%)* | *5,273 (25.2%)* |  |
| **Sociodemographic variables** |  |  |  |  |  |  |
| Female | 4,326 (24.9%) | 901 (25.5%) | 0.412 | 3,891 (24.9%) | 1,336 (25.3%) | 0.509 |
| Age, years | 74 [66.0, 81.0] | 75 [67.0, 80.0] | 0.551 | 74 [66.0, 80.0] | 76 [68.0, 82.0] | <0.001 |
| Age ≥75 years | 8,459 (48.6%) | 1,773 (50.3%) | 0.08 | 7,365 (47.1%) | 2,867 (54.4%) | <0.001 |
| Index year |  |  | <0.001 |  |  | <0.001 |
| 2000-2008 | 1,813 (10.4%) | 494 (14.0%) |  | 1,623 (10.4%) | 684 (13.0%) |  |
| 2009-2015 | 5,049 (29.0%) | 1,285 (36.4%) |  | 4,451 (28.4%) | 1,883 (35.7%) |  |
| 2016-2021 | 10,530 (60.5%) | 1,748 (49.6%) |  | 9,572 (61.2%) | 2,706 (51.3%) |  |
| Income level <median (by index year) | 8,228 (47.3%) | 1,787 (50.7%) | <0.001 | 7,308 (46.7%) | 2,707 (51.4%) | <0.001 |
| Education: compulsory school only (vs. secondary school/university) | 6,811 (39.8%) | 1,513 (43.7%) | <0.001 | 6,040 (39.2%) | 2,284 (44.1%) | <0.001 |
| Single living | 7,753 (44.6%) | 1,548 (43.9%) | 0.483 | 6,848 (43.8%) | 2,453 (46.6%) | 0.001 |
| Children | 14,539 (83.6%) | 2,930 (83.1%) | 0.461 | 13,088 (83.7%) | 4,381 (83.1%) | 0.348 |
| **Health organizational variables** |  |  |  |  |  |  |
| Caregiver: in-patient (vs. out-patient) | 0 (0.0%) | 0 (0.0%) | NaN | 0 (0.0%) | 0 (0.0%) | NaN |
| Planned follow-up: specialty care (vs. primary care/other) | 13,146 (77.7%) | 2,821 (82.3%) | <0.001 | 11,929 (78.2%) | 4,038 (79.2%) | 0.124 |
| Referral to follow-up in a nurse-led HF unit | 11,919 (71.2%) | 2,344 (69.3%) | 0.032 | 10,781 (71.5%) | 3,482 (69.0%) | 0.001 |
| **Clinical variables** |  |  |  |  |  |  |
| Ejection fraction <30% | 7,194 (41.4%) | 2,134 (60.5%) | <0.001 | 6,219 (39.7%) | 3,109 (59.0%) | <0.001 |
| NYHA class |  |  | <0.001 |  |  | <0.001 |
| I | 1,757 (11.4%) | 0 (0.0%) |  | 1,757 (12.7%) | 0 (0.0%) |  |
| II | 7,377 (47.7%) | 1,427 (44.3%) |  | 6,792 (48.9%) | 2,012 (41.9%) |  |
| III | 6,074 (39.3%) | 1,711 (53.1%) |  | 5,136 (37.0%) | 2,649 (55.2%) |  |
| IV | 246 (1.6%) | 83 (2.6%) |  | 193 (1.4%) | 136 (2.8%) |  |
| HF duration ≥6 months | 13,991 (81.8%) | 2,748 (78.7%) | <0.001 | 12,621 (82.1%) | 4,118 (78.9%) | <0.001 |
| Mean arterial pressure, mmHg | 88.3 [80.0, 96.7] | 84 [76.7, 91.7] | <0.001 | 90 [80.0, 97.7] | 83.3 [76.7, 91.7] | <0.001 |
| Mean arterial pressure <90 mmHg | 8,558 (51.5%) | 2,305 (67.7%) | <0.001 | 7,384 (49.5%) | 3,479 (68.4%) | <0.001 |
| Heart rate, b.p.m. | 70 [61.0, 78.0] | 70 [63.0, 80.0] | <0.001 | 70 [60.0, 78.0] | 70 [63.0, 80.0] | <0.001 |
| Heart rate ≥70 b.p.m. | 8,340 (51.2%) | 1,890 (56.7%) | <0.001 | 7,365 (50.3%) | 2,865 (57.4%) | <0.001 |
| Body mass index, kg/m² | 26.5 [23.5, 30.1] | 26.3 [23.4, 30.0] | 0.087 | 26.7 [23.7, 30.4] | 25.9 [23.0, 29.4] | <0.001 |
| Body mass index category |  |  | 0.75 |  |  | <0.001 |
| Normal/overweight | 10,593 (71.9%) | 2,324 (72.6%) |  | 9,329 (70.9%) | 3,588 (75.1%) |  |
| Underweight | 301 (2.0%) | 66 (2.1%) |  | 250 (1.9%) | 117 (2.5%) |  |
| Obese | 3,829 (26.0%) | 812 (25.4%) |  | 3,571 (27.2%) | 1,070 (22.4%) |  |
| HF hospitalization past 6 months | 3,608 (20.7%) | 2,744 (77.8%) | <0.001 | 2,217 (14.2%) | 4,135 (78.4%) | <0.001 |
| HF hospitalization past 12 months | 4,821 (27.7%) | 3,527 (100.0%) | <0.001 | 3,075 (19.7%) | 5,273 (100.0%) | <0.001 |
| **Laboratory variables** |  |  |  |  |  |  |
| NT-proBNP, pg/L | 1,670.5 [692.0, 3902.5] | 3,420 [1848.5, 6593.5] | <0.001 | 1,460 [617.0, 3423.5] | 3,663.5 [1940.0, 7290.0] | <0.001 |
| NT-proBNP tertile (by EF and atrial fibrillation) |  |  | <0.001 |  |  | <0.001 |
| NT-proBNP lowest tertile | 5,065 (44.2%) | 363 (15.9%) |  | 4,906 (47.9%) | 522 (15.0%) |  |
| NT-proBNP middle tertile | 3,832 (33.5%) | 1,006 (44.1%) |  | 3,369 (32.9%) | 1,469 (42.1%) |  |
| NT-proBNP highest tertile | 2,555 (22.3%) | 914 (40.0%) |  | 1,972 (19.2%) | 1,497 (42.9%) |  |
| eGFR, mL/min/1.73m² | 64.7 [47.8, 84.2] | 59 [44.1, 77.6] | <0.001 | 65.9 [48.7, 84.9] | 58.1 [43.3, 76.6] | <0.001 |
| eGFR category |  |  | <0.001 |  |  | <0.001 |
| eGFR≥60 | 9,552 (57.1%) | 1,667 (48.3%) |  | 8,826 (58.7%) | 2,393 (46.4%) |  |
| eGFR 30-60 | 6,202 (37.1%) | 1,557 (45.1%) |  | 5,362 (35.7%) | 2,397 (46.5%) |  |
| eGFR<30 | 978 (5.8%) | 230 (6.7%) |  | 846 (5.6%) | 362 (7.0%) |  |
| Hemoglobin, g/L | 135 [124.0, 146.0] | 133 [121.0, 145.0] | <0.001 | 136 [124.0, 147.0] | 132 [120.0, 143.0] | <0.001 |
| Potassium, mmol/L | 4.3 [4.1, 4.6] | 4.3 [4.0, 4.6] | 0.104 | 4.3 [4.1, 4.6] | 4.3 [4.0, 4.6] | 0.002 |
| Potessium category |  |  | 0.009 |  |  | <0.001 |
| Hypokalemia <5mmol/L | 275 (1.8%) | 69 (2.3%) |  | 230 (1.7%) | 114 (2.5%) |  |
| Normokalemia 3.5-4.9mmol/L | 13,830 (90.7%) | 2,676 (88.9%) |  | 12,456 (90.9%) | 4,050 (89.0%) |  |
| Hyperkalemia ≥5mmol/L | 1,142 (7.5%) | 264 (8.8%) |  | 1,017 (7.4%) | 389 (8.5%) |  |
| **Comorbidities** |  |  |  |  |  |  |
| Peripheral arterial disease | 1,748 (10.1%) | 396 (11.2%) | 0.038 | 1,546 (9.9%) | 598 (11.3%) | 0.003 |
| Stroke/transitory ischemic attack | 2,974 (17.1%) | 598 (17.0%) | 0.854 | 2,581 (16.5%) | 991 (18.8%) | <0.001 |
| Anemia | 4,666 (30.7%) | 1,100 (34.8%) | <0.001 | 3,956 (29.0%) | 1,810 (38.1%) | <0.001 |
| Cancer past 3 years | 2,556 (14.7%) | 179 (5.1%) | <0.001 | 2,011 (12.9%) | 724 (13.7%) | 0.107 |
| Liver disease | 437 (2.5%) | 71 (2.0%) | 0.09 | 330 (2.1%) | 178 (3.4%) | <0.001 |
| Major bleeding | 3,258 (18.7%) | 691 (19.6%) | 0.244 | 2,769 (17.7%) | 1,180 (22.4%) | <0.001 |
| Diabetes mellitus | 5,057 (29.1%) | 1,285 (36.4%) | <0.001 | 4,509 (28.8%) | 1,833 (34.8%) | <0.001 |
| Atrial fibrillation | 9,809 (56.4%) | 2,053 (58.2%) | 0.05 | 8,712 (55.7%) | 3,150 (59.7%) | <0.001 |
| Hypertension | 11,351 (65.3%) | 2,343 (66.4%) | 0.191 | 10,190 (65.1%) | 3,504 (66.5%) | 0.083 |
| Chronic obstructive pulmonary disease | 2,247 (12.9%) | 590 (16.7%) | <0.001 | 1,964 (12.6%) | 873 (16.6%) | <0.001 |
| Ischemic heart disease | 10,865 (62.5%) | 2,268 (64.3%) | 0.042 | 9,705 (62.0%) | 3,428 (65.0%) | <0.001 |
| Revascularized | 7,149 (41.1%) | 1,467 (41.6%) | 0.604 | 6,437 (41.1%) | 2,179 (41.3%) | 0.829 |
| Valvular disease | 4,376 (25.2%) | 1,014 (28.7%) | <0.001 | 3,798 (24.3%) | 1,592 (30.2%) | <0.001 |
| **Treatments** |  |  |  |  |  |  |
| Beta-blockers | 16,147 (93.0%) | 3,353 (95.2%) | <0.001 | 14,535 (93.1%) | 4,965 (94.3%) | 0.002 |
| RASi/ARNi | 16,165 (93.4%) | 3,305 (94.1%) | 0.122 | 14,623 (93.9%) | 4,847 (92.4%) | <0.001 |
| MRA | 8,885 (51.3%) | 2,099 (59.7%) | <0.001 | 7,961 (51.0%) | 3,023 (57.5%) | <0.001 |
| SGLT2 inhibitor | 595 (34.5%) | 117 (43.3%) | 0.006 | 540 (34.2%) | 172 (41.1%) | 0.01 |
| Diuretics | 12,911 (74.5%) | 3,182 (90.4%) | <0.001 | 11,323 (72.6%) | 4,770 (90.7%) | <0.001 |
| Digoxin | 2,132 (12.3%) | 529 (15.0%) | <0.001 | 1,888 (12.1%) | 773 (14.7%) | <0.001 |
| Nitrates | 2,066 (11.9%) | 438 (12.4%) | 0.388 | 1,798 (11.5%) | 706 (13.4%) | <0.001 |
| Anticoagulants | 9,155 (52.8%) | 2,001 (56.9%) | <0.001 | 8,230 (52.7%) | 2,926 (55.7%) | <0.001 |
| Antiplatelets | 6,483 (37.4%) | 1,237 (35.2%) | 0.014 | 5,844 (37.5%) | 1,876 (35.7%) | 0.022 |
| Statins | 9,966 (57.5%) | 2,067 (58.8%) | 0.164 | 9,134 (58.5%) | 2,899 (55.1%) | <0.001 |
| Cardiac resynchronisation therapy | 2,227 (12.9%) | 618 (17.6%) | <0.001 | 2,016 (13.0%) | 829 (15.8%) | <0.001 |
| Implantable cardioverter-defibrillator | 3,034 (17.6%) | 681 (19.4%) | 0.013 | 2,782 (17.9%) | 933 (17.8%) | 0.862 |

**Abbreviations:** ARNi, angiotensin receptor–neprilysin inhibitor; b.p.m., beats per minute; eGFR, estimated glomerular filtration rate (calculated by the Chronic Kidney Disease Epidemiology Collaboration formula); HF, heart failure; NT-proBNP, N-terminal pro-B-type natriuretic peptide; NYHA, New York Heart Association; RASi, renin–angiotensin system inhibitor; SGLT2, sodium-glucose co-transporter 2

**Table S7. Baseline characteristics according to eligibility for omecamtiv mecarbil (trial and pragmatic scenarios) in patients with severe heart failure.**

|  | **Trial scenario** | | | **Pragmatic scenario** | | |
| --- | --- | --- | --- | --- | --- | --- |
| **Criterion** | **Ineligible** | **Eligible** | ***P*** | **Ineligible** | **Eligible** | ***P*** |
| *Number of patients (% of study population)* | *3,662 (65%)* | *1,969 (35%)* |  | *2,270 (40.3%)* | *3,361 (59.7%)* |  |
| **Sociodemographic variables** |  |  |  |  |  |  |
| Female | 995 (27.2%) | 445 (22.6%) | <0.001 | 631 (27.8%) | 809 (24.1%) | 0.002 |
| Age, years | 79 [71.0, 85.0] | 77 [70.0, 81.0] | <0.001 | 78 [71.0, 83.0] | 78 [71.0, 83.0] | 0.378 |
| Age ≥75 years | 2,393 (65.3%) | 1,198 (60.8%) | 0.001 | 1,423 (62.7%) | 2,168 (64.5%) | 0.173 |
| Index year |  |  | <0.001 |  |  | <0.001 |
| 2000-2008 | 834 (22.8%) | 511 (26.0%) |  | 511 (22.5%) | 834 (24.8%) |  |
| 2009-2015 | 1,661 (45.4%) | 975 (49.5%) |  | 958 (42.2%) | 1,678 (49.9%) |  |
| 2016-2021 | 1,167 (31.9%) | 483 (24.5%) |  | 801 (35.3%) | 849 (25.3%) |  |
| Income level <median (by index year) | 2,001 (54.7%) | 1,006 (51.1%) | 0.011 | 1,222 (53.9%) | 1,785 (53.1%) | 0.584 |
| Education: compulsory school only (vs. secondary school/university) | 1,743 (48.8%) | 933 (48.7%) | 0.959 | 1,074 (48.5%) | 1,602 (48.9%) | 0.797 |
| Single living | 1,834 (50.1%) | 883 (44.9%) | <0.001 | 1,083 (47.8%) | 1,634 (48.6%) | 0.545 |
| Children | 3,015 (82.3%) | 1,607 (81.6%) | 0.527 | 1,887 (83.1%) | 2,735 (81.4%) | 0.1 |
| **Health organizational variables** |  |  |  |  |  |  |
| Caregiver: in-patient (vs. out-patient) | 1,919 (52.4%) | 1,220 (62.0%) | <0.001 | 957 (42.2%) | 2,182 (64.9%) | <0.001 |
| Planned follow-up: specialty care (vs. primary care/other) | 2,077 (61.5%) | 1,254 (67.7%) | <0.001 | 1,418 (67.1%) | 1,913 (61.4%) | <0.001 |
| Referral to follow-up in a nurse-led HF unit | 1,591 (47.7%) | 899 (49.4%) | 0.255 | 1,113 (53.3%) | 1,377 (45.0%) | <0.001 |
| **Clinical variables** |  |  |  |  |  |  |
| Ejection fraction <30% | 3,662 (100.0%) | 1,969 (100.0%) | NaN | 2,270 (100.0%) | 3,361 (100.0%) | NaN |
| NYHA class |  |  | NaN |  |  | NaN |
| I | 0 (0.0%) | 0 (0.0%) |  | 0 (0.0%) | 0 (0.0%) |  |
| II | 0 (0.0%) | 0 (0.0%) |  | 0 (0.0%) | 0 (0.0%) |  |
| III | 2,349 (85.7%) | 1,265 (83.4%) |  | 1,519 (86.7%) | 2,095 (83.7%) |  |
| IV | 392 (14.3%) | 251 (16.6%) |  | 234 (13.3%) | 409 (16.3%) |  |
| HF duration ≥6 months | 3,137 (86.6%) | 1,695 (86.8%) | 0.846 | 1,944 (86.7%) | 2,888 (86.7%) | 1 |
| Mean arterial pressure, mmHg | 83.7 [76.3, 93.3] | 81.7 [75.0, 90.0] | <0.001 | 86.7 [76.7, 96.7] | 82 [75.0, 90.0] | <0.001 |
| Mean arterial pressure <90 mmHg | 2,215 (62.8%) | 1,443 (74.5%) | <0.001 | 1,237 (57.3%) | 2,421 (73.3%) | <0.001 |
| Heart rate, b.p.m. | 72 [65.0, 84.0] | 74 [66.0, 82.0] | 0.472 | 72 [64.0, 83.0] | 74 [66.0, 83.0] | <0.001 |
| Heart rate ≥70 b.p.m. | 2,194 (63.8%) | 1,240 (67.0%) | 0.024 | 1,275 (60.5%) | 2,159 (67.9%) | <0.001 |
| Body mass index, kg/m² | 24.7 [21.9, 28.0] | 25 [22.2, 28.4] | 0.009 | 25.1 [22.2, 28.8] | 24.6 [21.9, 27.8] | <0.001 |
| Body mass index category |  |  | 0.125 |  |  | 0.002 |
| Normal/overweight | 2,572 (78.1%) | 1,434 (76.7%) |  | 1,504 (75.7%) | 2,502 (78.8%) |  |
| Underweight | 166 (5.0%) | 82 (4.4%) |  | 87 (4.4%) | 161 (5.1%) |  |
| Obese | 555 (16.9%) | 353 (18.9%) |  | 395 (19.9%) | 513 (16.2%) |  |
| HF hospitalization past 6 months | 2,541 (69.4%) | 1,839 (93.4%) | <0.001 | 1,214 (53.5%) | 3,166 (94.2%) | <0.001 |
| HF hospitalization past 12 months | 2,660 (72.6%) | 1,969 (100.0%) | <0.001 | 1,268 (55.9%) | 3,361 (100.0%) | <0.001 |
| **Laboratory variables** |  |  |  |  |  |  |
| NT-proBNP, pg/L | 11,400 [7420.8, 20000.0] | 10,396 [7065.0, 17991.0] | 0.01 | 10,890 [7123.5, 19000.0] | 11,159 [7312.0, 19637.0] | 0.535 |
| NT-proBNP tertile (by EF and atrial fibrillation) |  |  | NaN |  |  | NaN |
| NT-proBNP lowest tertile | 0 (0.0%) | 0 (0.0%) |  | 0 (0.0%) | 0 (0.0%) |  |
| NT-proBNP middle tertile | 26 (1.8%) | 22 (2.7%) |  | 20 (2.3%) | 28 (2.0%) |  |
| NT-proBNP highest tertile | 1,436 (98.2%) | 784 (97.3%) |  | 835 (97.7%) | 1,385 (98.0%) |  |
| eGFR, mL/min/1.73m² | 47.9 [33.5, 65.5] | 49.4 [36.5, 65.7] | 0.001 | 47.3 [31.4, 65.7] | 49 [36.3, 65.5] | <0.001 |
| eGFR category |  |  | <0.001 |  |  | <0.001 |
| eGFR≥60 | 1,129 (31.7%) | 636 (32.6%) |  | 713 (32.5%) | 1,052 (31.7%) |  |
| eGFR 30-60 | 1,742 (48.9%) | 1,047 (53.7%) |  | 978 (44.6%) | 1,811 (54.5%) |  |
| eGFR<30 | 695 (19.5%) | 266 (13.6%) |  | 501 (22.9%) | 460 (13.8%) |  |
| Hemoglobin, g/L | 127 [115.0, 140.0] | 130 [118.0, 141.0] | <0.001 | 127 [115.0, 140.0] | 128 [116.0, 141.0] | 0.026 |
| Potassium, mmol/L | 4.2 [3.9, 4.6] | 4.2 [3.9, 4.5] | 0.002 | 4.2 [3.9, 4.6] | 4.2 [3.8, 4.5] | <0.001 |
| Potessium category |  |  | 0.226 |  |  | <0.001 |
| Hypokalemia <5mmol/L | 150 (5.6%) | 98 (7.0%) |  | 75 (4.5%) | 173 (7.2%) |  |
| Normokalemia 3.5-4.9mmol/L | 2,302 (86.3%) | 1,200 (85.3%) |  | 1,425 (86.1%) | 2,077 (86.0%) |  |
| Hyperkalemia ≥5mmol/L | 214 (8.0%) | 108 (7.7%) |  | 156 (9.4%) | 166 (6.9%) |  |
| **Comorbidities** |  |  |  |  |  |  |
| Peripheral arterial disease | 556 (15.2%) | 287 (14.6%) | 0.569 | 370 (16.3%) | 473 (14.1%) | 0.024 |
| Stroke/transitory ischemic attack | 927 (25.3%) | 440 (22.3%) | 0.015 | 577 (25.4%) | 790 (23.5%) | 0.107 |
| Anemia | 1,724 (50.1%) | 839 (44.3%) | <0.001 | 1,022 (48.9%) | 1,541 (47.5%) | 0.309 |
| Cancer past 3 years | 692 (18.9%) | 118 (6.0%) | <0.001 | 324 (14.3%) | 486 (14.5%) | 0.875 |
| Liver disease | 162 (4.4%) | 39 (2.0%) | <0.001 | 77 (3.4%) | 124 (3.7%) | 0.605 |
| Major bleeding | 989 (27.0%) | 443 (22.5%) | <0.001 | 595 (26.2%) | 837 (24.9%) | 0.283 |
| Diabetes mellitus | 1,299 (35.5%) | 827 (42.0%) | <0.001 | 838 (36.9%) | 1,288 (38.3%) | 0.299 |
| Atrial fibrillation | 2,406 (65.7%) | 1,356 (68.9%) | 0.018 | 1,447 (63.7%) | 2,315 (68.9%) | <0.001 |
| Hypertension | 2,417 (66.0%) | 1,231 (62.5%) | 0.01 | 1,547 (68.1%) | 2,101 (62.5%) | <0.001 |
| Chronic obstructive pulmonary disease | 646 (17.6%) | 385 (19.6%) | 0.083 | 405 (17.8%) | 626 (18.6%) | 0.477 |
| Ischemic heart disease | 2,731 (74.6%) | 1,470 (74.7%) | 0.973 | 1,715 (75.6%) | 2,486 (74.0%) | 0.191 |
| Revascularized | 1,553 (42.4%) | 905 (46.0%) | 0.011 | 1,010 (44.5%) | 1,448 (43.1%) | 0.308 |
| Valvular disease | 1,326 (36.2%) | 722 (36.7%) | 0.755 | 765 (33.7%) | 1,283 (38.2%) | 0.001 |
| **Treatments** |  |  |  |  |  |  |
| Beta-blockers | 3,277 (89.8%) | 1,801 (91.9%) | 0.013 | 2,070 (91.6%) | 3,008 (89.8%) | 0.036 |
| RASi/ARNi | 2,915 (80.4%) | 1,681 (86.5%) | <0.001 | 1,838 (81.6%) | 2,758 (83.2%) | 0.136 |
| Mineralocorticoid receptor antagonists | 1,592 (43.8%) | 962 (49.3%) | <0.001 | 984 (43.6%) | 1,570 (47.1%) | 0.011 |
| SGLT2 inhibitor | 49 (33.6%) | 18 (40.9%) | 0.475 | 33 (31.1%) | 34 (40.5%) | 0.236 |
| Diuretics | 3,365 (92.5%) | 1,861 (95.0%) | <0.001 | 2,053 (91.0%) | 3,173 (95.1%) | <0.001 |
| Digoxin | 608 (16.7%) | 414 (21.1%) | <0.001 | 373 (16.5%) | 649 (19.4%) | 0.006 |
| Nitrates | 743 (20.4%) | 337 (17.2%) | 0.005 | 476 (21.0%) | 604 (18.1%) | 0.007 |
| Anticoagulants | 1,728 (47.4%) | 1,133 (58.0%) | <0.001 | 1,106 (49.0%) | 1,755 (52.6%) | 0.008 |
| Antiplatelets | 1,619 (44.4%) | 760 (38.9%) | <0.001 | 1,013 (44.8%) | 1,366 (40.9%) | 0.004 |
| Statins | 1,759 (48.2%) | 1,086 (55.4%) | <0.001 | 1,217 (53.8%) | 1,628 (48.6%) | <0.001 |
| Cardiac resynchronisation therapy | 501 (13.8%) | 386 (19.7%) | <0.001 | 329 (14.6%) | 558 (16.7%) | 0.039 |
| Implantable cardioverter-defibrillator | 486 (13.4%) | 389 (19.9%) | <0.001 | 315 (14.0%) | 560 (16.8%) | 0.006 |

**Abbreviations:** ARNi, angiotensin receptor–neprilysin inhibitor; b.p.m., beats per minute; eGFR, estimated glomerular filtration rate (calculated by the Chronic Kidney Disease Epidemiology Collaboration formula); HF, heart failure; NT-proBNP, N-terminal pro-B-type natriuretic peptide; NYHA, New York Heart Association; RASi, renin–angiotensin system inhibitor; SGLT2, sodium-glucose co-transporter 2

**Table S8. Baseline characteristics according to eligibility for omecamtiv mecarbil (trial and pragmatic scenarios) in patients with NYHA class III-IV.**

|  | **Trial scenario** | | | **Pragmatic scenario** | | |
| --- | --- | --- | --- | --- | --- | --- |
| **Criterion** | **Ineligible** | **Eligible** | ***P*** | **Ineligible** | **Eligible** | ***P*** |
| *Number of patients (% of study population)* | *11,835 (73.9%)* | *4,173 (26.1%)* |  | *8,841 (55.2%)* | *7,167 (44.8%)* |  |
| **Sociodemographic variables** |  |  |  |  |  |  |
| Female | 3,379 (28.6%) | 1,074 (25.7%) | 0.001 | 2,524 (28.5%) | 1,929 (26.9%) | 0.023 |
| Age, years | 78 [71.0, 85.0] | 77 [70.0, 81.0] | <0.001 | 77 [70.0, 83.0] | 78 [71.0, 84.0] | <0.001 |
| Age ≥75 years | 7,585 (64.1%) | 2,520 (60.4%) | <0.001 | 5,423 (61.3%) | 4,682 (65.3%) | <0.001 |
| Index year |  |  | <0.001 |  |  | <0.001 |
| 2000-2008 | 2,043 (17.3%) | 883 (21.2%) |  | 1,454 (16.4%) | 1,472 (20.5%) |  |
| 2009-2015 | 4,652 (39.3%) | 1,887 (45.2%) |  | 3,254 (36.8%) | 3,285 (45.8%) |  |
| 2016-2021 | 5,140 (43.4%) | 1,403 (33.6%) |  | 4,133 (46.7%) | 2,410 (33.6%) |  |
| Income level <median (by index year) | 6,318 (53.4%) | 2,226 (53.4%) | 0.984 | 4,660 (52.7%) | 3,884 (54.2%) | 0.065 |
| Education: compulsory school only (vs. secondary school/university) | 5,433 (47.0%) | 1,978 (48.7%) | 0.064 | 3,965 (45.9%) | 3,446 (49.3%) | <0.001 |
| Single living | 5,858 (49.5%) | 1,929 (46.3%) | <0.001 | 4,230 (47.9%) | 3,557 (49.7%) | 0.026 |
| Children | 9,881 (83.5%) | 3,436 (82.3%) | 0.092 | 7,408 (83.8%) | 5,909 (82.4%) | 0.025 |
| **Health organizational variables** |  |  |  |  |  |  |
| Caregiver: in-patient (vs. out-patient) | 4,694 (39.7%) | 2,211 (53.0%) | <0.001 | 2,793 (31.6%) | 4,112 (57.4%) | <0.001 |
| Planned follow-up: specialty care (vs. primary care/other) | 7,247 (65.1%) | 2,736 (69.2%) | <0.001 | 5,802 (69.2%) | 4,181 (62.3%) | <0.001 |
| Referral to follow-up in a nurse-led HF unit | 6,166 (56.1%) | 2,145 (55.2%) | 0.314 | 5,017 (60.7%) | 3,294 (49.8%) | <0.001 |
| **Clinical variables** |  |  |  |  |  |  |
| Ejection fraction <30% | 6,056 (51.2%) | 2,708 (64.9%) | <0.001 | 4,290 (48.5%) | 4,474 (62.4%) | <0.001 |
| NYHA class |  |  | NaN |  |  | NaN |
| I | 0 (0.0%) | 0 (0.0%) |  | 0 (0.0%) | 0 (0.0%) |  |
| II | 0 (0.0%) | 0 (0.0%) |  | 0 (0.0%) | 0 (0.0%) |  |
| III | 8,205 (91.0%) | 2,833 (88.3%) |  | 6,414 (92.1%) | 4,624 (87.9%) |  |
| IV | 811 (9.0%) | 375 (11.7%) |  | 548 (7.9%) | 638 (12.1%) |  |
| HF duration ≥6 months | 9,997 (85.7%) | 3,538 (85.6%) | 0.971 | 7,473 (85.8%) | 6,062 (85.5%) | 0.53 |
| Mean arterial pressure, mmHg | 86.7 [77.0, 96.0] | 83.3 [76.7, 90.0] | <0.001 | 86.7 [78.3, 96.7] | 83.3 [76.7, 90.0] | <0.001 |
| Mean arterial pressure <90 mmHg | 6,636 (58.0%) | 2,911 (71.2%) | <0.001 | 4,580 (53.8%) | 4,967 (70.7%) | <0.001 |
| Heart rate, b.p.m. | 72 [64.0, 82.0] | 73 [65.0, 81.0] | 0.009 | 71 [64.0, 81.0] | 73 [66.0, 82.0] | <0.001 |
| Heart rate ≥70 b.p.m. | 6,908 (61.8%) | 2,574 (65.2%) | <0.001 | 4,972 (59.7%) | 4,510 (66.3%) | <0.001 |
| Body mass index, kg/m² | 25.9 [22.8, 29.8] | 25.9 [22.9, 30.1] | 0.187 | 26.4 [23.2, 30.4] | 25.4 [22.4, 29.2] | <0.001 |
| Body mass index category |  |  | 0.171 |  |  | <0.001 |
| Normal/overweight | 7,577 (72.0%) | 2,765 (70.5%) |  | 5,359 (69.4%) | 4,983 (74.1%) |  |
| Underweight | 386 (3.7%) | 146 (3.7%) |  | 240 (3.1%) | 292 (4.3%) |  |
| Obese | 2,560 (24.3%) | 1,013 (25.8%) |  | 2,125 (27.5%) | 1,448 (21.5%) |  |
| HF hospitalization past 6 months | 6,568 (55.5%) | 3,797 (91.0%) | <0.001 | 3,761 (42.5%) | 6,604 (92.1%) | <0.001 |
| HF hospitalization past 12 months | 7,028 (59.4%) | 4,173 (100.0%) | <0.001 | 4,034 (45.6%) | 7,167 (100.0%) | <0.001 |
| **Laboratory variables** |  |  |  |  |  |  |
| NT-proBNP, pg/L | 3,580 [1420.0, 8990.2] | 5,079 [2586.0, 10100.0] | <0.001 | 2,784 [1110.0, 6960.0] | 5,689 [2846.5, 11423.8] | <0.001 |
| NT-proBNP tertile (by EF and atrial fibrillation) |  |  | <0.001 |  |  | <0.001 |
| NT-proBNP lowest tertile | 1,633 (25.5%) | 216 (9.4%) |  | 1,518 (31.6%) | 331 (8.5%) |  |
| NT-proBNP middle tertile | 2,016 (31.5%) | 849 (36.9%) |  | 1,566 (32.6%) | 1,299 (33.3%) |  |
| NT-proBNP highest tertile | 2,759 (43.1%) | 1,236 (53.7%) |  | 1,721 (35.8%) | 2,274 (58.2%) |  |
| eGFR, mL/min/1.73m² | 52.5 [37.3, 71.8] | 51.5 [38.4, 68.6] | 0.509 | 53.6 [37.6, 73.4] | 50.5 [37.5, 67.7] | <0.001 |
| eGFR category |  |  | <0.001 |  |  | <0.001 |
| eGFR≥60 | 4,490 (38.8%) | 1,491 (36.1%) |  | 3,537 (41.0%) | 2,444 (34.5%) |  |
| eGFR 30-60 | 5,392 (46.6%) | 2,178 (52.7%) |  | 3,777 (43.8%) | 3,793 (53.5%) |  |
| eGFR<30 | 1,695 (14.6%) | 466 (11.3%) |  | 1,306 (15.2%) | 855 (12.1%) |  |
| Hemoglobin, g/L | 129 [117.0, 141.0] | 129 [118.0, 141.0] | 0.072 | 130 [118.0, 142.0] | 128 [116.0, 140.0] | <0.001 |
| Potassium, mmol/L | 4.3 [3.9, 4.6] | 4.2 [3.9, 4.5] | <0.001 | 4.3 [4.0, 4.6] | 4.2 [3.9, 4.5] | <0.001 |
| Potessium category |  |  | 0.136 |  |  | <0.001 |
| Hypokalemia <5mmol/L | 431 (4.5%) | 173 (5.3%) |  | 271 (3.8%) | 333 (6.0%) |  |
| Normokalemia 3.5-4.9mmol/L | 8,305 (87.6%) | 2,827 (87.3%) |  | 6,287 (87.8%) | 4,845 (87.2%) |  |
| Hyperkalemia ≥5mmol/L | 743 (7.8%) | 238 (7.4%) |  | 604 (8.4%) | 377 (6.8%) |  |
| **Comorbidities** |  |  |  |  |  |  |
| Peripheral arterial disease | 1,655 (14.0%) | 623 (14.9%) | 0.14 | 1,263 (14.3%) | 1,015 (14.2%) | 0.842 |
| Stroke/transitory ischemic attack | 2,791 (23.6%) | 890 (21.3%) | 0.003 | 2,000 (22.6%) | 1,681 (23.5%) | 0.22 |
| Anemia | 4,947 (44.7%) | 1,730 (43.4%) | 0.14 | 3,457 (42.4%) | 3,220 (46.7%) | <0.001 |
| Cancer past 3 years | 2,073 (17.5%) | 225 (5.4%) | <0.001 | 1,286 (14.5%) | 1,012 (14.1%) | 0.459 |
| Liver disease | 401 (3.4%) | 93 (2.2%) | <0.001 | 245 (2.8%) | 249 (3.5%) | 0.012 |
| Major bleeding | 2,925 (24.7%) | 952 (22.8%) | 0.015 | 2,068 (23.4%) | 1,809 (25.2%) | 0.007 |
| Diabetes mellitus | 4,224 (35.7%) | 1,759 (42.2%) | <0.001 | 3,233 (36.6%) | 2,750 (38.4%) | 0.02 |
| Atrial fibrillation | 7,807 (66.0%) | 2,782 (66.7%) | 0.421 | 5,754 (65.1%) | 4,835 (67.5%) | 0.002 |
| Hypertension | 8,208 (69.4%) | 2,772 (66.4%) | <0.001 | 6,231 (70.5%) | 4,749 (66.3%) | <0.001 |
| Chronic obstructive pulmonary disease | 2,230 (18.8%) | 899 (21.5%) | <0.001 | 1,681 (19.0%) | 1,448 (20.2%) | 0.062 |
| Ischemic heart disease | 8,508 (71.9%) | 3,085 (73.9%) | 0.012 | 6,353 (71.9%) | 5,240 (73.1%) | 0.08 |
| Revascularized | 5,091 (43.0%) | 1,959 (46.9%) | <0.001 | 3,909 (44.2%) | 3,141 (43.8%) | 0.634 |
| Valvular disease | 3,881 (32.8%) | 1,482 (35.5%) | 0.001 | 2,710 (30.7%) | 2,653 (37.0%) | <0.001 |
| **Treatments** |  |  |  |  |  |  |
| Beta-blockers | 10,699 (90.7%) | 3,824 (91.9%) | 0.021 | 8,082 (91.7%) | 6,441 (90.2%) | 0.001 |
| RASi/ARNi | 9,747 (83.2%) | 3,606 (87.2%) | <0.001 | 7,448 (84.9%) | 5,905 (83.4%) | 0.007 |
| Mineralocorticoid receptor antagonists | 5,345 (45.4%) | 2,167 (52.3%) | <0.001 | 4,023 (45.7%) | 3,489 (49.0%) | <0.001 |
| SGLT2 inhibitor | 263 (35.6%) | 62 (37.8%) | 0.665 | 223 (36.0%) | 102 (36.2%) | 1 |
| Diuretics | 10,457 (88.8%) | 3,934 (94.6%) | <0.001 | 7,640 (86.8%) | 6,751 (94.6%) | <0.001 |
| Digoxin | 1,769 (15.0%) | 780 (18.8%) | <0.001 | 1,304 (14.8%) | 1,245 (17.4%) | <0.001 |
| Nitrates | 2,487 (21.1%) | 773 (18.6%) | 0.001 | 1,828 (20.7%) | 1,432 (20.1%) | 0.301 |
| Anticoagulants | 6,067 (51.5%) | 2,413 (58.1%) | <0.001 | 4,651 (52.8%) | 3,829 (53.7%) | 0.249 |
| Antiplatelets | 4,896 (41.5%) | 1,583 (38.2%) | <0.001 | 3,631 (41.3%) | 2,848 (39.9%) | 0.094 |
| Statins | 6,205 (52.6%) | 2,367 (56.9%) | <0.001 | 4,982 (56.5%) | 3,590 (50.3%) | <0.001 |
| Cardiac resynchronisation therapy | 1,441 (12.3%) | 724 (17.4%) | <0.001 | 1,140 (13.0%) | 1,025 (14.4%) | 0.012 |
| Implantable cardioverter-defibrillator | 1,684 (14.4%) | 772 (18.6%) | <0.001 | 1,355 (15.5%) | 1,101 (15.5%) | 1 |

**Abbreviations:** ARNi, angiotensin receptor–neprilysin inhibitor; b.p.m., beats per minute; eGFR, estimated glomerular filtration rate (calculated by the Chronic Kidney Disease Epidemiology Collaboration formula); HF, heart failure; NT-proBNP, N-terminal pro-B-type natriuretic peptide; NYHA, New York Heart Association; RASi, renin–angiotensin system inhibitor; SGLT2, sodium-glucose co-transporter 2

**Table S9. Baseline characteristics according to eligibility for omecamtiv mecarbil (trial and pragmatic scenarios) in patients with NT-proBNP≥5,000pg/mL.**

|  | **Trial scenario** | | | **Pragmatic scenario** | | |
| --- | --- | --- | --- | --- | --- | --- |
| **Criterion** | **Ineligible** | **Eligible** | ***P*** | **Ineligible** | **Eligible** | ***P*** |
| *Number of patients (% of study population)* | *8,520 (70.5%)* | *3,557 (29.5%)* |  | *5,877 (48.7%)* | *6,200 (51.3%)* |  |
| **Sociodemographic variables** |  |  |  |  |  |  |
| Female | 2,340 (27.5%) | 910 (25.6%) | 0.036 | 1,612 (27.4%) | 1,638 (26.4%) | 0.219 |
| Age, years | 78 [70.0, 85.0] | 77 [70.0, 81.0] | <0.001 | 77 [69.0, 83.0] | 78 [71.0, 84.0] | <0.001 |
| Age ≥75 years | 5,373 (63.1%) | 2,122 (59.7%) | <0.001 | 3,500 (59.6%) | 3,995 (64.4%) | <0.001 |
| Index year |  |  | <0.001 |  |  | <0.001 |
| 2000-2008 | 1,641 (19.3%) | 815 (22.9%) |  | 1,133 (19.3%) | 1,323 (21.3%) |  |
| 2009-2015 | 3,697 (43.4%) | 1,726 (48.5%) |  | 2,375 (40.4%) | 3,048 (49.2%) |  |
| 2016-2021 | 3,182 (37.3%) | 1,016 (28.6%) |  | 2,369 (40.3%) | 1,829 (29.5%) |  |
| Income level <median (by index year) | 4,420 (51.9%) | 1,829 (51.5%) | 0.676 | 2,978 (50.7%) | 3,271 (52.8%) | 0.023 |
| Education: compulsory school only (vs. secondary school/university) | 3,903 (46.8%) | 1,651 (47.6%) | 0.403 | 2,626 (45.6%) | 2,928 (48.3%) | 0.003 |
| Single living | 4,219 (49.6%) | 1,636 (46.0%) | <0.001 | 2,760 (47.0%) | 3,095 (50.0%) | 0.001 |
| Children | 7,106 (83.4%) | 2,923 (82.2%) | 0.107 | 4,930 (83.9%) | 5,099 (82.2%) | 0.017 |
| **Health organizational variables** |  |  |  |  |  |  |
| Caregiver: in-patient (vs. out-patient) | 3,674 (43.1%) | 1,931 (54.3%) | <0.001 | 1,914 (32.6%) | 3,691 (59.5%) | <0.001 |
| Planned follow-up: specialty care (vs. primary care/other) | 5,056 (63.5%) | 2,327 (69.5%) | <0.001 | 3,799 (68.7%) | 3,584 (62.0%) | <0.001 |
| Referral to follow-up in a nurse-led HF unit | 3,964 (50.4%) | 1,710 (51.8%) | 0.171 | 2,987 (54.8%) | 2,687 (47.1%) | <0.001 |
| **Clinical variables** |  |  |  |  |  |  |
| Ejection fraction <30% | 5,752 (67.5%) | 2,743 (77.1%) | <0.001 | 3,884 (66.1%) | 4,611 (74.4%) | <0.001 |
| NYHA class |  |  | <0.001 |  |  | <0.001 |
| I | 407 (6.4%) | 0 (0.0%) |  | 407 (8.8%) | 0 (0.0%) |  |
| II | 2,162 (33.8%) | 840 (30.8%) |  | 1,663 (36.1%) | 1,339 (29.6%) |  |
| III | 3,341 (52.2%) | 1,603 (58.8%) |  | 2,242 (48.6%) | 2,702 (59.7%) |  |
| IV | 495 (7.7%) | 284 (10.4%) |  | 297 (6.4%) | 482 (10.7%) |  |
| HF duration ≥6 months | 6,947 (82.7%) | 2,914 (82.7%) | 0.943 | 4,757 (82.2%) | 5,104 (83.1%) | 0.183 |
| Mean arterial pressure, mmHg | 86.7 [78.3, 96.7] | 83.3 [76.7, 90.0] | <0.001 | 89.3 [79.7, 99.7] | 83.3 [76.7, 91.3] | <0.001 |
| Mean arterial pressure <90 mmHg | 4,575 (55.6%) | 2,469 (70.7%) | <0.001 | 2,825 (50.2%) | 4,219 (69.3%) | <0.001 |
| Heart rate, b.p.m. | 71 [64.0, 82.0] | 73 [65.0, 82.0] | <0.001 | 70 [62.0, 81.0] | 74 [65.0, 83.0] | <0.001 |
| Heart rate ≥70 b.p.m. | 4,825 (60.1%) | 2,177 (64.8%) | <0.001 | 3,113 (56.6%) | 3,889 (66.0%) | <0.001 |
| Body mass index, kg/m² | 24.9 [22.2, 28.2] | 25.2 [22.5, 28.7] | <0.001 | 25.4 [22.5, 28.7] | 24.8 [22.1, 28.1] | <0.001 |
| Body mass index category |  |  | <0.001 |  |  | <0.001 |
| Normal/overweight | 6,021 (79.2%) | 2,542 (76.2%) |  | 3,981 (77.7%) | 4,582 (78.8%) |  |
| Underweight | 325 (4.3%) | 134 (4.0%) |  | 184 (3.6%) | 275 (4.7%) |  |
| Obese | 1,259 (16.6%) | 660 (19.8%) |  | 958 (18.7%) | 961 (16.5%) |  |
| HF hospitalization past 6 months | 5,058 (59.4%) | 3,249 (91.3%) | <0.001 | 2,579 (43.9%) | 5,728 (92.4%) | <0.001 |
| HF hospitalization past 12 months | 5,402 (63.4%) | 3,557 (100.0%) | <0.001 | 2,759 (46.9%) | 6,200 (100.0%) | <0.001 |
| **Laboratory variables** |  |  |  |  |  |  |
| NT-proBNP, pg/L | 10,040 [6861.5, 17343.5] | 9,548 [6640.0, 16062.0] | 0.009 | 9,640 [6652.8, 16566.2] | 10,192.5 [6920.0, 17598.2] | 0.014 |
| NT-proBNP tertile (by EF and atrial fibrillation) |  |  | NaN |  |  | NaN |
| NT-proBNP lowest tertile | 0 (0.0%) | 0 (0.0%) |  | 0 (0.0%) | 0 (0.0%) |  |
| NT-proBNP middle tertile | 116 (3.1%) | 63 (4.0%) |  | 89 (3.6%) | 90 (3.1%) |  |
| NT-proBNP highest tertile | 3,663 (96.9%) | 1,520 (96.0%) |  | 2,363 (96.4%) | 2,820 (96.9%) |  |
| eGFR, mL/min/1.73m² | 51.1 [36.0, 70.6] | 51.6 [38.3, 69.4] | 0.008 | 52.3 [35.3, 72.2] | 50.7 [37.5, 68.1] | 0.958 |
| eGFR category |  |  | <0.001 |  |  | <0.001 |
| eGFR≥60 | 3,085 (37.4%) | 1,308 (37.3%) |  | 2,231 (39.6%) | 2,162 (35.3%) |  |
| eGFR 30-60 | 3,822 (46.3%) | 1,782 (50.8%) |  | 2,398 (42.5%) | 3,206 (52.4%) |  |
| eGFR<30 | 1,343 (16.3%) | 420 (12.0%) |  | 1,010 (17.9%) | 753 (12.3%) |  |
| Hemoglobin, g/L | 128 [115.0, 141.0] | 130 [117.0, 142.0] | <0.001 | 128 [116.0, 142.0] | 128 [116.0, 141.0] | 0.175 |
| Potassium, mmol/L | 4.2 [3.9, 4.6] | 4.2 [3.9, 4.5] | <0.001 | 4.3 [4.0, 4.6] | 4.2 [3.8, 4.5] | <0.001 |
| Potessium category |  |  | 0.324 |  |  | <0.001 |
| Hypokalemia <5mmol/L | 317 (4.9%) | 148 (5.6%) |  | 178 (4.0%) | 287 (6.2%) |  |
| Normokalemia 3.5-4.9mmol/L | 5,689 (87.5%) | 2,288 (86.9%) |  | 3,934 (87.6%) | 4,043 (87.0%) |  |
| Hyperkalemia ≥5mmol/L | 499 (7.7%) | 196 (7.4%) |  | 379 (8.4%) | 316 (6.8%) |  |
| **Comorbidities** |  |  |  |  |  |  |
| Peripheral arterial disease | 1,164 (13.7%) | 509 (14.3%) | 0.363 | 819 (13.9%) | 854 (13.8%) | 0.818 |
| Stroke/transitory ischemic attack | 1,907 (22.4%) | 741 (20.8%) | 0.064 | 1,260 (21.4%) | 1,388 (22.4%) | 0.216 |
| Anemia | 3,713 (47.3%) | 1,459 (43.4%) | <0.001 | 2,390 (45.1%) | 2,782 (47.0%) | 0.043 |
| Cancer past 3 years | 1,497 (17.6%) | 212 (6.0%) | <0.001 | 820 (14.0%) | 889 (14.3%) | 0.56 |
| Liver disease | 302 (3.5%) | 64 (1.8%) | <0.001 | 143 (2.4%) | 223 (3.6%) | <0.001 |
| Major bleeding | 2,148 (25.2%) | 800 (22.5%) | 0.002 | 1,402 (23.9%) | 1,546 (24.9%) | 0.174 |
| Diabetes mellitus | 2,767 (32.5%) | 1,417 (39.8%) | <0.001 | 1,936 (32.9%) | 2,248 (36.3%) | <0.001 |
| Atrial fibrillation | 5,359 (62.9%) | 2,384 (67.0%) | <0.001 | 3,568 (60.7%) | 4,175 (67.3%) | <0.001 |
| Hypertension | 5,763 (67.6%) | 2,305 (64.8%) | 0.003 | 4,024 (68.5%) | 4,044 (65.2%) | <0.001 |
| Chronic obstructive pulmonary disease | 1,301 (15.3%) | 660 (18.6%) | <0.001 | 856 (14.6%) | 1,105 (17.8%) | <0.001 |
| Ischemic heart disease | 5,963 (70.0%) | 2,502 (70.3%) | 0.716 | 4,109 (69.9%) | 4,356 (70.3%) | 0.697 |
| Revascularized | 3,453 (40.5%) | 1,548 (43.5%) | 0.003 | 2,446 (41.6%) | 2,555 (41.2%) | 0.661 |
| Valvular disease | 2,806 (32.9%) | 1,271 (35.7%) | 0.003 | 1,807 (30.7%) | 2,270 (36.6%) | <0.001 |
| **Treatments** |  |  |  |  |  |  |
| Beta-blockers | 7,687 (90.5%) | 3,301 (93.1%) | <0.001 | 5,366 (91.6%) | 5,622 (91.0%) | 0.2 |
| RASi/ARNi | 7,009 (83.2%) | 3,071 (87.4%) | <0.001 | 4,947 (85.0%) | 5,133 (83.9%) | 0.102 |
| Mineralocorticoid receptor antagonists | 3,487 (41.2%) | 1,725 (48.9%) | <0.001 | 2,379 (40.7%) | 2,833 (46.0%) | <0.001 |
| SGLT2 inhibitor | 112 (29.3%) | 39 (37.5%) | 0.139 | 84 (27.5%) | 67 (37.0%) | 0.037 |
| Diuretics | 7,380 (87.1%) | 3,304 (93.4%) | <0.001 | 4,916 (84.1%) | 5,768 (93.6%) | <0.001 |
| Digoxin | 1,282 (15.1%) | 694 (19.6%) | <0.001 | 876 (15.0%) | 1,100 (17.8%) | <0.001 |
| Nitrates | 1,583 (18.7%) | 576 (16.3%) | 0.002 | 1,067 (18.2%) | 1,092 (17.7%) | 0.457 |
| Anticoagulants | 4,041 (47.7%) | 2,030 (57.4%) | <0.001 | 2,870 (49.1%) | 3,201 (51.9%) | 0.002 |
| Antiplatelets | 3,730 (44.0%) | 1,335 (37.8%) | <0.001 | 2,568 (43.9%) | 2,497 (40.5%) | <0.001 |
| Statins | 4,241 (50.0%) | 1,949 (55.0%) | <0.001 | 3,165 (54.1%) | 3,025 (49.0%) | <0.001 |
| Cardiac resynchronisation therapy | 927 (11.0%) | 561 (15.8%) | <0.001 | 666 (11.4%) | 822 (13.3%) | 0.002 |
| Implantable cardioverter-defibrillator | 1,009 (11.9%) | 579 (16.4%) | <0.001 | 731 (12.6%) | 857 (13.9%) | 0.032 |

**Abbreviations:** ARNi, angiotensin receptor–neprilysin inhibitor; b.p.m., beats per minute; eGFR, estimated glomerular filtration rate (calculated by the Chronic Kidney Disease Epidemiology Collaboration formula); HF, heart failure; NT-proBNP, N-terminal pro-B-type natriuretic peptide; NYHA, New York Heart Association; RASi, renin–angiotensin system inhibitor; SGLT2, sodium-glucose co-transporter 2

**Table S10. Baseline characteristics according to eligibility for omecamtiv mecarbil (trial and pragmatic scenario sensitivity analysis considering as eligible also patients with hypotension).**

|  | **Trial scenario** | | | **Pragmatic scenario** | | |
| --- | --- | --- | --- | --- | --- | --- |
| **Criterion** | **Ineligible** | **Eligible** | ***P*** | **Ineligible** | **Eligible** | ***P*** |
| *Number of patients (% of study population)* | *24,245 (78.2%)* | *6,770 (21.8%)* |  | *19,611 (63.2%)* | *11,404 (36.8%)* |  |
| **Sociodemographic variables** |  |  |  |  |  |  |
| Female | 6,488 (26.8%) | 1,795 (26.5%) | 0.697 | 5,190 (26.5%) | 3,093 (27.1%) | 0.212 |
| Age, years | 76 [68.0, 83.0] | 76 [69.0, 81.0] | <0.001 | 75 [67.0, 81.0] | 78 [70.0, 83.0] | <0.001 |
| Age ≥75 years | 13,231 (54.6%) | 3,773 (55.7%) | 0.093 | 10,025 (51.1%) | 6,979 (61.2%) | <0.001 |
| Index year |  |  | <0.001 |  |  | <0.001 |
| 2000-2008 | 3,671 (15.1%) | 1,334 (19.7%) |  | 2,804 (14.3%) | 2,201 (19.3%) |  |
| 2009-2015 | 8,642 (35.6%) | 3,042 (44.9%) |  | 6,499 (33.1%) | 5,185 (45.5%) |  |
| 2016-2021 | 11,932 (49.2%) | 2,394 (35.4%) |  | 10,308 (52.6%) | 4,018 (35.2%) |  |
| Income level <median (by index year) | 12,009 (49.6%) | 3,471 (51.3%) | 0.01 | 9,494 (48.4%) | 5,986 (52.5%) | <0.001 |
| Education: compulsory school only (vs. secondary school/university) | 10,252 (43.1%) | 3,014 (45.7%) | <0.001 | 8,043 (41.8%) | 5,223 (46.9%) | <0.001 |
| Single living | 11,528 (47.6%) | 3,105 (45.9%) | 0.017 | 8,965 (45.7%) | 5,668 (49.7%) | <0.001 |
| Children | 20,218 (83.4%) | 5,573 (82.3%) | 0.039 | 16,385 (83.6%) | 9,406 (82.5%) | 0.016 |
| **Health organizational variables** |  |  |  |  |  |  |
| Caregiver: in-patient (vs. out-patient) | 6,958 (28.7%) | 3,138 (46.4%) | <0.001 | 4,130 (21.1%) | 5,966 (52.3%) | <0.001 |
| Planned follow-up: specialty care (vs. primary care/other) | 16,060 (69.4%) | 4,616 (71.8%) | <0.001 | 13,700 (72.7%) | 6,976 (65.0%) | <0.001 |
| Referral to follow-up in a nurse-led HF unit | 13,659 (59.7%) | 3,562 (56.3%) | <0.001 | 11,793 (63.3%) | 5,428 (51.3%) | <0.001 |
| **Clinical variables** |  |  |  |  |  |  |
| Ejection fraction <30% | 10,634 (43.9%) | 4,171 (61.6%) | <0.001 | 8,072 (41.2%) | 6,733 (59.0%) | <0.001 |
| NYHA class |  |  | <0.001 |  |  | <0.001 |
| I | 1,952 (10.1%) | 0 (0.0%) |  | 1,952 (12.2%) | 0 (0.0%) |  |
| II | 8,434 (43.8%) | 1,954 (36.8%) |  | 7,371 (45.9%) | 3,017 (35.5%) |  |
| III | 8,101 (42.1%) | 2,937 (55.3%) |  | 6,260 (39.0%) | 4,778 (56.2%) |  |
| IV | 769 (4.0%) | 417 (7.9%) |  | 481 (3.0%) | 705 (8.3%) |  |
| HF duration ≥6 months | 19,583 (82.1%) | 5,469 (81.6%) | 0.36 | 15,807 (82.0%) | 9,245 (81.9%) | 0.874 |
| Mean arterial pressure, mmHg | 88.3 [80.0, 96.7] | 83.3 [76.7, 91.3] | <0.001 | 90 [81.0, 98.7] | 83.3 [76.7, 91.7] | <0.001 |
| Mean arterial pressure <90 mmHg | 12,186 (52.1%) | 4,578 (69.2%) | <0.001 | 9,102 (48.3%) | 7,662 (68.6%) | <0.001 |
| Heart rate, b.p.m. | 70 [62.0, 80.0] | 72 [64.0, 81.0] | <0.001 | 70 [61.0, 80.0] | 72 [65.0, 82.0] | <0.001 |
| Heart rate ≥70 b.p.m. | 12,696 (55.5%) | 3,957 (61.7%) | <0.001 | 9,823 (53.3%) | 6,830 (63.0%) | <0.001 |
| Body mass index, kg/m² | 26.1 [23.1, 29.7] | 26 [23.0, 29.8] | 0.51 | 26.4 [23.4, 30.1] | 25.5 [22.5, 29.2] | <0.001 |
| Body mass index category |  |  | 0.018 |  |  | <0.001 |
| Normal/overweight | 15,528 (73.3%) | 4,533 (71.9%) |  | 12,109 (71.8%) | 7,952 (74.8%) |  |
| Underweight | 616 (2.9%) | 220 (3.5%) |  | 405 (2.4%) | 431 (4.1%) |  |
| Obese | 5,049 (23.8%) | 1,553 (24.6%) |  | 4,360 (25.8%) | 2,242 (21.1%) |  |
| HF hospitalization past 6 months | 10,478 (43.2%) | 5,970 (88.2%) | <0.001 | 6,211 (31.7%) | 10,237 (89.8%) | <0.001 |
| HF hospitalization past 12 months | 11,674 (48.2%) | 6,770 (100.0%) | <0.001 | 7,040 (35.9%) | 11,404 (100.0%) | <0.001 |
| **Laboratory variables** |  |  |  |  |  |  |
| NT-proBNP, pg/L | 2,074 [813.0, 5346.0] | 4,300 [2150.0, 8706.5] | <0.001 | 1,630 [671.0, 4075.0] | 4,819 [2340.0, 10000.0] | <0.001 |
| NT-proBNP tertile (by EF and atrial fibrillation) |  |  | <0.001 |  |  | <0.001 |
| NT-proBNP lowest tertile | 5,403 (38.8%) | 483 (13.0%) |  | 5,162 (45.1%) | 724 (11.7%) |  |
| NT-proBNP middle tertile | 4,432 (31.8%) | 1,454 (39.0%) |  | 3,637 (31.8%) | 2,249 (36.2%) |  |
| NT-proBNP highest tertile | 4,098 (29.4%) | 1,790 (48.0%) |  | 2,648 (23.1%) | 3,240 (52.1%) |  |
| eGFR, mL/min/1.73m² | 60.5 [43.2, 80.9] | 55.4 [40.8, 73.9] | <0.001 | 62.8 [45.1, 82.8] | 53.9 [39.6, 72.5] | <0.001 |
| eGFR category |  |  | <0.001 |  |  | <0.001 |
| eGFR≥60 | 11,961 (50.8%) | 2,839 (42.5%) |  | 10,262 (54.1%) | 4,538 (40.3%) |  |
| eGFR 30-60 | 9,349 (39.7%) | 3,227 (48.3%) |  | 6,965 (36.7%) | 5,611 (49.9%) |  |
| eGFR<30 | 2,245 (9.5%) | 614 (9.2%) |  | 1,754 (9.2%) | 1,105 (9.8%) |  |
| Hemoglobin, g/L | 132 [120.0, 144.0] | 131 [119.0, 143.0] | <0.001 | 134 [122.0, 145.0] | 129 [117.0, 141.0] | <0.001 |
| Potassium, mmol/L | 4.3 [4.0, 4.6] | 4.2 [3.9, 4.6] | <0.001 | 4.3 [4.0, 4.6] | 4.2 [3.9, 4.5] | <0.001 |
| Potessium category |  |  | <0.001 |  |  | <0.001 |
| Hypokalemia <5mmol/L | 614 (3.1%) | 232 (4.4%) |  | 405 (2.5%) | 441 (5.0%) |  |
| Normokalemia 3.5-4.9mmol/L | 17,884 (89.9%) | 4,654 (88.2%) |  | 14,728 (90.3%) | 7,810 (88.1%) |  |
| Hyperkalemia ≥5mmol/L | 1,399 (7.0%) | 388 (7.4%) |  | 1,174 (7.2%) | 613 (6.9%) |  |
| **Comorbidities** |  |  |  |  |  |  |
| Peripheral arterial disease | 2,750 (11.3%) | 871 (12.9%) | 0.001 | 2,197 (11.2%) | 1,424 (12.5%) | 0.001 |
| Stroke/transitory ischemic attack | 4,721 (19.5%) | 1,277 (18.9%) | 0.269 | 3,566 (18.2%) | 2,432 (21.3%) | <0.001 |
| Anemia | 8,118 (36.9%) | 2,536 (39.8%) | <0.001 | 5,929 (33.7%) | 4,725 (43.7%) | <0.001 |
| Cancer past 3 years | 3,702 (15.3%) | 361 (5.3%) | <0.001 | 2,520 (12.8%) | 1,543 (13.5%) | 0.09 |
| Liver disease | 719 (3.0%) | 133 (2.0%) | <0.001 | 452 (2.3%) | 400 (3.5%) | <0.001 |
| Major bleeding | 5,015 (20.7%) | 1,422 (21.0%) | 0.578 | 3,744 (19.1%) | 2,693 (23.6%) | <0.001 |
| Diabetes mellitus | 7,494 (30.9%) | 2,620 (38.7%) | <0.001 | 6,031 (30.8%) | 4,083 (35.8%) | <0.001 |
| Atrial fibrillation | 14,414 (59.5%) | 4,238 (62.6%) | <0.001 | 11,367 (58.0%) | 7,285 (63.9%) | <0.001 |
| Hypertension | 16,065 (66.3%) | 4,384 (64.8%) | 0.022 | 13,023 (66.4%) | 7,426 (65.1%) | 0.022 |
| Chronic obstructive pulmonary disease | 3,470 (14.3%) | 1,268 (18.7%) | <0.001 | 2,677 (13.7%) | 2,061 (18.1%) | <0.001 |
| Ischemic heart disease | 16,000 (66.0%) | 4,683 (69.2%) | <0.001 | 12,766 (65.1%) | 7,917 (69.4%) | <0.001 |
| Revascularized | 9,879 (40.7%) | 2,962 (43.8%) | <0.001 | 8,093 (41.3%) | 4,748 (41.6%) | 0.535 |
| Valvular disease | 6,912 (28.5%) | 2,234 (33.0%) | <0.001 | 5,182 (26.4%) | 3,964 (34.8%) | <0.001 |
| **Treatments** |  |  |  |  |  |  |
| Beta-blockers | 22,149 (91.6%) | 6,275 (92.9%) | <0.001 | 18,048 (92.3%) | 10,376 (91.3%) | 0.002 |
| RASi/ARNi | 21,250 (88.4%) | 6,009 (89.5%) | 0.009 | 17,556 (90.2%) | 9,703 (86.0%) | <0.001 |
| Mineralocorticoid receptor antagonists | 11,426 (47.3%) | 3,611 (53.6%) | <0.001 | 9,365 (48.0%) | 5,672 (50.0%) | <0.001 |
| SGLT2 inhibitor | 607 (33.6%) | 124 (41.2%) | 0.012 | 544 (33.6%) | 187 (38.2%) | 0.066 |
| Diuretics | 19,062 (79.0%) | 6,198 (92.0%) | <0.001 | 14,810 (75.8%) | 10,450 (92.1%) | <0.001 |
| Digoxin | 3,258 (13.5%) | 1,210 (17.9%) | <0.001 | 2,546 (13.0%) | 1,922 (16.9%) | <0.001 |
| Nitrates | 3,878 (16.1%) | 1,072 (15.9%) | 0.763 | 2,951 (15.1%) | 1,999 (17.6%) | <0.001 |
| Anticoagulants | 11,979 (49.6%) | 3,788 (56.2%) | <0.001 | 9,859 (50.4%) | 5,908 (52.0%) | 0.007 |
| Antiplatelets | 10,037 (41.5%) | 2,563 (38.1%) | <0.001 | 8,016 (41.0%) | 4,584 (40.4%) | 0.284 |
| Statins | 13,090 (54.2%) | 3,794 (56.2%) | 0.003 | 11,127 (56.9%) | 5,757 (50.7%) | <0.001 |
| Cardiac resynchronisation therapy | 2,779 (11.6%) | 1,066 (15.8%) | <0.001 | 2,349 (12.1%) | 1,496 (13.2%) | 0.005 |
| Implantable cardioverter-defibrillator | 3,639 (15.1%) | 1,175 (17.4%) | <0.001 | 3,121 (16.0%) | 1,693 (14.9%) | 0.01 |

**Abbreviations:** ARNi, angiotensin receptor–neprilysin inhibitor; b.p.m., beats per minute; BP, blood pressure; eGFR, estimated glomerular filtration rate (calculated by the Chronic Kidney Disease Epidemiology Collaboration formula); HF, heart failure; NT-proBNP, N-terminal pro-B-type natriuretic peptide; NYHA, New York Heart Association; RASi, renin–angiotensin system inhibitor; SGLT2, sodium-glucose co-transporter 2

**Table S11. Baseline characteristics according to eligibility for omecamtiv mecarbil (trial and pragmatic scenario sensitivity analyses considering as ineligible all patients with atrial fibrillation and digoxin use).**

|  | **Trial scenario** | | | **Pragmatic scenario** | | |
| --- | --- | --- | --- | --- | --- | --- |
| **Criterion** | **Ineligible** | **Eligible** | ***P*** | **Ineligible** | **Eligible** | ***P*** |
| *Number of patients (% of study population)* | *25,524 (82.3%)* | *5,491 (17.7%)* |  | *21,664 (69.9%)* | *9,351 (30.1%)* |  |
| **Sociodemographic variables** |  |  |  |  |  |  |
| Female | 6,836 (26.8%) | 1,447 (26.4%) | 0.524 | 5,759 (26.6%) | 2,524 (27.0%) | 0.464 |
| Age, years | 76 [68.0, 83.0] | 76 [68.0, 81.0] | <0.001 | 75 [67.0, 81.0] | 78 [70.0, 84.0] | <0.001 |
| Age ≥75 years | 13,965 (54.7%) | 3,039 (55.3%) | 0.402 | 11,265 (52.0%) | 5,739 (61.4%) | <0.001 |
| Index year |  |  | <0.001 |  |  | <0.001 |
| 2000-2008 | 4,003 (15.7%) | 1,002 (18.2%) |  | 3,337 (15.4%) | 1,668 (17.8%) |  |
| 2009-2015 | 9,262 (36.3%) | 2,422 (44.1%) |  | 7,473 (34.5%) | 4,211 (45.0%) |  |
| 2016-2021 | 12,259 (48.0%) | 2,067 (37.6%) |  | 10,854 (50.1%) | 3,472 (37.1%) |  |
| Income level <median (by index year) | 12,632 (49.5%) | 2,848 (51.9%) | 0.001 | 10,512 (48.6%) | 4,968 (53.2%) | <0.001 |
| Education: compulsory school only (vs. secondary school/university) | 10,821 (43.2%) | 2,445 (45.6%) | 0.001 | 8,974 (42.2%) | 4,292 (46.9%) | <0.001 |
| Single living | 12,129 (47.6%) | 2,504 (45.6%) | 0.011 | 10,001 (46.2%) | 4,632 (49.6%) | <0.001 |
| Children | 21,282 (83.4%) | 4,509 (82.1%) | 0.024 | 18,083 (83.5%) | 7,708 (82.4%) | 0.026 |
| **Health organizational variables** |  |  |  |  |  |  |
| Caregiver: in-patient (vs. out-patient) | 7,654 (30.0%) | 2,442 (44.5%) | <0.001 | 5,306 (24.5%) | 4,790 (51.2%) | <0.001 |
| Planned follow-up: specialty care (vs. primary care/other) | 16,885 (69.4%) | 3,791 (72.3%) | <0.001 | 14,912 (71.9%) | 5,764 (65.2%) | <0.001 |
| Referral to follow-up in a nurse-led HF unit | 14,290 (59.4%) | 2,931 (56.8%) | 0.001 | 12,718 (62.1%) | 4,503 (51.7%) | <0.001 |
| **Clinical variables** |  |  |  |  |  |  |
| Ejection fraction <30% | 11,448 (44.9%) | 3,357 (61.1%) | <0.001 | 9,335 (43.1%) | 5,470 (58.5%) | <0.001 |
| NYHA class |  |  | <0.001 |  |  | <0.001 |
| I | 1,952 (9.6%) | 0 (0.0%) |  | 1,952 (11.1%) | 0 (0.0%) |  |
| II | 8,753 (43.2%) | 1,635 (38.0%) |  | 7,870 (44.7%) | 2,518 (36.3%) |  |
| III | 8,681 (42.9%) | 2,357 (54.7%) |  | 7,159 (40.6%) | 3,879 (55.9%) |  |
| IV | 872 (4.3%) | 314 (7.3%) |  | 643 (3.6%) | 543 (7.8%) |  |
| HF duration ≥6 months | 20,645 (82.2%) | 4,407 (81.1%) | 0.07 | 17,506 (82.1%) | 7,546 (81.6%) | 0.275 |
| Mean arterial pressure, mmHg | 88 [80.0, 96.7] | 83.3 [76.7, 91.7] | <0.001 | 89 [80.0, 98.0] | 83.3 [76.7, 91.7] | <0.001 |
| Mean arterial pressure <90 mmHg | 13,117 (53.2%) | 3,647 (68.1%) | <0.001 | 10,583 (50.7%) | 6,181 (67.6%) | <0.001 |
| Heart rate, b.p.m. | 70 [62.0, 80.0] | 72 [64.0, 80.0] | <0.001 | 70 [62.0, 80.0] | 72 [64.0, 81.0] | <0.001 |
| Heart rate ≥70 b.p.m. | 13,560 (56.3%) | 3,093 (59.5%) | <0.001 | 11,201 (55.0%) | 5,452 (61.3%) | <0.001 |
| Body mass index, kg/m² | 26 [23.0, 29.7] | 26.1 [23.1, 30.0] | 0.174 | 26.3 [23.3, 30.0] | 25.5 [22.6, 29.3] | <0.001 |
| Body mass index category |  |  | 0.02 |  |  | <0.001 |
| Normal/overweight | 16,425 (73.3%) | 3,636 (71.4%) |  | 13,585 (72.2%) | 6,476 (74.6%) |  |
| Underweight | 673 (3.0%) | 163 (3.2%) |  | 501 (2.7%) | 335 (3.9%) |  |
| Obese | 5,307 (23.7%) | 1,295 (25.4%) |  | 4,728 (25.1%) | 1,874 (21.6%) |  |
| HF hospitalization past 6 months | 11,636 (45.6%) | 4,812 (87.6%) | <0.001 | 8,089 (37.3%) | 8,359 (89.4%) | <0.001 |
| HF hospitalization past 12 months | 12,953 (50.7%) | 5,491 (100.0%) | <0.001 | 9,093 (42.0%) | 9,351 (100.0%) | <0.001 |
| **Laboratory variables** |  |  |  |  |  |  |
| NT-proBNP, pg/L | 2,183 [859.0, 5562.0] | 4,201 [2040.0, 8620.0] | <0.001 | 1,840 [735.0, 4558.0] | 4,815 [2276.0, 10100.0] | <0.001 |
| NT-proBNP tertile (by EF and atrial fibrillation) |  |  | <0.001 |  |  | <0.001 |
| NT-proBNP lowest tertile | 5,486 (37.6%) | 400 (13.0%) |  | 5,294 (42.4%) | 592 (11.4%) |  |
| NT-proBNP middle tertile | 4,698 (32.2%) | 1,188 (38.7%) |  | 4,045 (32.4%) | 1,841 (35.5%) |  |
| NT-proBNP highest tertile | 4,405 (30.2%) | 1,483 (48.3%) |  | 3,140 (25.2%) | 2,748 (53.0%) |  |
| eGFR, mL/min/1.73m² | 60.2 [43.1, 80.5] | 55.3 [40.4, 74.2] | <0.001 | 62.1 [44.7, 82.0] | 53.5 [39.0, 72.0] | <0.001 |
| eGFR category |  |  | <0.001 |  |  | <0.001 |
| eGFR≥60 | 12,481 (50.3%) | 2,319 (42.9%) |  | 11,126 (52.9%) | 3,674 (39.8%) |  |
| eGFR 30-60 | 9,998 (40.3%) | 2,578 (47.6%) |  | 7,995 (38.0%) | 4,581 (49.7%) |  |
| eGFR<30 | 2,345 (9.4%) | 514 (9.5%) |  | 1,892 (9.0%) | 967 (10.5%) |  |
| Hemoglobin, g/L | 132 [120.0, 144.0] | 131 [119.0, 142.0] | <0.001 | 133 [121.0, 145.0] | 129 [117.0, 141.0] | <0.001 |
| Potassium, mmol/L | 4.3 [4.0, 4.6] | 4.2 [3.9, 4.5] | <0.001 | 4.3 [4.0, 4.6] | 4.2 [3.9, 4.5] | <0.001 |
| Potessium category |  |  | 0.002 |  |  | <0.001 |
| Hypokalemia <5mmol/L | 663 (3.2%) | 183 (4.2%) |  | 485 (2.7%) | 361 (4.9%) |  |
| Normokalemia 3.5-4.9mmol/L | 18,701 (89.8%) | 3,837 (88.4%) |  | 16,033 (90.1%) | 6,505 (88.1%) |  |
| Hyperkalemia ≥5mmol/L | 1,465 (7.0%) | 322 (7.4%) |  | 1,270 (7.1%) | 517 (7.0%) |  |
| **Comorbidities** |  |  |  |  |  |  |
| Peripheral arterial disease | 2,888 (11.3%) | 733 (13.3%) | <0.001 | 2,423 (11.2%) | 1,198 (12.8%) | <0.001 |
| Stroke/transitory ischemic attack | 4,961 (19.4%) | 1,037 (18.9%) | 0.358 | 4,007 (18.5%) | 1,991 (21.3%) | <0.001 |
| Anemia | 8,606 (37.0%) | 2,048 (39.9%) | <0.001 | 6,748 (34.5%) | 3,906 (44.2%) | <0.001 |
| Cancer past 3 years | 3,780 (14.8%) | 283 (5.2%) | <0.001 | 2,802 (12.9%) | 1,261 (13.5%) | 0.193 |
| Liver disease | 748 (2.9%) | 104 (1.9%) | <0.001 | 543 (2.5%) | 309 (3.3%) | <0.001 |
| Major bleeding | 5,258 (20.6%) | 1,179 (21.5%) | 0.154 | 4,212 (19.4%) | 2,225 (23.8%) | <0.001 |
| Diabetes mellitus | 7,957 (31.2%) | 2,157 (39.3%) | <0.001 | 6,729 (31.1%) | 3,385 (36.2%) | <0.001 |
| Atrial fibrillation | 15,605 (61.1%) | 3,047 (55.5%) | <0.001 | 13,274 (61.3%) | 5,378 (57.5%) | <0.001 |
| Hypertension | 16,832 (65.9%) | 3,617 (65.9%) | 0.928 | 14,252 (65.8%) | 6,197 (66.3%) | 0.416 |
| Chronic obstructive pulmonary disease | 3,712 (14.5%) | 1,026 (18.7%) | <0.001 | 3,087 (14.2%) | 1,651 (17.7%) | <0.001 |
| Ischemic heart disease | 16,835 (66.0%) | 3,848 (70.1%) | <0.001 | 14,087 (65.0%) | 6,596 (70.5%) | <0.001 |
| Revascularized | 10,349 (40.5%) | 2,492 (45.4%) | <0.001 | 8,810 (40.7%) | 4,031 (43.1%) | <0.001 |
| Valvular disease | 7,381 (28.9%) | 1,765 (32.1%) | <0.001 | 5,969 (27.6%) | 3,177 (34.0%) | <0.001 |
| **Treatments** |  |  |  |  |  |  |
| Beta-blockers | 23,332 (91.7%) | 5,092 (93.0%) | 0.001 | 19,940 (92.3%) | 8,484 (91.0%) | <0.001 |
| RASi/ARNi | 22,384 (88.4%) | 4,875 (89.5%) | 0.021 | 19,333 (89.9%) | 7,926 (85.7%) | <0.001 |
| Mineralocorticoid receptor antagonists | 12,139 (47.8%) | 2,898 (53.1%) | <0.001 | 10,484 (48.6%) | 4,553 (49.0%) | 0.538 |
| SGLT2 inhibitor | 617 (33.6%) | 114 (41.9%) | 0.009 | 566 (33.7%) | 165 (38.6%) | 0.067 |
| Diuretics | 20,267 (79.7%) | 4,993 (91.4%) | <0.001 | 16,736 (77.5%) | 8,524 (91.7%) | <0.001 |
| Digoxin | 4,339 (17.1%) | 129 (2.4%) | <0.001 | 4,287 (19.9%) | 181 (1.9%) | <0.001 |
| Nitrates | 4,072 (16.0%) | 878 (16.1%) | 0.956 | 3,291 (15.2%) | 1,659 (17.8%) | <0.001 |
| Anticoagulants | 12,992 (51.1%) | 2,775 (50.8%) | 0.691 | 11,349 (52.6%) | 4,418 (47.5%) | <0.001 |
| Antiplatelets | 10,324 (40.6%) | 2,276 (41.7%) | 0.145 | 8,573 (39.7%) | 4,027 (43.3%) | <0.001 |
| Statins | 13,728 (54.0%) | 3,156 (57.7%) | <0.001 | 12,053 (55.8%) | 4,831 (51.8%) | <0.001 |
| Cardiac resynchronisation therapy | 2,994 (11.8%) | 851 (15.6%) | <0.001 | 2,645 (12.3%) | 1,200 (12.9%) | 0.148 |
| Implantable cardioverter-defibrillator | 3,843 (15.2%) | 971 (17.8%) | <0.001 | 3,414 (15.9%) | 1,400 (15.1%) | 0.07 |

**Abbreviations:** ARNi, angiotensin receptor–neprilysin inhibitor; b.p.m., beats per minute; eGFR, estimated glomerular filtration rate (calculated by the Chronic Kidney Disease Epidemiology Collaboration formula); HF, heart failure; NT-proBNP, N-terminal pro-B-type natriuretic peptide; NYHA, New York Heart Association; RASi, renin–angiotensin system inhibitor; SGLT2, sodium-glucose co-transporter 2

**Table S12. Baseline characteristics according to eligibility for omecamtiv mecarbil (trial scenario sensitivity analysis considering a strict interpretation of the optimal-medical-therapy criterion).**

|  | **Trial scenario: OMT sensitivity** | | |
| --- | --- | --- | --- |
| **Criterion** | **Ineligible** | **Eligible** | ***P*** |
| *Number of patients (% of study population)* | *27,962 (90.2%)* | *3,053 (9.8%)* |  |
| **Sociodemographic variables** |  |  |  |
| Female | 7,473 (26.7%) | 810 (26.5%) | 0.835 |
| Age, years | 76 [68.0, 83.0] | 74 [67.0, 79.0] | <0.001 |
| Age ≥75 years | 15,538 (55.6%) | 1,466 (48.0%) | <0.001 |
| Index year |  |  | 0.419 |
| 2000-2008 | 4,499 (16.1%) | 506 (16.6%) |  |
| 2009-2015 | 10,513 (37.6%) | 1,171 (38.4%) |  |
| 2016-2021 | 12,950 (46.3%) | 1,376 (45.1%) |  |
| Income level <median (by index year) | 13,979 (50.0%) | 1,501 (49.2%) | 0.415 |
| Education: compulsory school only (vs. secondary school/university) | 11,988 (43.7%) | 1,278 (42.8%) | 0.338 |
| Single living | 13,261 (47.5%) | 1,372 (45.0%) | 0.01 |
| Children | 23,282 (83.3%) | 2,509 (82.2%) | 0.136 |
| **Health organizational variables** |  |  |  |
| Caregiver: in-patient (vs. out-patient) | 8,964 (32.1%) | 1,132 (37.1%) | <0.001 |
| Planned follow-up: specialty care (vs. primary care/other) | 18,341 (68.9%) | 2,335 (79.3%) | <0.001 |
| Referral to follow-up in a nurse-led HF unit | 15,382 (58.5%) | 1,839 (63.7%) | <0.001 |
| **Clinical variables** |  |  |  |
| Ejection fraction <30% | 12,869 (46.0%) | 1,936 (63.4%) | <0.001 |
| NYHA class |  |  | <0.001 |
| I | 1,952 (8.8%) | 0 (0.0%) |  |
| II | 9,367 (42.4%) | 1,021 (41.0%) |  |
| III | 9,693 (43.9%) | 1,345 (54.0%) |  |
| IV | 1,060 (4.8%) | 126 (5.1%) |  |
| HF duration ≥6 months | 22,592 (82.0%) | 2,460 (81.4%) | 0.411 |
| Mean arterial pressure, mmHg | 87 [80.0, 96.7] | 83.3 [76.7, 90.0] | <0.001 |
| Mean arterial pressure <90 mmHg | 14,683 (54.3%) | 2,081 (69.9%) | <0.001 |
| Heart rate, b.p.m. | 70 [62.0, 80.0] | 71 [64.0, 80.0] | 0.007 |
| Heart rate ≥70 b.p.m. | 14,943 (56.6%) | 1,710 (59.2%) | 0.008 |
| Body mass index, kg/m² | 26 [23.0, 29.7] | 26.5 [23.5, 30.2] | <0.001 |
| Body mass index category |  |  | <0.001 |
| Normal/overweight | 18,059 (73.3%) | 2,002 (70.3%) |  |
| Underweight | 761 (3.1%) | 75 (2.6%) |  |
| Obese | 5,831 (23.7%) | 771 (27.1%) |  |
| HF hospitalization past 6 months | 13,824 (49.4%) | 2,624 (85.9%) | <0.001 |
| HF hospitalization past 12 months | 15,391 (55.0%) | 3,053 (100.0%) | <0.001 |
| **Laboratory variables** |  |  |  |
| NT-proBNP, pg/L | 2,376 [920.0, 6016.0] | 3,449 [1846.5, 6828.5] | <0.001 |
| NT-proBNP tertile (by EF and atrial fibrillation) |  |  | <0.001 |
| NT-proBNP lowest tertile | 5,568 (35.3%) | 318 (16.8%) |  |
| NT-proBNP middle tertile | 5,084 (32.2%) | 802 (42.4%) |  |
| NT-proBNP highest tertile | 5,117 (32.4%) | 771 (40.8%) |  |
| eGFR, mL/min/1.73m² | 59 [42.1, 79.3] | 61.3 [46.5, 78.7] | <0.001 |
| eGFR category |  |  | <0.001 |
| eGFR≥60 | 13,236 (48.6%) | 1,564 (52.2%) |  |
| eGFR 30-60 | 11,283 (41.4%) | 1,293 (43.2%) |  |
| eGFR<30 | 2,721 (10.0%) | 138 (4.6%) |  |
| Hemoglobin, g/L | 132 [120.0, 144.0] | 133 [121.0, 144.0] | 0.001 |
| Potassium, mmol/L | 4.3 [4.0, 4.6] | 4.3 [4.0, 4.6] | 0.162 |
| Potessium category |  |  | 0.065 |
| Hypokalemia <5mmol/L | 778 (3.4%) | 68 (2.8%) |  |
| Normokalemia 3.5-4.9mmol/L | 20,332 (89.6%) | 2,206 (89.3%) |  |
| Hyperkalemia ≥5mmol/L | 1,592 (7.0%) | 195 (7.9%) |  |
| **Comorbidities** |  |  |  |
| Peripheral arterial disease | 3,257 (11.6%) | 364 (11.9%) | 0.675 |
| Stroke/transitory ischemic attack | 5,522 (19.7%) | 476 (15.6%) | <0.001 |
| Anemia | 9,667 (37.8%) | 987 (35.2%) | 0.007 |
| Cancer past 3 years | 3,902 (14.0%) | 161 (5.3%) | <0.001 |
| Liver disease | 793 (2.8%) | 59 (1.9%) | 0.004 |
| Major bleeding | 5,865 (21.0%) | 572 (18.7%) | 0.004 |
| Diabetes mellitus | 8,948 (32.0%) | 1,166 (38.2%) | <0.001 |
| Atrial fibrillation | 16,802 (60.1%) | 1,850 (60.6%) | 0.6 |
| Hypertension | 18,420 (65.9%) | 2,029 (66.5%) | 0.531 |
| Chronic obstructive pulmonary disease | 4,208 (15.0%) | 530 (17.4%) | 0.001 |
| Ischemic heart disease | 18,656 (66.7%) | 2,027 (66.4%) | 0.732 |
| Revascularized | 11,487 (41.1%) | 1,354 (44.3%) | 0.001 |
| Valvular disease | 8,192 (29.3%) | 954 (31.2%) | 0.026 |
| **Treatments** |  |  |  |
| Beta-blockers | 25,379 (91.0%) | 3,045 (100.0%) | <0.001 |
| RASi/ARNi | 24,230 (87.4%) | 3,029 (100.0%) | <0.001 |
| Mineralocorticoid receptor antagonists | 11,997 (43.1%) | 3,040 (100.0%) | <0.001 |
| SGLT2 inhibitor | 636 (33.4%) | 95 (47.0%) | <0.001 |
| Diuretics | 22,486 (80.8%) | 2,774 (91.4%) | <0.001 |
| Digoxin | 3,889 (14.0%) | 579 (19.0%) | <0.001 |
| Nitrates | 4,530 (16.3%) | 420 (13.8%) | 0.001 |
| Anticoagulants | 13,969 (50.1%) | 1,798 (59.2%) | <0.001 |
| Antiplatelets | 11,508 (41.3%) | 1,092 (36.0%) | <0.001 |
| Statins | 15,097 (54.2%) | 1,787 (58.7%) | <0.001 |
| Cardiac resynchronisation therapy | 3,295 (11.9%) | 550 (18.1%) | <0.001 |
| Implantable cardioverter-defibrillator | 4,157 (15.0%) | 657 (21.6%) | <0.001 |

**Abbreviations:** ARNi, angiotensin receptor–neprilysin inhibitor; b.p.m., beats per minute; eGFR, estimated glomerular filtration rate (calculated by the Chronic Kidney Disease Epidemiology Collaboration formula); HF, heart failure; NT-proBNP, N-terminal pro-B-type natriuretic peptide; NYHA, New York Heart Association; OMT, optimal medical therapy; RASi, renin–angiotensin system inhibitor; SGLT2, sodium-glucose co-transporter 2

**Table S13. Baseline characteristics according to eligibility for omecamtiv mecarbil (trial and pragmatic scenario sensitivity analyses considering as ineligible all patients with atrial fibrillation).**

|  | **Trial scenario** | | | **Pragmatic scenario** | | |
| --- | --- | --- | --- | --- | --- | --- |
| **Criterion** | **Ineligible** | **Eligible** | ***P*** | **Ineligible** | **Eligible** | ***P*** |
| *Number of patients (% of study population)* | *28,571 (92.1%)* | *2,444 (7.9%)* |  | *27,042 (87.2%)* | *3,973 (12.8%)* |  |
| **Sociodemographic variables** |  |  |  |  |  |  |
| Female | 7,527 (26.3%) | 756 (30.9%) | <0.001 | 7,029 (26.0%) | 1,254 (31.6%) | <0.001 |
| Age, years | 76 [68.0, 82.0] | 73 [64.0, 79.0] | <0.001 | 76 [68.0, 82.0] | 75 [65.0, 82.0] | <0.001 |
| Age ≥75 years | 15,915 (55.7%) | 1,089 (44.6%) | <0.001 | 15,007 (55.5%) | 1,997 (50.3%) | <0.001 |
| Index year |  |  | <0.001 |  |  | <0.001 |
| 2000-2008 | 4,504 (15.8%) | 501 (20.5%) |  | 4,207 (15.6%) | 798 (20.1%) |  |
| 2009-2015 | 10,632 (37.2%) | 1,052 (43.0%) |  | 9,958 (36.8%) | 1,726 (43.4%) |  |
| 2016-2021 | 13,435 (47.0%) | 891 (36.5%) |  | 12,877 (47.6%) | 1,449 (36.5%) |  |
| Income level <median (by index year) | 14,183 (49.7%) | 1,297 (53.1%) | 0.001 | 13,291 (49.2%) | 2,189 (55.1%) | <0.001 |
| Education: compulsory school only (vs. secondary school/university) | 12,210 (43.6%) | 1,056 (44.4%) | 0.446 | 11,507 (43.4%) | 1,759 (45.4%) | 0.022 |
| Single living | 13,459 (47.1%) | 1,174 (48.1%) | 0.386 | 12,570 (46.5%) | 2,063 (52.0%) | <0.001 |
| Children | 23,832 (83.4%) | 1,959 (80.2%) | <0.001 | 22,592 (83.5%) | 3,199 (80.5%) | <0.001 |
| **Health organizational variables** |  |  |  |  |  |  |
| Caregiver: in-patient (vs. out-patient) | 9,126 (31.9%) | 970 (39.7%) | <0.001 | 8,246 (30.5%) | 1,850 (46.6%) | <0.001 |
| Planned follow-up: specialty care (vs. primary care/other) | 18,946 (69.5%) | 1,730 (74.3%) | <0.001 | 18,099 (70.1%) | 2,577 (68.6%) | 0.06 |
| Referral to follow-up in a nurse-led HF unit | 15,914 (59.2%) | 1,307 (56.9%) | 0.041 | 15,263 (59.9%) | 1,958 (52.9%) | <0.001 |
| **Clinical variables** |  |  |  |  |  |  |
| Ejection fraction <30% | 13,214 (46.2%) | 1,591 (65.1%) | <0.001 | 12,306 (45.5%) | 2,499 (62.9%) | <0.001 |
| NYHA class |  |  | <0.001 |  |  | <0.001 |
| I | 1,952 (8.6%) | 0 (0.0%) |  | 1,952 (9.1%) | 0 (0.0%) |  |
| II | 9,510 (42.1%) | 878 (44.7%) |  | 9,103 (42.3%) | 1,285 (42.5%) |  |
| III | 10,070 (44.6%) | 968 (49.3%) |  | 9,491 (44.1%) | 1,547 (51.1%) |  |
| IV | 1,068 (4.7%) | 118 (6.0%) |  | 992 (4.6%) | 194 (6.4%) |  |
| HF duration ≥6 months | 23,186 (82.4%) | 1,866 (77.2%) | <0.001 | 22,000 (82.6%) | 3,052 (77.7%) | <0.001 |
| Mean arterial pressure, mmHg | 86.7 [79.7, 96.7] | 83.3 [76.7, 91.7] | <0.001 | 87.3 [80.0, 96.7] | 83.3 [76.7, 91.7] | <0.001 |
| Mean arterial pressure <90 mmHg | 15,140 (54.8%) | 1,624 (68.3%) | <0.001 | 14,147 (54.2%) | 2,617 (67.5%) | <0.001 |
| Heart rate, b.p.m. | 70 [63.0, 80.0] | 70 [62.0, 78.0] | <0.001 | 70 [63.0, 80.0] | 70 [62.0, 79.0] | <0.001 |
| Heart rate ≥70 b.p.m. | 15,483 (57.4%) | 1,170 (50.8%) | <0.001 | 14,645 (57.4%) | 2,008 (53.2%) | <0.001 |
| Body mass index, kg/m² | 26 [23.1, 29.7] | 26 [22.9, 29.9] | 0.682 | 26.1 [23.1, 29.8] | 25.5 [22.5, 29.4] | <0.001 |
| Body mass index category |  |  | 0.065 |  |  | <0.001 |
| Normal/overweight | 18,459 (73.1%) | 1,602 (71.4%) |  | 17,381 (72.9%) | 2,680 (73.4%) |  |
| Underweight | 752 (3.0%) | 84 (3.7%) |  | 668 (2.8%) | 168 (4.6%) |  |
| Obese | 6,045 (23.9%) | 557 (24.8%) |  | 5,797 (24.3%) | 805 (22.0%) |  |
| HF hospitalization past 6 months | 14,383 (50.3%) | 2,065 (84.5%) | <0.001 | 12,994 (48.1%) | 3,454 (86.9%) | <0.001 |
| HF hospitalization past 12 months | 16,000 (56.0%) | 2,444 (100.0%) | <0.001 | 14,471 (53.5%) | 3,973 (100.0%) | <0.001 |
| **Laboratory variables** |  |  |  |  |  |  |
| NT-proBNP, pg/L | 2,475 [974.0, 6087.5] | 3,000 [1227.0, 6780.0] | <0.001 | 2,384 [942.0, 5825.0] | 3,590 [1388.0, 8713.5] | <0.001 |
| NT-proBNP tertile (by EF and atrial fibrillation) |  |  | <0.001 |  |  | <0.001 |
| NT-proBNP lowest tertile | 5,716 (34.9%) | 170 (13.1%) |  | 5,649 (36.3%) | 237 (11.2%) |  |
| NT-proBNP middle tertile | 5,403 (33.0%) | 483 (37.4%) |  | 5,169 (33.2%) | 717 (34.0%) |  |
| NT-proBNP highest tertile | 5,248 (32.1%) | 640 (49.5%) |  | 4,735 (30.4%) | 1,153 (54.7%) |  |
| eGFR, mL/min/1.73m² | 59.1 [42.5, 79.1] | 61.2 [44.4, 80.7] | <0.001 | 59.3 [42.6, 79.3] | 59.6 [43.0, 79.4] | 0.127 |
| eGFR category |  |  | <0.001 |  |  | 0.005 |
| eGFR≥60 | 13,549 (48.7%) | 1,251 (52.0%) |  | 12,871 (48.9%) | 1,929 (49.4%) |  |
| eGFR 30-60 | 11,592 (41.7%) | 984 (40.9%) |  | 10,912 (41.4%) | 1,664 (42.6%) |  |
| eGFR<30 | 2,690 (9.7%) | 169 (7.0%) |  | 2,545 (9.7%) | 314 (8.0%) |  |
| Hemoglobin, g/L | 132 [120.0, 144.0] | 132 [120.0, 144.0] | 0.971 | 132 [120.0, 144.0] | 130 [118.0, 142.0] | <0.001 |
| Potassium, mmol/L | 4.3 [4.0, 4.6] | 4.3 [4.0, 4.6] | 0.495 | 4.3 [4.0, 4.6] | 4.2 [3.9, 4.6] | 0.003 |
| Potessium category |  |  | 0.513 |  |  | 0.009 |
| Hypokalemia <5mmol/L | 787 (3.4%) | 59 (3.1%) |  | 714 (3.2%) | 132 (4.3%) |  |
| Normokalemia 3.5-4.9mmol/L | 20,833 (89.6%) | 1,705 (89.3%) |  | 19,797 (89.7%) | 2,741 (88.4%) |  |
| Hyperkalemia ≥5mmol/L | 1,641 (7.1%) | 146 (7.6%) |  | 1,558 (7.1%) | 229 (7.4%) |  |
| **Comorbidities** |  |  |  |  |  |  |
| Peripheral arterial disease | 3,312 (11.6%) | 309 (12.6%) | 0.128 | 3,150 (11.6%) | 471 (11.9%) | 0.725 |
| Stroke/transitory ischemic attack | 5,643 (19.8%) | 355 (14.5%) | <0.001 | 5,332 (19.7%) | 666 (16.8%) | <0.001 |
| Anemia | 9,838 (37.7%) | 816 (36.0%) | 0.121 | 9,144 (37.1%) | 1,510 (40.5%) | <0.001 |
| Cancer past 3 years | 3,966 (13.9%) | 97 (4.0%) | <0.001 | 3,612 (13.4%) | 451 (11.4%) | 0.001 |
| Liver disease | 800 (2.8%) | 52 (2.1%) | 0.059 | 708 (2.6%) | 144 (3.6%) | <0.001 |
| Major bleeding | 6,015 (21.1%) | 422 (17.3%) | <0.001 | 5,672 (21.0%) | 765 (19.3%) | 0.013 |
| Diabetes mellitus | 9,182 (32.1%) | 932 (38.1%) | <0.001 | 8,670 (32.1%) | 1,444 (36.3%) | <0.001 |
| Atrial fibrillation | 18,652 (65.3%) | 0 (0.0%) | <0.001 | 18,652 (69.0%) | 0 (0.0%) | <0.001 |
| Hypertension | 18,966 (66.4%) | 1,483 (60.7%) | <0.001 | 17,975 (66.5%) | 2,474 (62.3%) | <0.001 |
| Chronic obstructive pulmonary disease | 4,295 (15.0%) | 443 (18.1%) | <0.001 | 4,027 (14.9%) | 711 (17.9%) | <0.001 |
| Ischemic heart disease | 19,011 (66.5%) | 1,672 (68.4%) | 0.062 | 17,930 (66.3%) | 2,753 (69.3%) | <0.001 |
| Revascularized | 11,760 (41.2%) | 1,081 (44.2%) | 0.003 | 11,154 (41.2%) | 1,687 (42.5%) | 0.152 |
| Valvular disease | 8,504 (29.8%) | 642 (26.3%) | <0.001 | 8,005 (29.6%) | 1,141 (28.7%) | 0.262 |
| **Treatments** |  |  |  |  |  |  |
| Beta-blockers | 26,160 (91.8%) | 2,264 (92.9%) | 0.053 | 24,822 (92.0%) | 3,602 (91.0%) | 0.027 |
| RASi/ARNi | 25,020 (88.3%) | 2,239 (92.4%) | <0.001 | 23,754 (88.5%) | 3,505 (89.3%) | 0.173 |
| Mineralocorticoid receptor antagonists | 13,717 (48.2%) | 1,320 (54.3%) | <0.001 | 13,059 (48.5%) | 1,978 (50.2%) | 0.056 |
| SGLT2 inhibitor | 675 (34.0%) | 56 (45.5%) | 0.012 | 652 (33.9%) | 79 (42.5%) | 0.024 |
| Diuretics | 23,110 (81.2%) | 2,150 (88.4%) | <0.001 | 21,768 (80.8%) | 3,492 (88.4%) | <0.001 |
| Digoxin | 4,339 (15.2%) | 129 (5.3%) | <0.001 | 4,287 (15.9%) | 181 (4.6%) | <0.001 |
| Nitrates | 4,559 (16.0%) | 391 (16.1%) | 0.959 | 4,259 (15.8%) | 691 (17.5%) | 0.007 |
| Anticoagulants | 15,312 (53.8%) | 455 (18.7%) | <0.001 | 15,082 (56.0%) | 685 (17.3%) | <0.001 |
| Antiplatelets | 11,057 (38.9%) | 1,543 (63.4%) | <0.001 | 10,089 (37.5%) | 2,511 (63.5%) | <0.001 |
| Statins | 15,447 (54.2%) | 1,437 (59.0%) | <0.001 | 14,736 (54.7%) | 2,148 (54.3%) | 0.635 |
| Cardiac resynchronisation therapy | 3,512 (12.4%) | 333 (13.7%) | 0.065 | 3,408 (12.7%) | 437 (11.1%) | 0.005 |
| Implantable cardioverter-defibrillator | 4,384 (15.5%) | 430 (17.7%) | 0.004 | 4,212 (15.7%) | 602 (15.3%) | 0.508 |

**Abbreviations:** ARNi, angiotensin receptor–neprilysin inhibitor; b.p.m., beats per minute; eGFR, estimated glomerular filtration rate (calculated by the Chronic Kidney Disease Epidemiology Collaboration formula); HF, heart failure; NT-proBNP, N-terminal pro-B-type natriuretic peptide; NYHA, New York Heart Association; RASi, renin–angiotensin system inhibitor; SGLT2, sodium-glucose co-transporter 2

**Table S14. Outcomes eligible and ineligible patients in the trial and pragmatic scenario in patients with ejection fraction <30%.**

|  | **Events per 100 patient-years (95% CI)** | |  |  |
| --- | --- | --- | --- | --- |
| **Event** | **Ineligible** | **Eligible** | ***IRR*** | ***P*** |
| **Trial scenario** | |  |  |  |
| All-cause death | 21.7 (21.2-22.2) | 24.8 (23.9-25.7) | 1.14 (1.09-1.19) | <0.001 |
| CV death | 14.5 (14.1-14.9) | 17.9 (17.2-18.7) | 1.24 (1.18-1.31) | <0.001 |
| Non-CV death | 7.2 (6.9-7.5) | 6.8 (6.4-7.3) | 0.95 (0.87-1.03) | 0.208 |
| All-cause hospitalization | 59.8 (58.5-61.1) | 72.5 (70.0-75.1) | 1.21 (1.16-1.26) | <0.001 |
| CV hospitalization | 31.2 (30.4-32.0) | 42.9 (41.3-44.6) | 1.38 (1.31-1.44) | <0.001 |
| HF hospitalization | 19.3 (18.7-19.9) | 29.7 (28.4-30.9) | 1.54 (1.46-1.62) | <0.001 |
| Non-CV hospitalization | 30.9 (30.1-31.7) | 32.6 (31.2-34.0) | 1.06 (1.01-1.11) | 0.03 |
| Stroke/TIA hospitalization | 2.5 (2.3-2.7) | 2.3 (2.1-2.6) | 0.93 (0.81-1.07) | 0.341 |
| **Pragmatic scenario** | |  |  |  |
| All-cause death | 18.6 (18.1-19.1) | 28.4 (27.6-29.2) | 1.53 (1.47-1.59) | <0.001 |
| CV death | 12.3 (11.9-12.7) | 20.1 (19.5-20.8) | 1.63 (1.56-1.72) | <0.001 |
| Non-CV death | 6.3 (6.0-6.6) | 8.3 (7.9-8.7) | 1.32 (1.23-1.42) | <0.001 |
| All-cause hospitalization | 51.8 (50.5-53.2) | 83.6 (81.3-85.9) | 1.61 (1.55-1.67) | <0.001 |
| CV hospitalization | 27.4 (26.6-28.2) | 45.8 (44.4-47.2) | 1.67 (1.60-1.75) | <0.001 |
| HF hospitalization | 16.2 (15.7-16.8) | 31.8 (30.7-32.9) | 1.96 (1.86-2.05) | <0.001 |
| Non-CV hospitalization | 27.5 (26.7-28.4) | 37.7 (36.4-38.9) | 1.37 (1.31-1.43) | <0.001 |
| Stroke/TIA hospitalization | 2.4 (2.2-2.6) | 2.5 (2.3-2.8) | 1.05 (0.92-1.19) | 0.462 |
| **Trial scenario, OMT sensitivity** | |  |  |  |
| All-cause death | 22.8 (22.4-23.3) | 20.4 (19.3-21.6) | 0.90 (0.84-0.95) | <0.001 |
| CV death | 15.6 (15.2-16.0) | 14.3 (13.4-15.3) | 0.92 (0.85-0.99) | 0.02 |
| Non-CV death | 7.2 (7.0-7.5) | 6.1 (5.5-6.8) | 0.85 (0.76-0.94) | 0.002 |
| All-cause hospitalization | 62.5 (61.2-63.7) | 66.0 (62.7-69.4) | 1.06 (1.00-1.12) | 0.046 |
| CV hospitalization | 33.0 (32.2-33.7) | 41.3 (39.0-43.6) | 1.25 (1.18-1.33) | <0.001 |
| HF hospitalization | 20.8 (20.3-21.3) | 28.9 (27.2-30.7) | 1.39 (1.30-1.48) | <0.001 |
| Non-CV hospitalization | 31.6 (30.9-32.4) | 29.5 (27.7-31.4) | 0.93 (0.87-1.00) | 0.038 |
| Stroke/TIA hospitalization | 2.5 (2.4-2.7) | 2.1 (1.7-2.5) | 0.82 (0.67-0.99) | 0.037 |
| **Trial scenario, BP sensitivity** | |  |  |  |
| All-cause death | 21.4 (20.9-21.9) | 25.3 (24.4-26.2) | 1.18 (1.13-1.23) | <0.001 |
| CV death | 14.3 (13.8-14.7) | 18.4 (17.7-19.2) | 1.29 (1.23-1.36) | <0.001 |
| Non-CV death | 7.2 (6.9-7.5) | 6.9 (6.4-7.4) | 0.96 (0.89-1.04) | 0.323 |
| All-cause hospitalization | 59.2 (57.9-60.5) | 74.1 (71.6-76.6) | 1.25 (1.20-1.30) | <0.001 |
| CV hospitalization | 30.8 (30.0-31.6) | 43.9 (42.2-45.5) | 1.42 (1.36-1.49) | <0.001 |
| HF hospitalization | 19.0 (18.4-19.5) | 30.4 (29.2-31.7) | 1.61 (1.53-1.69) | <0.001 |
| Non-CV hospitalization | 30.8 (30.0-31.6) | 32.8 (31.5-34.2) | 1.07 (1.02-1.12) | 0.01 |
| Stroke/TIA hospitalization | 2.5 (2.3-2.7) | 2.4 (2.1-2.7) | 0.94 (0.82-1.08) | 0.421 |
| **Trial scenario, AF+digoxin sensitivity** | |  |  |  |
| All-cause death | 22.1 (21.6-22.6) | 24.0 (23.0-25.0) | 1.09 (1.04-1.14) | <0.001 |
| CV death | 14.9 (14.5-15.3) | 17.2 (16.4-18.0) | 1.16 (1.09-1.22) | <0.001 |
| Non-CV death | 7.2 (6.9-7.5) | 6.8 (6.3-7.3) | 0.95 (0.87-1.03) | 0.204 |
| All-cause hospitalization | 60.9 (59.6-62.2) | 70.4 (67.8-73.2) | 1.16 (1.11-1.21) | <0.001 |
| CV hospitalization | 32.0 (31.2-32.8) | 41.9 (40.1-43.7) | 1.31 (1.25-1.38) | <0.001 |
| HF hospitalization | 19.9 (19.4-20.5) | 28.9 (27.6-30.3) | 1.45 (1.37-1.53) | <0.001 |
| Non-CV hospitalization | 31.1 (30.3-31.9) | 32.0 (30.6-33.6) | 1.03 (0.98-1.09) | 0.269 |
| Stroke/TIA hospitalization | 2.5 (2.4-2.7) | 2.2 (2.0-2.6) | 0.89 (0.76-1.03) | 0.126 |
| **Trial scenario, AF sensitivity** | |  |  |  |
| All-cause death | 23.0 (22.5-23.5) | 19.1 (18.0-20.3) | 0.83 (0.78-0.89) | <0.001 |
| CV death | 15.7 (15.3-16.1) | 13.6 (12.6-14.6) | 0.87 (0.80-0.93) | <0.001 |
| Non-CV death | 7.3 (7.0-7.6) | 5.5 (4.9-6.2) | 0.76 (0.67-0.85) | <0.001 |
| All-cause hospitalization | 64.1 (62.8-65.3) | 54.6 (51.6-57.8) | 0.85 (0.80-0.91) | <0.001 |
| CV hospitalization | 34.2 (33.5-35.0) | 32.2 (30.2-34.3) | 0.94 (0.88-1.01) | 0.074 |
| HF hospitalization | 21.6 (21.1-22.2) | 22.7 (21.2-24.4) | 1.05 (0.98-1.13) | 0.183 |
| Non-CV hospitalization | 32.1 (31.3-32.8) | 26.2 (24.4-28.0) | 0.82 (0.76-0.88) | <0.001 |
| Stroke/TIA hospitalization | 2.5 (2.4-2.7) | 1.9 (1.5-2.3) | 0.74 (0.59-0.91) | 0.004 |
| **Pragmatic scenario, BP sensitivity** | |  |  |  |
| All-cause death | 18.1 (17.6-18.6) | 28.9 (28.1-29.7) | 1.60 (1.54-1.66) | <0.001 |
| CV death | 11.9 (11.5-12.3) | 20.5 (19.9-21.2) | 1.73 (1.65-1.81) | <0.001 |
| Non-CV death | 6.2 (5.9-6.5) | 8.4 (8.0-8.8) | 1.35 (1.26-1.45) | <0.001 |
| All-cause hospitalization | 50.7 (49.4-52.0) | 85.1 (82.9-87.4) | 1.68 (1.62-1.74) | <0.001 |
| CV hospitalization | 26.7 (25.9-27.5) | 46.6 (45.3-48.0) | 1.75 (1.67-1.82) | <0.001 |
| HF hospitalization | 15.7 (15.1-16.3) | 32.5 (31.4-33.6) | 2.07 (1.97-2.17) | <0.001 |
| Non-CV hospitalization | 27.2 (26.4-28.0) | 38.0 (36.7-39.2) | 1.39 (1.33-1.46) | <0.001 |
| Stroke/TIA hospitalization | 2.4 (2.2-2.6) | 2.6 (2.3-2.8) | 1.06 (0.94-1.21) | 0.339 |
| **Pragmatic scenario, AF+digoxin sensitivity** | |  |  |  |
| All-cause death | 19.8 (19.3-20.3) | 27.9 (27.1-28.8) | 1.41 (1.35-1.47) | <0.001 |
| CV death | 13.3 (12.9-13.7) | 19.6 (18.9-20.4) | 1.48 (1.41-1.55) | <0.001 |
| Non-CV death | 6.5 (6.2-6.8) | 8.3 (7.8-8.8) | 1.28 (1.18-1.37) | <0.001 |
| All-cause hospitalization | 54.7 (53.4-56.0) | 82.4 (80.0-84.9) | 1.51 (1.45-1.57) | <0.001 |
| CV hospitalization | 29.0 (28.2-29.8) | 45.4 (43.9-46.9) | 1.56 (1.50-1.63) | <0.001 |
| HF hospitalization | 17.6 (17.0-18.2) | 31.5 (30.3-32.6) | 1.79 (1.70-1.88) | <0.001 |
| Non-CV hospitalization | 28.6 (27.8-29.4) | 37.3 (35.9-38.6) | 1.30 (1.25-1.37) | <0.001 |
| Stroke/TIA hospitalization | 2.5 (2.3-2.6) | 2.5 (2.2-2.8) | 1.01 (0.89-1.16) | 0.817 |
| **Pragmatic scenario, AF sensitivity** | |  |  |  |
| All-cause death | 22.6 (22.1-23.1) | 22.1 (21.1-23.2) | 0.98 (0.93-1.03) | 0.408 |
| CV death | 15.5 (15.1-15.9) | 15.1 (14.2-16.0) | 0.97 (0.91-1.04) | 0.436 |
| Non-CV death | 7.1 (6.8-7.4) | 7.0 (6.4-7.6) | 0.98 (0.90-1.08) | 0.761 |
| All-cause hospitalization | 62.8 (61.5-64.1) | 63.5 (60.7-66.4) | 1.01 (0.96-1.06) | 0.652 |
| CV hospitalization | 34.0 (33.2-34.8) | 34.0 (32.3-35.7) | 1.00 (0.94-1.06) | 1 |
| HF hospitalization | 21.4 (20.8-21.9) | 23.8 (22.5-25.2) | 1.12 (1.05-1.19) | 0.001 |
| Non-CV hospitalization | 31.5 (30.7-32.2) | 30.7 (29.1-32.3) | 0.98 (0.92-1.03) | 0.408 |
| Stroke/TIA hospitalization | 2.6 (2.4-2.7) | 2.0 (1.7-2.3) | 0.77 (0.64-0.92) | 0.003 |

**Abbreviations:** AF, atrial fibrillation; BP, blood pressure; CI, confidence interval; CV, cardiovascular; HF, heart failure; IRR, incidence rate ratio; OMT, optimal medical therapy; TIA, transient ischemic attack

**Table S15. Outcomes in eligible and ineligible patients in the trial and pragmatic scenario in in-patients.**

|  | **Events per 100 patient-years (95% CI)** | |  |  |
| --- | --- | --- | --- | --- |
| **Event** | **Ineligible** | **Eligible** | ***IRR*** | ***P*** |
| **Trial scenario** | |  |  |  |
| All-cause death | 37.4 (36.5-38.4) | 33.1 (31.9-34.4) | 0.89 (0.85-0.93) | <0.001 |
| CV death | 25.7 (24.9-26.5) | 23.3 (22.2-24.4) | 0.91 (0.86-0.96) | <0.001 |
| Non-CV death | 11.7 (11.2-12.3) | 9.9 (9.2-10.6) | 0.84 (0.77-0.92) | <0.001 |
| All-cause hospitalization | 110.1 (107.3-113.0) | 109.2 (105.0-113.6) | 0.99 (0.95-1.04) | 0.748 |
| CV hospitalization | 49.4 (48.0-50.9) | 55.9 (53.5-58.4) | 1.13 (1.07-1.19) | <0.001 |
| HF hospitalization | 28.2 (27.2-29.2) | 34.7 (33.0-36.4) | 1.23 (1.16-1.31) | <0.001 |
| Non-CV hospitalization | 51.9 (50.3-53.5) | 46.2 (44.1-48.5) | 0.89 (0.84-0.94) | <0.001 |
| Stroke/TIA hospitalization | 3.6 (3.4-4.0) | 2.7 (2.4-3.1) | 0.75 (0.63-0.88) | <0.001 |
| **Pragmatic scenario** | |  |  |  |
| All-cause death | 33.7 (32.7-34.8) | 38.0 (36.9-39.0) | 1.13 (1.08-1.17) | <0.001 |
| CV death | 23.0 (22.1-23.9) | 26.5 (25.7-27.4) | 1.16 (1.10-1.22) | <0.001 |
| Non-CV death | 10.8 (10.2-11.4) | 11.4 (10.9-12.0) | 1.06 (0.98-1.15) | 0.123 |
| All-cause hospitalization | 96.4 (93.2-99.6) | 122.2 (118.7-125.6) | 1.27 (1.21-1.32) | <0.001 |
| CV hospitalization | 44.6 (42.9-46.3) | 57.4 (55.5-59.2) | 1.29 (1.22-1.35) | <0.001 |
| HF hospitalization | 23.8 (22.7-24.9) | 36.0 (34.7-37.3) | 1.51 (1.43-1.60) | <0.001 |
| Non-CV hospitalization | 47.0 (45.2-48.8) | 52.7 (51.0-54.6) | 1.12 (1.07-1.18) | <0.001 |
| Stroke/TIA hospitalization | 3.5 (3.1-3.9) | 3.2 (2.9-3.6) | 0.92 (0.80-1.07) | 0.278 |
| **Trial scenario, OMT sensitivity** | |  |  |  |
| All-cause death | 37.6 (36.8-38.5) | 26.5 (24.8-28.2) | 0.70 (0.66-0.75) | <0.001 |
| CV death | 26.1 (25.4-26.8) | 18.0 (16.6-19.5) | 0.69 (0.64-0.75) | <0.001 |
| Non-CV death | 11.6 (11.1-12.1) | 8.4 (7.5-9.5) | 0.73 (0.64-0.82) | <0.001 |
| All-cause hospitalization | 111.9 (109.3-114.5) | 96.6 (90.7-102.9) | 0.86 (0.81-0.92) | <0.001 |
| CV hospitalization | 50.9 (49.6-52.2) | 54.2 (50.5-58.1) | 1.07 (0.99-1.15) | 0.093 |
| HF hospitalization | 29.4 (28.5-30.3) | 35.2 (32.6-38.0) | 1.20 (1.10-1.30) | <0.001 |
| Non-CV hospitalization | 51.7 (50.3-53.1) | 40.4 (37.4-43.5) | 0.78 (0.72-0.85) | <0.001 |
| Stroke/TIA hospitalization | 3.5 (3.2-3.8) | 2.5 (1.9-3.1) | 0.70 (0.55-0.88) | 0.002 |
| **Trial scenario, BP sensitivity** | |  |  |  |
| All-cause death | 37.0 (36.1-38.0) | 34.0 (32.8-35.3) | 0.92 (0.88-0.96) | <0.001 |
| CV death | 25.3 (24.6-26.1) | 24.0 (23.0-25.1) | 0.95 (0.90-1.00) | 0.059 |
| Non-CV death | 11.7 (11.2-12.2) | 10.0 (9.3-10.7) | 0.85 (0.78-0.93) | <0.001 |
| All-cause hospitalization | 109.0 (106.2-111.9) | 111.7 (107.5-116.1) | 1.02 (0.98-1.07) | 0.302 |
| CV hospitalization | 48.8 (47.4-50.3) | 57.1 (54.7-59.6) | 1.17 (1.11-1.23) | <0.001 |
| HF hospitalization | 27.8 (26.8-28.8) | 35.6 (33.9-37.3) | 1.28 (1.21-1.36) | <0.001 |
| Non-CV hospitalization | 51.8 (50.3-53.4) | 46.5 (44.4-48.7) | 0.90 (0.85-0.95) | <0.001 |
| Stroke/TIA hospitalization | 3.6 (3.3-3.9) | 2.8 (2.4-3.2) | 0.76 (0.65-0.89) | 0.001 |
| **Trial scenario, AF+digoxin sensitivity** | |  |  |  |
| All-cause death | 36.9 (36.0-37.8) | 33.6 (32.2-35.1) | 0.91 (0.87-0.96) | <0.001 |
| CV death | 25.4 (24.7-26.2) | 23.4 (22.2-24.7) | 0.92 (0.87-0.98) | 0.006 |
| Non-CV death | 11.5 (11.0-12.0) | 10.2 (9.4-11.0) | 0.88 (0.81-0.97) | 0.007 |
| All-cause hospitalization | 109.7 (107.0-112.5) | 110.1 (105.4-115.0) | 1.00 (0.95-1.06) | 0.888 |
| CV hospitalization | 49.8 (48.4-51.3) | 56.1 (53.4-58.9) | 1.13 (1.06-1.19) | <0.001 |
| HF hospitalization | 28.5 (27.6-29.5) | 35.3 (33.5-37.3) | 1.24 (1.16-1.32) | <0.001 |
| Non-CV hospitalization | 50.9 (49.5-52.5) | 47.6 (45.1-50.1) | 0.93 (0.88-0.99) | 0.023 |
| Stroke/TIA hospitalization | 3.6 (3.3-3.9) | 2.7 (2.3-3.2) | 0.76 (0.63-0.90) | 0.001 |
| **Trial scenario, AF sensitivity** | |  |  |  |
| All-cause death | 37.1 (36.3-38.0) | 27.9 (26.0-29.9) | 0.75 (0.70-0.81) | <0.001 |
| CV death | 25.6 (24.9-26.3) | 19.8 (18.2-21.5) | 0.77 (0.71-0.84) | <0.001 |
| Non-CV death | 11.5 (11.1-12.0) | 8.2 (7.1-9.3) | 0.71 (0.62-0.81) | <0.001 |
| All-cause hospitalization | 111.8 (109.2-114.3) | 94.5 (88.1-101.3) | 0.85 (0.79-0.91) | <0.001 |
| CV hospitalization | 51.6 (50.3-53.0) | 48.4 (44.8-52.3) | 0.94 (0.86-1.02) | 0.129 |
| HF hospitalization | 30.1 (29.2-31.0) | 30.0 (27.5-32.7) | 1.00 (0.91-1.09) | 0.982 |
| Non-CV hospitalization | 51.5 (50.2-52.9) | 39.3 (36.0-42.7) | 0.76 (0.70-0.83) | <0.001 |
| Stroke/TIA hospitalization | 3.5 (3.2-3.8) | 2.2 (1.7-2.9) | 0.64 (0.49-0.83) | 0.001 |
| **Pragmatic scenario, BP sensitivity** | |  |  |  |
| All-cause death | 32.8 (31.7-33.9) | 38.6 (37.6-39.7) | 1.18 (1.13-1.23) | <0.001 |
| CV death | 22.2 (21.3-23.1) | 27.1 (26.2-28.0) | 1.22 (1.16-1.29) | <0.001 |
| Non-CV death | 10.6 (10.0-11.3) | 11.5 (11.0-12.1) | 1.09 (1.01-1.17) | 0.035 |
| All-cause hospitalization | 93.8 (90.7-97.1) | 124.0 (120.6-127.5) | 1.32 (1.26-1.38) | <0.001 |
| CV hospitalization | 43.3 (41.6-45.0) | 58.3 (56.5-60.1) | 1.35 (1.28-1.42) | <0.001 |
| HF hospitalization | 22.8 (21.8-23.9) | 36.7 (35.4-38.0) | 1.61 (1.52-1.70) | <0.001 |
| Non-CV hospitalization | 46.5 (44.7-48.4) | 53.0 (51.3-54.8) | 1.14 (1.08-1.20) | <0.001 |
| Stroke/TIA hospitalization | 3.5 (3.1-3.9) | 3.2 (2.9-3.6) | 0.93 (0.81-1.08) | 0.346 |
| **Pragmatic scenario, AF+digoxin sensitivity** | |  |  |  |
| All-cause death | 34.0 (33.0-35.0) | 38.6 (37.5-39.8) | 1.14 (1.09-1.19) | <0.001 |
| CV death | 23.3 (22.5-24.2) | 26.9 (25.9-27.9) | 1.15 (1.10-1.21) | <0.001 |
| Non-CV death | 10.6 (10.1-11.2) | 11.8 (11.1-12.4) | 1.11 (1.02-1.19) | 0.009 |
| All-cause hospitalization | 99.3 (96.4-102.3) | 124.0 (120.2-127.9) | 1.25 (1.20-1.30) | <0.001 |
| CV hospitalization | 46.4 (44.8-48.0) | 57.7 (55.7-59.8) | 1.24 (1.18-1.31) | <0.001 |
| HF hospitalization | 25.3 (24.3-26.4) | 36.7 (35.2-38.1) | 1.45 (1.37-1.53) | <0.001 |
| Non-CV hospitalization | 47.1 (45.5-48.8) | 54.0 (52.0-56.0) | 1.15 (1.09-1.21) | <0.001 |
| Stroke/TIA hospitalization | 3.4 (3.1-3.8) | 3.2 (2.9-3.6) | 0.94 (0.81-1.08) | 0.385 |
| **Pragmatic scenario, AF sensitivity** | |  |  |  |
| All-cause death | 37.0 (36.2-37.9) | 32.1 (30.5-33.7) | 0.87 (0.82-0.92) | <0.001 |
| CV death | 25.6 (24.9-26.4) | 22.0 (20.7-23.3) | 0.86 (0.80-0.92) | <0.001 |
| Non-CV death | 11.4 (10.9-11.9) | 10.1 (9.2-11.1) | 0.89 (0.80-0.98) | 0.017 |
| All-cause hospitalization | 111.5 (108.8-114.1) | 103.1 (98.0-108.4) | 0.93 (0.87-0.98) | 0.006 |
| CV hospitalization | 52.2 (50.8-53.6) | 47.6 (44.9-50.4) | 0.91 (0.85-0.97) | 0.004 |
| HF hospitalization | 30.2 (29.2-31.1) | 29.8 (27.9-31.8) | 0.99 (0.92-1.06) | 0.758 |
| Non-CV hospitalization | 51.5 (50.0-52.9) | 44.6 (41.9-47.3) | 0.87 (0.81-0.93) | <0.001 |
| Stroke/TIA hospitalization | 3.5 (3.3-3.8) | 2.5 (2.1-3.1) | 0.72 (0.59-0.87) | 0.001 |

**Abbreviations:** AF, atrial fibrillation; BP, blood pressure; CI, confidence interval; CV, cardiovascular; HF, heart failure; IRR, incidence rate ratio; OMT, optimal medical therapy; TIA, transient ischemic attack

**Table S16. Outcomes in eligible and ineligible patients in the trial and pragmatic scenario in out-patients.**

|  | **Events per 100 patient-years (95% CI)** | |  |  |
| --- | --- | --- | --- | --- |
| **Event** | **Ineligible** | **Eligible** | ***IRR*** | ***P*** |
| **Trial scenario** | |  |  |  |
| All-cause death | 13.1 (12.8-13.4) | 17.5 (16.7-18.3) | 1.33 (1.27-1.40) | <0.001 |
| CV death | 7.9 (7.7-8.2) | 12.3 (11.6-12.9) | 1.55 (1.46-1.64) | <0.001 |
| Non-CV death | 5.2 (5.0-5.4) | 5.2 (4.8-5.7) | 1.01 (0.92-1.10) | 0.841 |
| All-cause hospitalization | 41.8 (41.0-42.5) | 55.1 (53.0-57.2) | 1.32 (1.26-1.38) | <0.001 |
| CV hospitalization | 20.6 (20.2-21.1) | 30.5 (29.2-31.8) | 1.48 (1.41-1.55) | <0.001 |
| HF hospitalization | 11.5 (11.2-11.8) | 20.8 (19.9-21.9) | 1.81 (1.72-1.91) | <0.001 |
| Non-CV hospitalization | 24.9 (24.3-25.4) | 29.0 (27.7-30.3) | 1.17 (1.11-1.23) | <0.001 |
| Stroke/TIA hospitalization | 2.0 (1.9-2.1) | 2.1 (1.8-2.3) | 1.04 (0.90-1.20) | 0.606 |
| **Pragmatic scenario** | |  |  |  |
| All-cause death | 12.0 (11.8-12.3) | 20.0 (19.3-20.7) | 1.66 (1.59-1.73) | <0.001 |
| CV death | 7.3 (7.0-7.5) | 13.3 (12.8-13.9) | 1.84 (1.74-1.94) | <0.001 |
| Non-CV death | 4.8 (4.6-5.0) | 6.7 (6.3-7.1) | 1.39 (1.30-1.50) | <0.001 |
| All-cause hospitalization | 39.2 (38.4-39.9) | 62.2 (60.3-64.1) | 1.59 (1.53-1.65) | <0.001 |
| CV hospitalization | 19.2 (18.8-19.7) | 33.2 (32.0-34.3) | 1.72 (1.65-1.80) | <0.001 |
| HF hospitalization | 10.4 (10.1-10.7) | 22.6 (21.7-23.5) | 2.17 (2.07-2.28) | <0.001 |
| Non-CV hospitalization | 23.5 (23.0-24.0) | 33.1 (32.0-34.3) | 1.41 (1.35-1.47) | <0.001 |
| Stroke/TIA hospitalization | 1.9 (1.8-2.1) | 2.2 (1.9-2.4) | 1.12 (0.99-1.27) | 0.073 |
| **Trial scenario, OMT sensitivity** | |  |  |  |
| All-cause death | 13.7 (13.4-14.0) | 15.7 (14.7-16.7) | 1.15 (1.07-1.23) | <0.001 |
| CV death | 8.4 (8.2-8.6) | 10.9 (10.1-11.8) | 1.30 (1.20-1.41) | <0.001 |
| Non-CV death | 5.3 (5.1-5.4) | 4.7 (4.2-5.3) | 0.90 (0.79-1.02) | 0.092 |
| All-cause hospitalization | 43.2 (42.4-43.9) | 50.0 (47.4-52.7) | 1.16 (1.09-1.22) | <0.001 |
| CV hospitalization | 21.5 (21.0-21.9) | 29.4 (27.7-31.2) | 1.37 (1.29-1.46) | <0.001 |
| HF hospitalization | 12.2 (11.9-12.5) | 20.6 (19.3-22.0) | 1.68 (1.57-1.81) | <0.001 |
| Non-CV hospitalization | 25.5 (25.0-26.0) | 25.8 (24.2-27.5) | 1.01 (0.95-1.08) | 0.672 |
| Stroke/TIA hospitalization | 2.0 (1.9-2.1) | 1.8 (1.4-2.2) | 0.88 (0.71-1.08) | 0.224 |
| **Trial scenario, BP sensitivity** | |  |  |  |
| All-cause death | 13.1 (12.8-13.4) | 17.7 (17.0-18.5) | 1.36 (1.29-1.42) | <0.001 |
| CV death | 7.9 (7.6-8.1) | 12.4 (11.8-13.1) | 1.58 (1.49-1.67) | <0.001 |
| Non-CV death | 5.2 (5.0-5.4) | 5.3 (4.9-5.7) | 1.02 (0.94-1.11) | 0.626 |
| All-cause hospitalization | 41.6 (40.9-42.4) | 55.8 (53.8-58.0) | 1.34 (1.29-1.40) | <0.001 |
| CV hospitalization | 20.5 (20.1-21.0) | 30.9 (29.7-32.3) | 1.51 (1.44-1.58) | <0.001 |
| HF hospitalization | 11.4 (11.1-11.7) | 21.2 (20.3-22.2) | 1.86 (1.76-1.96) | <0.001 |
| Non-CV hospitalization | 24.8 (24.3-25.3) | 29.2 (28.0-30.5) | 1.18 (1.12-1.24) | <0.001 |
| Stroke/TIA hospitalization | 2.0 (1.9-2.1) | 2.1 (1.8-2.3) | 1.04 (0.90-1.20) | 0.61 |
| **Trial scenario, AF+digoxin sensitivity** | |  |  |  |
| All-cause death | 13.3 (13.0-13.6) | 16.9 (16.1-17.7) | 1.26 (1.20-1.33) | <0.001 |
| CV death | 8.1 (7.9-8.4) | 11.7 (11.0-12.4) | 1.44 (1.35-1.54) | <0.001 |
| Non-CV death | 5.2 (5.0-5.4) | 5.2 (4.7-5.6) | 0.99 (0.90-1.09) | 0.887 |
| All-cause hospitalization | 42.3 (41.5-43.0) | 53.7 (51.5-55.9) | 1.27 (1.21-1.33) | <0.001 |
| CV hospitalization | 20.9 (20.5-21.4) | 30.0 (28.6-31.4) | 1.43 (1.36-1.51) | <0.001 |
| HF hospitalization | 11.8 (11.5-12.1) | 20.2 (19.2-21.3) | 1.71 (1.62-1.81) | <0.001 |
| Non-CV hospitalization | 25.1 (24.5-25.6) | 28.4 (27.0-29.8) | 1.13 (1.07-1.19) | <0.001 |
| Stroke/TIA hospitalization | 2.0 (1.9-2.1) | 2.0 (1.7-2.3) | 0.98 (0.83-1.14) | 0.815 |
| **Trial scenario, AF sensitivity** | |  |  |  |
| All-cause death | 13.9 (13.6-14.2) | 13.1 (12.2-14.1) | 0.94 (0.87-1.02) | 0.125 |
| CV death | 8.6 (8.4-8.8) | 8.8 (8.1-9.6) | 1.02 (0.93-1.12) | 0.636 |
| Non-CV death | 5.3 (5.1-5.5) | 4.3 (3.8-4.9) | 0.81 (0.71-0.93) | 0.001 |
| All-cause hospitalization | 44.1 (43.4-44.8) | 39.5 (37.2-42.0) | 0.90 (0.84-0.96) | 0.001 |
| CV hospitalization | 22.1 (21.7-22.5) | 22.0 (20.5-23.6) | 1.00 (0.92-1.07) | 0.912 |
| HF hospitalization | 12.7 (12.4-13.0) | 14.7 (13.5-15.9) | 1.15 (1.06-1.25) | 0.001 |
| Non-CV hospitalization | 25.8 (25.3-26.3) | 22.3 (20.7-23.9) | 0.86 (0.80-0.93) | <0.001 |
| Stroke/TIA hospitalization | 2.0 (1.9-2.1) | 1.7 (1.3-2.0) | 0.82 (0.65-1.01) | 0.071 |
| **Pragmatic scenario, BP sensitivity** | |  |  |  |
| All-cause death | 11.9 (11.6-12.2) | 20.3 (19.6-21.0) | 1.70 (1.63-1.77) | <0.001 |
| CV death | 7.2 (6.9-7.4) | 13.5 (12.9-14.1) | 1.88 (1.79-1.98) | <0.001 |
| Non-CV death | 4.7 (4.6-4.9) | 6.8 (6.4-7.2) | 1.42 (1.33-1.53) | <0.001 |
| All-cause hospitalization | 38.8 (38.1-39.6) | 63.2 (61.3-65.1) | 1.63 (1.57-1.69) | <0.001 |
| CV hospitalization | 19.0 (18.6-19.5) | 33.7 (32.5-34.8) | 1.77 (1.70-1.84) | <0.001 |
| HF hospitalization | 10.3 (10.0-10.6) | 23.0 (22.1-23.9) | 2.24 (2.14-2.35) | <0.001 |
| Non-CV hospitalization | 23.3 (22.8-23.8) | 33.5 (32.4-34.7) | 1.44 (1.38-1.50) | <0.001 |
| Stroke/TIA hospitalization | 1.9 (1.8-2.1) | 2.2 (2.0-2.4) | 1.12 (0.99-1.27) | 0.066 |
| **Pragmatic scenario, AF+digoxin sensitivity** | |  |  |  |
| All-cause death | 12.4 (12.2-12.7) | 19.6 (18.9-20.3) | 1.57 (1.50-1.65) | <0.001 |
| CV death | 7.6 (7.4-7.8) | 13.0 (12.4-13.6) | 1.71 (1.62-1.81) | <0.001 |
| Non-CV death | 4.9 (4.7-5.1) | 6.6 (6.2-7.0) | 1.36 (1.26-1.46) | <0.001 |
| All-cause hospitalization | 40.1 (39.3-40.8) | 61.2 (59.2-63.3) | 1.53 (1.47-1.59) | <0.001 |
| CV hospitalization | 19.8 (19.3-20.2) | 32.8 (31.6-34.1) | 1.66 (1.59-1.74) | <0.001 |
| HF hospitalization | 10.9 (10.6-11.2) | 22.1 (21.1-23.0) | 2.02 (1.92-2.12) | <0.001 |
| Non-CV hospitalization | 23.9 (23.4-24.4) | 32.7 (31.4-33.9) | 1.37 (1.31-1.43) | <0.001 |
| Stroke/TIA hospitalization | 2.0 (1.9-2.1) | 2.1 (1.8-2.3) | 1.04 (0.91-1.19) | 0.559 |
| **Pragmatic scenario, AF sensitivity** | |  |  |  |
| All-cause death | 13.7 (13.4-14.0) | 14.9 (14.0-15.8) | 1.09 (1.02-1.16) | 0.009 |
| CV death | 8.5 (8.3-8.8) | 9.6 (8.9-10.3) | 1.12 (1.04-1.21) | 0.004 |
| Non-CV death | 5.2 (5.0-5.4) | 5.3 (4.8-5.9) | 1.03 (0.92-1.14) | 0.593 |
| All-cause hospitalization | 43.5 (42.8-44.3) | 45.7 (43.4-48.0) | 1.05 (1.00-1.11) | 0.07 |
| CV hospitalization | 21.9 (21.4-22.3) | 24.0 (22.6-25.4) | 1.10 (1.03-1.17) | 0.004 |
| HF hospitalization | 12.5 (12.2-12.8) | 16.2 (15.1-17.3) | 1.29 (1.20-1.39) | <0.001 |
| Non-CV hospitalization | 25.5 (25.0-26.0) | 25.8 (24.3-27.3) | 1.01 (0.95-1.08) | 0.696 |
| Stroke/TIA hospitalization | 2.0 (1.9-2.2) | 1.7 (1.4-2.0) | 0.82 (0.67-0.98) | 0.035 |

**Abbreviations:** AF, atrial fibrillation; BP, blood pressure; CI, confidence interval; CV, cardiovascular; HF, heart failure; IRR, incidence rate ratio; OMT, optimal medical therapy; TIA, transient ischemic attack

**Table S17. Outcomes in eligible and ineligible patients in the trial and pragmatic scenario in patients with severe heart failure.**

|  | **Events per 100 patient-years (95% CI)** | |  |  |
| --- | --- | --- | --- | --- |
| **Event** | **Ineligible** | **Eligible** | ***IRR*** | ***P*** |
| **Trial scenario** | |  |  |  |
| All-cause death | 40.0 (38.6-41.4) | 39.5 (37.6-41.4) | 0.99 (0.93-1.05) | 0.694 |
| CV death | 28.0 (26.8-29.2) | 29.7 (28.1-31.4) | 1.06 (0.99-1.14) | 0.1 |
| Non-CV death | 12.0 (11.2-12.8) | 9.8 (8.9-10.8) | 0.82 (0.73-0.92) | 0.001 |
| All-cause hospitalization | 107.1 (103.3-111.1) | 122.7 (116.8-128.8) | 1.15 (1.08-1.22) | <0.001 |
| CV hospitalization | 54.2 (52.1-56.5) | 71.3 (67.6-75.2) | 1.31 (1.23-1.41) | <0.001 |
| HF hospitalization | 34.6 (33.0-36.2) | 49.2 (46.5-52.2) | 1.42 (1.32-1.53) | <0.001 |
| Non-CV hospitalization | 47.9 (45.9-50.1) | 45.9 (43.1-48.7) | 0.96 (0.89-1.03) | 0.249 |
| Stroke/TIA hospitalization | 3.6 (3.2-4.1) | 3.0 (2.5-3.6) | 0.82 (0.65-1.02) | 0.07 |
| **Pragmatic scenario** | |  |  |  |
| All-cause death | 35.8 (34.1-37.4) | 42.8 (41.3-44.4) | 1.20 (1.13-1.27) | <0.001 |
| CV death | 25.0 (23.7-26.5) | 31.3 (30.0-32.6) | 1.25 (1.16-1.34) | <0.001 |
| Non-CV death | 10.7 (9.8-11.6) | 11.5 (10.7-12.4) | 1.08 (0.96-1.20) | 0.184 |
| All-cause hospitalization | 91.6 (87.4-95.9) | 131.5 (126.7-136.5) | 1.44 (1.35-1.53) | <0.001 |
| CV hospitalization | 48.0 (45.5-50.6) | 70.0 (67.2-73.0) | 1.46 (1.36-1.56) | <0.001 |
| HF hospitalization | 29.0 (27.3-30.8) | 48.7 (46.5-50.9) | 1.68 (1.55-1.81) | <0.001 |
| Non-CV hospitalization | 44.1 (41.7-46.6) | 49.7 (47.4-52.1) | 1.13 (1.05-1.21) | 0.001 |
| Stroke/TIA hospitalization | 3.7 (3.2-4.3) | 3.2 (2.8-3.7) | 0.87 (0.70-1.06) | 0.164 |
| **Trial scenario, OMT sensitivity** | |  |  |  |
| All-cause death | 41.6 (40.4-42.9) | 31.0 (28.7-33.5) | 0.75 (0.68-0.81) | <0.001 |
| CV death | 30.0 (28.9-31.1) | 22.1 (20.2-24.3) | 0.74 (0.67-0.82) | <0.001 |
| Non-CV death | 11.7 (11.0-12.4) | 8.9 (7.7-10.3) | 0.76 (0.65-0.89) | 0.001 |
| All-cause hospitalization | 111.9 (108.4-115.5) | 114.1 (105.8-122.8) | 1.02 (0.94-1.11) | 0.635 |
| CV hospitalization | 57.9 (55.8-60.0) | 69.8 (64.4-75.7) | 1.21 (1.10-1.32) | <0.001 |
| HF hospitalization | 37.9 (36.4-39.4) | 47.0 (43.0-51.3) | 1.24 (1.13-1.37) | <0.001 |
| Non-CV hospitalization | 48.7 (46.9-50.6) | 40.1 (36.5-44.0) | 0.82 (0.74-0.91) | <0.001 |
| Stroke/TIA hospitalization | 3.6 (3.2-4.0) | 2.6 (2.0-3.5) | 0.74 (0.54-0.99) | 0.044 |
| **Trial scenario, BP sensitivity** | |  |  |  |
| All-cause death | 39.4 (38.0-40.9) | 40.4 (38.6-42.3) | 1.02 (0.97-1.09) | 0.425 |
| CV death | 27.5 (26.3-28.7) | 30.6 (29.0-32.3) | 1.11 (1.04-1.19) | 0.002 |
| Non-CV death | 12.0 (11.2-12.8) | 9.8 (8.9-10.8) | 0.82 (0.73-0.92) | 0.001 |
| All-cause hospitalization | 105.1 (101.2-109.0) | 126.5 (120.6-132.6) | 1.20 (1.13-1.28) | <0.001 |
| CV hospitalization | 53.1 (50.9-55.3) | 73.3 (69.6-77.2) | 1.38 (1.29-1.48) | <0.001 |
| HF hospitalization | 33.6 (32.0-35.2) | 50.9 (48.1-53.8) | 1.52 (1.41-1.63) | <0.001 |
| Non-CV hospitalization | 47.7 (45.6-49.9) | 46.3 (43.6-49.1) | 0.97 (0.90-1.04) | 0.419 |
| Stroke/TIA hospitalization | 3.6 (3.2-4.1) | 3.0 (2.5-3.6) | 0.84 (0.67-1.04) | 0.113 |
| **Trial scenario, AF+digoxin sensitivity** | |  |  |  |
| All-cause death | 40.2 (38.8-41.5) | 38.9 (36.9-41.0) | 0.97 (0.91-1.03) | 0.33 |
| CV death | 28.5 (27.3-29.6) | 29.0 (27.3-30.9) | 1.02 (0.95-1.10) | 0.611 |
| Non-CV death | 11.7 (11.0-12.5) | 9.9 (8.9-11.0) | 0.85 (0.75-0.96) | 0.007 |
| All-cause hospitalization | 108.5 (104.8-112.4) | 122.3 (115.8-129.1) | 1.13 (1.06-1.20) | <0.001 |
| CV hospitalization | 55.7 (53.6-57.9) | 70.8 (66.7-75.1) | 1.27 (1.18-1.36) | <0.001 |
| HF hospitalization | 35.9 (34.3-37.5) | 48.7 (45.6-51.9) | 1.36 (1.25-1.47) | <0.001 |
| Non-CV hospitalization | 47.4 (45.5-49.5) | 46.6 (43.6-49.8) | 0.98 (0.91-1.06) | 0.689 |
| Stroke/TIA hospitalization | 3.6 (3.2-4.1) | 2.8 (2.3-3.5) | 0.78 (0.61-0.99) | 0.038 |
| **Trial scenario, AF sensitivity** | |  |  |  |
| All-cause death | 40.8 (39.6-42.0) | 33.2 (30.4-36.2) | 0.81 (0.74-0.89) | <0.001 |
| CV death | 29.2 (28.1-30.2) | 25.0 (22.6-27.6) | 0.86 (0.77-0.95) | 0.004 |
| Non-CV death | 11.6 (11.0-12.3) | 8.2 (6.8-9.7) | 0.70 (0.58-0.84) | <0.001 |
| All-cause hospitalization | 112.6 (109.1-116.1) | 109.5 (100.1-119.5) | 0.97 (0.88-1.07) | 0.574 |
| CV hospitalization | 59.3 (57.3-61.4) | 61.6 (55.8-67.8) | 1.04 (0.93-1.15) | 0.474 |
| HF hospitalization | 38.7 (37.3-40.3) | 42.8 (38.4-47.4) | 1.10 (0.98-1.23) | 0.083 |
| Non-CV hospitalization | 48.0 (46.2-49.9) | 41.5 (37.2-46.1) | 0.86 (0.77-0.97) | 0.01 |
| Stroke/TIA hospitalization | 3.5 (3.2-3.9) | 2.6 (1.8-3.6) | 0.74 (0.51-1.03) | 0.08 |
| **Pragmatic scenario, BP sensitivity** | |  |  |  |
| All-cause death | 34.4 (32.7-36.0) | 43.6 (42.1-45.2) | 1.27 (1.19-1.35) | <0.001 |
| CV death | 23.8 (22.4-25.2) | 32.0 (30.7-33.4) | 1.35 (1.25-1.45) | <0.001 |
| Non-CV death | 10.6 (9.7-11.6) | 11.6 (10.8-12.4) | 1.09 (0.98-1.22) | 0.119 |
| All-cause hospitalization | 86.8 (82.7-91.1) | 135.0 (130.1-140.0) | 1.55 (1.46-1.65) | <0.001 |
| CV hospitalization | 45.5 (43.1-48.0) | 71.7 (68.8-74.6) | 1.57 (1.47-1.69) | <0.001 |
| HF hospitalization | 27.0 (25.3-28.7) | 50.0 (47.8-52.2) | 1.85 (1.72-2.01) | <0.001 |
| Non-CV hospitalization | 43.2 (40.7-45.7) | 50.2 (48.0-52.5) | 1.16 (1.08-1.25) | <0.001 |
| Stroke/TIA hospitalization | 3.7 (3.1-4.3) | 3.2 (2.8-3.7) | 0.87 (0.71-1.08) | 0.195 |
| **Pragmatic scenario, AF+digoxin sensitivity** | |  |  |  |
| All-cause death | 37.3 (35.8-38.8) | 42.6 (40.9-44.3) | 1.14 (1.08-1.21) | <0.001 |
| CV death | 26.4 (25.2-27.7) | 31.1 (29.6-32.6) | 1.17 (1.10-1.26) | <0.001 |
| Non-CV death | 10.9 (10.1-11.7) | 11.5 (10.7-12.5) | 1.06 (0.95-1.19) | 0.272 |
| All-cause hospitalization | 97.2 (93.3-101.3) | 132.7 (127.3-138.3) | 1.37 (1.29-1.45) | <0.001 |
| CV hospitalization | 51.2 (48.9-53.7) | 70.5 (67.4-73.8) | 1.38 (1.29-1.47) | <0.001 |
| HF hospitalization | 32.0 (30.4-33.8) | 48.8 (46.4-51.3) | 1.52 (1.42-1.64) | <0.001 |
| Non-CV hospitalization | 44.7 (42.5-47.0) | 50.2 (47.6-52.8) | 1.12 (1.04-1.20) | 0.002 |
| Stroke/TIA hospitalization | 3.7 (3.2-4.2) | 3.1 (2.7-3.6) | 0.85 (0.69-1.05) | 0.125 |
| **Pragmatic scenario, AF sensitivity** | |  |  |  |
| All-cause death | 41.0 (39.7-42.3) | 35.4 (33.1-37.8) | 0.86 (0.80-0.93) | <0.001 |
| CV death | 29.5 (28.4-30.6) | 25.4 (23.5-27.5) | 0.86 (0.79-0.94) | 0.001 |
| Non-CV death | 11.5 (10.8-12.2) | 9.9 (8.7-11.3) | 0.86 (0.75-0.99) | 0.037 |
| All-cause hospitalization | 112.2 (108.6-115.9) | 112.3 (104.9-120.2) | 1.00 (0.93-1.08) | 0.97 |
| CV hospitalization | 60.4 (58.3-62.6) | 55.8 (51.6-60.2) | 0.92 (0.85-1.00) | 0.063 |
| HF hospitalization | 39.1 (37.5-40.7) | 39.6 (36.4-43.0) | 1.01 (0.92-1.11) | 0.76 |
| Non-CV hospitalization | 48.1 (46.2-50.0) | 43.7 (40.1-47.4) | 0.91 (0.83-0.99) | 0.037 |
| Stroke/TIA hospitalization | 3.6 (3.2-4.0) | 2.6 (2.0-3.3) | 0.71 (0.53-0.94) | 0.014 |

**Abbreviations:** AF, atrial fibrillation; BP, blood pressure; CI, confidence interval; CV, cardiovascular; HF, heart failure; IRR, incidence rate ratio; OMT, optimal medical therapy; TIA, transient ischemic attack

**Table S18. Outcomes in eligible and ineligible patients in the trial and pragmatic scenario in patients with NYHA class III-IV.**

|  | **Events per 100 patient-years (95% CI)** | |  |  |
| --- | --- | --- | --- | --- |
| **Event** | **Ineligible** | **Eligible** | ***IRR*** | ***P*** |
| **Trial scenario** | |  |  |  |
| All-cause death | 29.6 (29.0-30.3) | 32.7 (31.6-33.9) | 1.11 (1.06-1.15) | <0.001 |
| CV death | 19.6 (19.1-20.1) | 23.6 (22.7-24.6) | 1.21 (1.15-1.27) | <0.001 |
| Non-CV death | 10.0 (9.7-10.4) | 9.1 (8.5-9.7) | 0.91 (0.84-0.98) | 0.01 |
| All-cause hospitalization | 84.8 (83.1-86.5) | 109.7 (106.1-113.4) | 1.29 (1.24-1.35) | <0.001 |
| CV hospitalization | 41.5 (40.5-42.5) | 57.9 (55.8-60.1) | 1.40 (1.34-1.46) | <0.001 |
| HF hospitalization | 24.8 (24.1-25.5) | 38.7 (37.2-40.3) | 1.56 (1.48-1.64) | <0.001 |
| Non-CV hospitalization | 44.5 (43.4-45.5) | 46.0 (44.1-47.9) | 1.03 (0.99-1.08) | 0.163 |
| Stroke/TIA hospitalization | 3.0 (2.7-3.2) | 2.8 (2.5-3.2) | 0.96 (0.83-1.10) | 0.576 |
| **Pragmatic scenario** | |  |  |  |
| All-cause death | 25.6 (25.0-26.3) | 37.4 (36.4-38.4) | 1.46 (1.41-1.51) | <0.001 |
| CV death | 16.7 (16.2-17.3) | 26.3 (25.5-27.1) | 1.57 (1.50-1.64) | <0.001 |
| Non-CV death | 8.9 (8.5-9.3) | 11.1 (10.6-11.6) | 1.24 (1.17-1.33) | <0.001 |
| All-cause hospitalization | 74.4 (72.6-76.2) | 119.9 (116.9-123.0) | 1.61 (1.56-1.67) | <0.001 |
| CV hospitalization | 36.7 (35.7-37.7) | 59.9 (58.2-61.6) | 1.63 (1.57-1.70) | <0.001 |
| HF hospitalization | 21.1 (20.4-21.8) | 40.0 (38.8-41.3) | 1.90 (1.81-1.98) | <0.001 |
| Non-CV hospitalization | 40.5 (39.4-41.6) | 51.7 (50.1-53.3) | 1.28 (1.22-1.33) | <0.001 |
| Stroke/TIA hospitalization | 2.8 (2.6-3.1) | 3.1 (2.8-3.4) | 1.09 (0.96-1.23) | 0.179 |
| **Trial scenario, OMT sensitivity** | |  |  |  |
| All-cause death | 31.0 (30.4-31.6) | 26.2 (24.8-27.7) | 0.85 (0.80-0.90) | <0.001 |
| CV death | 20.9 (20.5-21.4) | 18.4 (17.2-19.6) | 0.88 (0.82-0.94) | <0.001 |
| Non-CV death | 10.1 (9.7-10.4) | 7.8 (7.1-8.7) | 0.78 (0.70-0.87) | <0.001 |
| All-cause hospitalization | 89.7 (88.1-91.4) | 95.3 (90.6-100.3) | 1.06 (1.01-1.12) | 0.029 |
| CV hospitalization | 44.1 (43.2-45.1) | 54.0 (51.1-57.1) | 1.22 (1.15-1.30) | <0.001 |
| HF hospitalization | 26.9 (26.3-27.6) | 37.1 (34.9-39.4) | 1.38 (1.29-1.47) | <0.001 |
| Non-CV hospitalization | 45.6 (44.7-46.7) | 39.4 (37.1-41.9) | 0.86 (0.81-0.92) | <0.001 |
| Stroke/TIA hospitalization | 3.0 (2.8-3.2) | 2.5 (2.0-2.9) | 0.82 (0.67-1.00) | 0.048 |
| **Trial scenario, BP sensitivity** | |  |  |  |
| All-cause death | 29.4 (28.7-30.0) | 33.3 (32.2-34.4) | 1.13 (1.09-1.18) | <0.001 |
| CV death | 19.3 (18.8-19.8) | 24.1 (23.2-25.1) | 1.25 (1.19-1.31) | <0.001 |
| Non-CV death | 10.0 (9.7-10.4) | 9.2 (8.6-9.8) | 0.91 (0.85-0.98) | 0.014 |
| All-cause hospitalization | 84.1 (82.3-85.8) | 111.5 (107.9-115.3) | 1.33 (1.28-1.38) | <0.001 |
| CV hospitalization | 41.0 (40.1-42.0) | 58.9 (56.8-61.1) | 1.43 (1.37-1.50) | <0.001 |
| HF hospitalization | 24.5 (23.8-25.1) | 39.6 (38.1-41.2) | 1.62 (1.54-1.70) | <0.001 |
| Non-CV hospitalization | 44.4 (43.3-45.5) | 46.2 (44.4-48.0) | 1.04 (0.99-1.09) | 0.095 |
| Stroke/TIA hospitalization | 3.0 (2.8-3.2) | 2.8 (2.5-3.2) | 0.96 (0.83-1.10) | 0.556 |
| **Trial scenario, AF+digoxin sensitivity** | |  |  |  |
| All-cause death | 29.8 (29.2-30.5) | 32.6 (31.4-33.8) | 1.09 (1.05-1.14) | <0.001 |
| CV death | 19.9 (19.4-20.4) | 23.3 (22.3-24.4) | 1.17 (1.11-1.23) | <0.001 |
| Non-CV death | 10.0 (9.6-10.3) | 9.3 (8.6-9.9) | 0.93 (0.86-1.01) | 0.08 |
| All-cause hospitalization | 86.0 (84.3-87.7) | 109.5 (105.5-113.5) | 1.27 (1.22-1.33) | <0.001 |
| CV hospitalization | 42.3 (41.4-43.3) | 57.6 (55.3-60.0) | 1.36 (1.30-1.43) | <0.001 |
| HF hospitalization | 25.5 (24.9-26.2) | 38.7 (37.0-40.5) | 1.52 (1.44-1.60) | <0.001 |
| Non-CV hospitalization | 44.4 (43.4-45.4) | 46.5 (44.5-48.6) | 1.05 (1.00-1.10) | 0.064 |
| Stroke/TIA hospitalization | 3.0 (2.8-3.2) | 2.8 (2.4-3.2) | 0.94 (0.81-1.09) | 0.455 |
| **Trial scenario, AF sensitivity** | |  |  |  |
| All-cause death | 30.9 (30.3-31.5) | 26.5 (24.9-28.1) | 0.86 (0.80-0.91) | <0.001 |
| CV death | 20.8 (20.3-21.3) | 18.9 (17.5-20.3) | 0.91 (0.84-0.98) | 0.012 |
| Non-CV death | 10.1 (9.7-10.4) | 7.6 (6.7-8.5) | 0.75 (0.67-0.85) | <0.001 |
| All-cause hospitalization | 90.5 (88.8-92.2) | 89.2 (84.1-94.6) | 0.99 (0.93-1.05) | 0.674 |
| CV hospitalization | 45.0 (44.0-45.9) | 47.5 (44.4-50.7) | 1.06 (0.99-1.13) | 0.119 |
| HF hospitalization | 27.6 (27.0-28.3) | 31.7 (29.4-34.0) | 1.15 (1.06-1.24) | 0.001 |
| Non-CV hospitalization | 45.6 (44.6-46.6) | 38.3 (35.7-41.1) | 0.84 (0.78-0.90) | <0.001 |
| Stroke/TIA hospitalization | 3.0 (2.8-3.2) | 2.4 (2.0-3.0) | 0.82 (0.65-1.02) | 0.075 |
| **Pragmatic scenario, BP sensitivity** | |  |  |  |
| All-cause death | 25.0 (24.4-25.7) | 37.9 (36.9-38.9) | 1.51 (1.46-1.57) | <0.001 |
| CV death | 16.2 (15.7-16.7) | 26.7 (26.0-27.6) | 1.65 (1.58-1.73) | <0.001 |
| Non-CV death | 8.8 (8.4-9.2) | 11.1 (10.6-11.7) | 1.26 (1.18-1.35) | <0.001 |
| All-cause hospitalization | 72.7 (70.9-74.4) | 121.9 (118.9-125.0) | 1.68 (1.62-1.74) | <0.001 |
| CV hospitalization | 35.8 (34.8-36.8) | 60.8 (59.1-62.6) | 1.70 (1.63-1.77) | <0.001 |
| HF hospitalization | 20.4 (19.7-21.1) | 40.8 (39.6-42.1) | 2.00 (1.91-2.10) | <0.001 |
| Non-CV hospitalization | 40.0 (38.9-41.1) | 52.2 (50.6-53.7) | 1.30 (1.25-1.36) | <0.001 |
| Stroke/TIA hospitalization | 2.8 (2.6-3.1) | 3.1 (2.8-3.3) | 1.08 (0.96-1.23) | 0.192 |
| **Pragmatic scenario, AF+digoxin sensitivity** | |  |  |  |
| All-cause death | 26.7 (26.1-27.3) | 37.7 (36.7-38.8) | 1.41 (1.36-1.47) | <0.001 |
| CV death | 17.6 (17.1-18.2) | 26.5 (25.6-27.4) | 1.50 (1.44-1.57) | <0.001 |
| Non-CV death | 9.1 (8.7-9.5) | 11.2 (10.7-11.8) | 1.24 (1.16-1.32) | <0.001 |
| All-cause hospitalization | 77.9 (76.2-79.6) | 120.3 (117.0-123.7) | 1.54 (1.49-1.60) | <0.001 |
| CV hospitalization | 38.7 (37.7-39.7) | 59.9 (58.1-61.9) | 1.55 (1.49-1.61) | <0.001 |
| HF hospitalization | 22.7 (22.0-23.4) | 40.3 (38.9-41.7) | 1.77 (1.69-1.86) | <0.001 |
| Non-CV hospitalization | 41.3 (40.3-42.4) | 52.2 (50.5-54.0) | 1.26 (1.21-1.32) | <0.001 |
| Stroke/TIA hospitalization | 2.9 (2.7-3.1) | 3.0 (2.7-3.4) | 1.06 (0.93-1.20) | 0.381 |
| **Pragmatic scenario, AF sensitivity** | |  |  |  |
| All-cause death | 30.4 (29.8-31.0) | 30.7 (29.3-32.1) | 1.01 (0.96-1.06) | 0.699 |
| CV death | 20.5 (20.0-21.0) | 21.2 (20.0-22.4) | 1.03 (0.97-1.10) | 0.332 |
| Non-CV death | 9.9 (9.5-10.2) | 9.5 (8.8-10.4) | 0.97 (0.88-1.06) | 0.482 |
| All-cause hospitalization | 89.0 (87.4-90.7) | 98.8 (94.4-103.4) | 1.11 (1.06-1.17) | <0.001 |
| CV hospitalization | 44.7 (43.7-45.6) | 48.3 (45.9-50.9) | 1.08 (1.02-1.14) | 0.006 |
| HF hospitalization | 27.3 (26.6-28.0) | 32.5 (30.7-34.4) | 1.19 (1.12-1.27) | <0.001 |
| Non-CV hospitalization | 45.1 (44.1-46.1) | 43.3 (41.0-45.8) | 0.96 (0.91-1.02) | 0.186 |
| Stroke/TIA hospitalization | 3.0 (2.8-3.2) | 2.5 (2.1-2.9) | 0.82 (0.68-0.98) | 0.03 |

**Abbreviations:** AF, atrial fibrillation; BP, blood pressure; CI, confidence interval; CV, cardiovascular; HF, heart failure; IRR, incidence rate ratio; OMT, optimal medical therapy; NYHA, New York heart association; TIA, transient ischemic attack

**Table S19. Outcomes in eligible and ineligible patients in the trial and pragmatic scenario in patients with NT-proBNP≥5,000pg/mL.**

|  | **Events per 100 patient-years (95% CI)** | |  |  |
| --- | --- | --- | --- | --- |
| **Event** | **Ineligible** | **Eligible** | ***IRR*** | ***P*** |
| **Trial scenario** | |  |  |  |
| All-cause death | 27.8 (27.1-28.5) | 29.9 (28.8-31.0) | 1.07 (1.03-1.12) | 0.002 |
| CV death | 18.6 (18.1-19.2) | 21.6 (20.7-22.6) | 1.16 (1.10-1.23) | <0.001 |
| Non-CV death | 9.2 (8.8-9.6) | 8.2 (7.6-8.8) | 0.89 (0.82-0.97) | 0.007 |
| All-cause hospitalization | 74.8 (73.0-76.6) | 87.4 (84.3-90.6) | 1.17 (1.12-1.22) | <0.001 |
| CV hospitalization | 36.5 (35.5-37.6) | 48.7 (46.8-50.7) | 1.33 (1.27-1.40) | <0.001 |
| HF hospitalization | 22.0 (21.3-22.8) | 32.9 (31.5-34.4) | 1.49 (1.41-1.58) | <0.001 |
| Non-CV hospitalization | 38.4 (37.4-39.5) | 39.1 (37.4-40.8) | 1.02 (0.97-1.07) | 0.516 |
| Stroke/TIA hospitalization | 3.0 (2.7-3.2) | 2.5 (2.2-2.8) | 0.84 (0.72-0.98) | 0.023 |
| **Pragmatic scenario** | |  |  |  |
| All-cause death | 23.4 (22.7-24.1) | 34.4 (33.5-35.3) | 1.47 (1.41-1.53) | <0.001 |
| CV death | 15.4 (14.8-16.0) | 24.4 (23.6-25.2) | 1.59 (1.51-1.67) | <0.001 |
| Non-CV death | 8.0 (7.6-8.4) | 10.0 (9.5-10.5) | 1.25 (1.16-1.35) | <0.001 |
| All-cause hospitalization | 62.5 (60.7-64.4) | 100.9 (98.2-103.7) | 1.61 (1.55-1.68) | <0.001 |
| CV hospitalization | 31.1 (30.0-32.1) | 51.8 (50.2-53.4) | 1.67 (1.59-1.74) | <0.001 |
| HF hospitalization | 17.8 (17.1-18.5) | 35.0 (33.8-36.2) | 1.97 (1.87-2.07) | <0.001 |
| Non-CV hospitalization | 33.7 (32.6-34.9) | 44.9 (43.4-46.4) | 1.33 (1.27-1.40) | <0.001 |
| Stroke/TIA hospitalization | 2.9 (2.6-3.1) | 2.8 (2.5-3.1) | 0.98 (0.86-1.13) | 0.812 |
| **Trial scenario, OMT sensitivity** | |  |  |  |
| All-cause death | 29.0 (28.4-29.6) | 24.7 (23.3-26.2) | 0.85 (0.80-0.91) | <0.001 |
| CV death | 19.8 (19.3-20.4) | 17.4 (16.2-18.7) | 0.88 (0.81-0.95) | 0.001 |
| Non-CV death | 9.2 (8.8-9.5) | 7.3 (6.5-8.2) | 0.80 (0.71-0.90) | <0.001 |
| All-cause hospitalization | 78.0 (76.3-79.6) | 80.0 (75.6-84.6) | 1.03 (0.97-1.09) | 0.398 |
| CV hospitalization | 38.7 (37.8-39.7) | 47.2 (44.4-50.2) | 1.22 (1.14-1.30) | <0.001 |
| HF hospitalization | 24.0 (23.3-24.7) | 31.7 (29.6-33.9) | 1.32 (1.23-1.42) | <0.001 |
| Non-CV hospitalization | 39.2 (38.2-40.2) | 34.9 (32.6-37.3) | 0.89 (0.83-0.96) | 0.001 |
| Stroke/TIA hospitalization | 2.9 (2.7-3.1) | 2.3 (1.8-2.8) | 0.77 (0.62-0.96) | 0.018 |
| **Trial scenario, BP sensitivity** | |  |  |  |
| All-cause death | 27.5 (26.8-28.2) | 30.6 (29.5-31.7) | 1.11 (1.06-1.16) | <0.001 |
| CV death | 18.3 (17.8-18.9) | 22.3 (21.3-23.2) | 1.22 (1.15-1.28) | <0.001 |
| Non-CV death | 9.2 (8.8-9.6) | 8.3 (7.7-8.9) | 0.90 (0.83-0.98) | 0.016 |
| All-cause hospitalization | 73.9 (72.1-75.7) | 89.6 (86.4-92.8) | 1.21 (1.16-1.27) | <0.001 |
| CV hospitalization | 36.0 (35.0-37.0) | 49.9 (48.0-51.9) | 1.39 (1.32-1.46) | <0.001 |
| HF hospitalization | 21.6 (20.9-22.3) | 33.8 (32.3-35.3) | 1.56 (1.48-1.65) | <0.001 |
| Non-CV hospitalization | 38.3 (37.2-39.4) | 39.4 (37.7-41.1) | 1.03 (0.98-1.08) | 0.281 |
| Stroke/TIA hospitalization | 3.0 (2.7-3.2) | 2.5 (2.2-2.9) | 0.86 (0.74-1.00) | 0.046 |
| **Trial scenario, AF+digoxin sensitivity** | |  |  |  |
| All-cause death | 28.1 (27.4-28.7) | 29.5 (28.4-30.8) | 1.05 (1.00-1.10) | 0.032 |
| CV death | 19.0 (18.4-19.5) | 21.2 (20.2-22.3) | 1.12 (1.06-1.18) | <0.001 |
| Non-CV death | 9.1 (8.7-9.5) | 8.3 (7.7-9.0) | 0.91 (0.84-1.00) | 0.046 |
| All-cause hospitalization | 75.9 (74.2-77.7) | 86.1 (82.7-89.6) | 1.13 (1.08-1.19) | <0.001 |
| CV hospitalization | 37.4 (36.4-38.4) | 48.1 (46.0-50.3) | 1.29 (1.22-1.36) | <0.001 |
| HF hospitalization | 22.8 (22.1-23.5) | 32.4 (30.9-34.0) | 1.42 (1.34-1.51) | <0.001 |
| Non-CV hospitalization | 38.4 (37.4-39.5) | 39.3 (37.4-41.2) | 1.02 (0.97-1.08) | 0.445 |
| Stroke/TIA hospitalization | 3.0 (2.7-3.2) | 2.4 (2.1-2.8) | 0.81 (0.68-0.96) | 0.011 |
| **Trial scenario, AF sensitivity** | |  |  |  |
| All-cause death | 29.1 (28.5-29.7) | 23.4 (21.9-25.0) | 0.80 (0.75-0.86) | <0.001 |
| CV death | 19.9 (19.4-20.4) | 16.8 (15.5-18.1) | 0.84 (0.78-0.92) | <0.001 |
| Non-CV death | 9.2 (8.9-9.6) | 6.6 (5.8-7.5) | 0.72 (0.63-0.82) | <0.001 |
| All-cause hospitalization | 80.0 (78.3-81.7) | 64.8 (60.7-69.1) | 0.81 (0.76-0.87) | <0.001 |
| CV hospitalization | 40.2 (39.2-41.1) | 36.1 (33.6-38.8) | 0.90 (0.83-0.97) | 0.005 |
| HF hospitalization | 24.9 (24.2-25.6) | 24.6 (22.7-26.6) | 0.99 (0.91-1.07) | 0.783 |
| Non-CV hospitalization | 39.6 (38.6-40.5) | 31.4 (29.1-33.9) | 0.79 (0.73-0.86) | <0.001 |
| Stroke/TIA hospitalization | 3.0 (2.7-3.2) | 1.9 (1.5-2.4) | 0.65 (0.50-0.83) | <0.001 |
| **Pragmatic scenario, BP sensitivity** | |  |  |  |
| All-cause death | 22.6 (21.9-23.3) | 35.0 (34.1-35.9) | 1.55 (1.48-1.61) | <0.001 |
| CV death | 14.8 (14.2-15.3) | 24.9 (24.1-25.7) | 1.69 (1.60-1.78) | <0.001 |
| Non-CV death | 7.9 (7.5-8.3) | 10.1 (9.6-10.6) | 1.28 (1.19-1.38) | <0.001 |
| All-cause hospitalization | 60.7 (58.9-62.5) | 102.9 (100.2-105.7) | 1.70 (1.63-1.77) | <0.001 |
| CV hospitalization | 30.0 (29.0-31.1) | 52.8 (51.2-54.4) | 1.76 (1.68-1.84) | <0.001 |
| HF hospitalization | 17.0 (16.3-17.7) | 35.7 (34.6-36.9) | 2.10 (1.99-2.22) | <0.001 |
| Non-CV hospitalization | 33.2 (32.1-34.4) | 45.2 (43.8-46.7) | 1.36 (1.30-1.43) | <0.001 |
| Stroke/TIA hospitalization | 2.8 (2.6-3.1) | 2.8 (2.6-3.1) | 1.00 (0.88-1.15) | 0.946 |
| **Pragmatic scenario, AF+digoxin sensitivity** | |  |  |  |
| All-cause death | 24.7 (24.0-25.4) | 34.5 (33.4-35.5) | 1.39 (1.34-1.45) | <0.001 |
| CV death | 16.6 (16.0-17.2) | 24.3 (23.4-25.2) | 1.46 (1.39-1.54) | <0.001 |
| Non-CV death | 8.1 (7.7-8.5) | 10.2 (9.6-10.8) | 1.25 (1.16-1.35) | <0.001 |
| All-cause hospitalization | 66.3 (64.6-68.1) | 101.3 (98.3-104.4) | 1.53 (1.47-1.59) | <0.001 |
| CV hospitalization | 33.2 (32.2-34.2) | 51.9 (50.2-53.7) | 1.56 (1.49-1.64) | <0.001 |
| HF hospitalization | 19.6 (18.9-20.3) | 34.9 (33.7-36.3) | 1.78 (1.69-1.88) | <0.001 |
| Non-CV hospitalization | 34.8 (33.7-35.9) | 45.4 (43.8-47.1) | 1.31 (1.25-1.37) | <0.001 |
| Stroke/TIA hospitalization | 2.9 (2.7-3.1) | 2.7 (2.4-3.0) | 0.94 (0.82-1.08) | 0.384 |
| **Pragmatic scenario, AF sensitivity** | |  |  |  |
| All-cause death | 28.6 (28.0-29.3) | 27.5 (26.1-28.8) | 0.96 (0.91-1.01) | 0.131 |
| CV death | 19.6 (19.1-20.2) | 18.9 (17.8-20.1) | 0.96 (0.90-1.03) | 0.293 |
| Non-CV death | 9.0 (8.6-9.4) | 8.5 (7.8-9.3) | 0.95 (0.86-1.04) | 0.263 |
| All-cause hospitalization | 78.4 (76.7-80.1) | 77.5 (73.8-81.4) | 0.99 (0.94-1.04) | 0.695 |
| CV hospitalization | 39.9 (38.9-40.9) | 38.9 (36.8-41.1) | 0.97 (0.92-1.04) | 0.4 |
| HF hospitalization | 24.6 (23.9-25.3) | 26.4 (24.8-28.1) | 1.07 (1.00-1.15) | 0.04 |
| Non-CV hospitalization | 39.0 (38.0-40.0) | 36.7 (34.6-38.9) | 0.94 (0.88-1.00) | 0.066 |
| Stroke/TIA hospitalization | 3.0 (2.8-3.2) | 2.1 (1.7-2.5) | 0.69 (0.57-0.85) | <0.001 |

**Abbreviations:** AF, atrial fibrillation; BP, blood pressure; CI, confidence interval; CV, cardiovascular; HF, heart failure; IRR, incidence rate ratio; NT-proBNP, N-terminal pro-B-type natriuretic peptide; OMT, optimal medical therapy; TIA, transient ischemic attack

**Table S20. Outcomes in eligible and ineligible patients in the trial and pragmatic scenario in the complete case analysis.**

|  | **Events per 100 patient-years (95% CI)** | |  |  |
| --- | --- | --- | --- | --- |
| **Event** | **Ineligible** | **Eligible** | ***IRR*** | ***P*** |
| **Trial scenario** |  |  |  |  |
| All-cause death | 14.9 (14.5-15.3) | 22.6 (21.5-23.7) | 1.52 (1.43-1.60) | <0.001 |
| CV death | 9.4 (9.1-9.7) | 15.8 (14.9-16.7) | 1.68 (1.57-1.80) | <0.001 |
| Non-CV death | 5.5 (5.3-5.8) | 6.8 (6.2-7.4) | 1.24 (1.12-1.37) | <0.001 |
| All-cause hospitalization | 47.1 (46.0-48.1) | 70.7 (67.7-73.8) | 1.50 (1.43-1.58) | <0.001 |
| CV hospitalization | 23.9 (23.3-24.6) | 41.1 (39.2-43.1) | 1.72 (1.63-1.81) | <0.001 |
| HF hospitalization | 14.3 (13.8-14.7) | 29.2 (27.7-30.8) | 2.05 (1.93-2.18) | <0.001 |
| Non-CV hospitalization | 26.7 (26.0-27.4) | 33.7 (32.0-35.5) | 1.26 (1.19-1.34) | <0.001 |
| Stroke/TIA hospitalization | 2.2 (2.0-2.3) | 2.2 (1.9-2.6) | 1.02 (0.85-1.22) | 0.788 |
| **Pragmatic scenario** |  |  |  |  |
| All-cause death | 12.6 (12.2-13.1) | 26.1 (25.2-27.1) | 2.07 (1.97-2.17) | <0.001 |
| CV death | 7.8 (7.5-8.2) | 17.8 (17.1-18.7) | 2.28 (2.14-2.42) | <0.001 |
| Non-CV death | 4.8 (4.5-5.1) | 8.3 (7.7-8.8) | 1.72 (1.58-1.88) | <0.001 |
| All-cause hospitalization | 42.0 (41.0-43.1) | 80.8 (78.1-83.5) | 1.92 (1.84-2.00) | <0.001 |
| CV hospitalization | 21.1 (20.4-21.7) | 44.8 (43.1-46.5) | 2.13 (2.03-2.23) | <0.001 |
| HF hospitalization | 12.0 (11.6-12.5) | 31.7 (30.4-33.0) | 2.63 (2.49-2.78) | <0.001 |
| Non-CV hospitalization | 24.4 (23.7-25.1) | 38.5 (37.0-40.1) | 1.58 (1.50-1.66) | <0.001 |
| Stroke/TIA hospitalization | 2.1 (1.9-2.2) | 2.5 (2.2-2.8) | 1.20 (1.03-1.39) | 0.019 |

**Abbreviations:** CI, confidence interval; CV, cardiovascular; HF, heart failure; IRR, incidence rate ratio; TIA, transient ischemic attack

**Table S21. Outcomes in eligible and ineligible patients in the trial and pragmatic scenario in the missing-as-eligible analysis.**

|  | **Events per 100 patient-years (95% CI)** | |  |  |
| --- | --- | --- | --- | --- |
| **Event** | **Ineligible** | **Eligible** | ***IRR*** | ***P*** |
| **Trial scenario** |  |  |  |  |
| All-cause death | 18.3 (18.0-18.6) | 23.9 (23.2-24.5) | 1.30 (1.26-1.35) | <0.001 |
| CV death | 11.7 (11.4-11.9) | 16.8 (16.2-17.3) | 1.43 (1.38-1.49) | <0.001 |
| Non-CV death | 6.6 (6.4-6.8) | 7.1 (6.8-7.5) | 1.08 (1.01-1.14) | 0.014 |
| All-cause hospitalization | 52.0 (51.2-52.8) | 72.7 (70.8-74.6) | 1.40 (1.36-1.44) | <0.001 |
| CV hospitalization | 25.6 (25.1-26.0) | 39.2 (38.1-40.3) | 1.53 (1.48-1.59) | <0.001 |
| HF hospitalization | 14.5 (14.2-14.8) | 25.7 (24.9-26.5) | 1.77 (1.70-1.84) | <0.001 |
| Non-CV hospitalization | 29.6 (29.1-30.1) | 35.4 (34.3-36.5) | 1.20 (1.15-1.24) | <0.001 |
| Stroke/TIA hospitalization | 2.3 (2.2-2.5) | 2.4 (2.1-2.6) | 1.01 (0.91-1.11) | 0.917 |
| **Pragmatic scenario** |  |  |  |  |
| All-cause death | 15.0 (14.7-15.4) | 28.2 (27.6-28.8) | 1.87 (1.82-1.93) | <0.001 |
| CV death | 9.4 (9.2-9.7) | 19.3 (18.9-19.8) | 2.05 (1.98-2.13) | <0.001 |
| Non-CV death | 5.6 (5.4-5.8) | 8.8 (8.5-9.2) | 1.57 (1.50-1.65) | <0.001 |
| All-cause hospitalization | 44.6 (43.8-45.3) | 84.5 (82.8-86.2) | 1.90 (1.85-1.95) | <0.001 |
| CV hospitalization | 22.0 (21.5-22.4) | 42.7 (41.8-43.7) | 1.95 (1.89-2.01) | <0.001 |
| HF hospitalization | 11.9 (11.6-12.2) | 27.9 (27.2-28.6) | 2.35 (2.27-2.44) | <0.001 |
| Non-CV hospitalization | 26.0 (25.5-26.5) | 41.4 (40.4-42.3) | 1.59 (1.54-1.64) | <0.001 |
| Stroke/TIA hospitalization | 2.2 (2.1-2.3) | 2.6 (2.5-2.8) | 1.21 (1.11-1.32) | <0.001 |

**Abbreviations:** CI, confidence interval; CV, cardiovascular; HF, heart failure; IRR, incidence rate ratio; TIA, transient ischemic attack

**Table S22. Comparison of patient characteristics in patients eligible for GALACTIC-HF in SwedeHF, vs. the GALACTIC-HF trial population.**

| **Event** | **SwedeHF patients with EF<40% fulfilling GALACTIC-HF criteria** | **GALACTIC-HF** |
| --- | --- | --- |
|  |  |  |
| Age, years (mean) | 74 | 65 |
| Female | 27% | 21% |
| In-patients | 46% | 25% |
| Atrial fibrillation | 63% | 42% |
| Diabetes mellitus | 39% | 40% |
| Ischemic heart disease | 69% | 62% |
| Hypertension | 65% | 70% |
| Chronic obstructive pulmonary disease | 19% | 16% |
| NYHA class III-IV | 63% | 47% |
| Body mass index, kg/m^2^ (mean) | 27 | 29 |
| Systolic blood pressure, mmHg (mean) | 115 | 117 |
| Heart rate, b.p.m. (mean) | 74 | 72 |
| NT-proBNP, pg/mL (median) | 4,250 | 1,971 |
| eGFR, mL/min/1.73 m^2^ (median) | 56 | 59 |
| RASi/ARNi | 90% | 87% |
| Beta-blocker | 93% | 94% |
| MRA | 54% | 77% |
| RASi/ARNi + Beta-blocker + MRA | 47% | 65% |
| Cardiac resynchronisation therapy | 16% | 14% |
| Implantable cardioverter-defibrillator | 17% | 32% |

**Abbreviations:** ARNi, angiotensin receptor–neprilysin inhibitor; b.p.m., beats per minute; eGFR, estimated glomerular filtration rate (calculated by the Chronic Kidney Disease Epidemiology Collaboration formula); GALACTIC-HF, Global Approach to Lowering Adverse Cardiac Outcomes through Improving Contractility in Heart Failure; MRA, mineralocorticoid receptor antagonist; NT-proBNP, N-terminal pro-B-type natriuretic peptide; NYHA, New York Heart Association; RASi, renin–angiotensin system inhibitor; SwedeHF, Swedish Heart Failure registry

**Figure S1. Patient selection flowchart.**


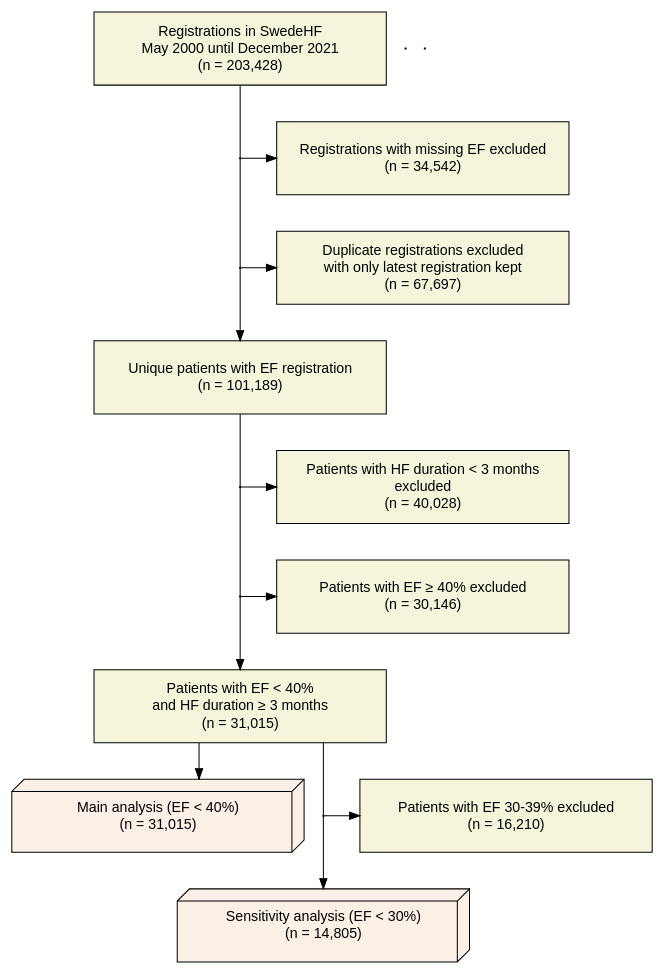


**Abbreviations:** EF, ejection fraction; HF, heart failure; SwedeHF, Swedish heart failure registry
